# Spatially Refined Time-Varying Reproduction Numbers of COVID-19 by Health District in Georgia, USA, March–December 2020

**DOI:** 10.3390/epidemiologia2020014

**Published:** 2021-05-28

**Authors:** Chigozie A. Ogwara, Arshpreet Kaur Mallhi, Xinyi Hua, Kamalich Muniz-Rodriguez, Jessica S. Schwind, Xiaolu Zhou, Jeffery A. Jones, Joanne Chopak-Foss, Gerardo Chowell, Isaac Chun-Hai Fung

**Affiliations:** 1 Department of Biostatistics, Epidemiology and Environmental Health Sciences, Jiann-Ping Hsu College of Public Health, Georgia Southern University, Statesboro, GA 30460, USA; co05814@georgiasouthern.edu (C.A.O.); am39588@georgiasouthern.edu (A.K.M.); xh00279@georgiasouthern.edu (X.H.); km11200@georgiasouthern.edu (K.M.-R.); jschwind@georgiasouthern.edu (J.S.S.); 2 Department of Geography, AddRan College of Liberal Arts, Texas Christian University, Fort Worth, TX 76109, USA; xiaolu.zhou@tcu.edu; 3 Department of Health Policy and Community Health, Jiann-Ping Hsu College of Public Health, Georgia Southern University, Statesboro, GA 30460, USA; jajones@georgiasouthern.edu (J.A.J.); jchopak@georgiasouthern.edu (J.C.-F.); 4 Department of Population Health Sciences, School of Public Health, Georgia State University, Atlanta, GA 30302, USA; gchowell@gsu.edu

**Keywords:** COVID-19, SARS-CoV-2, daily incidence, reproduction number, Georgia

## Abstract

This study quantifies the transmission potential of SARS-CoV-2 across public health districts in Georgia, USA, and tests if per capita cumulative case count varies across counties. To estimate the time-varying reproduction number, *R_t_* of SARS-CoV-2 in Georgia and its 18 public health districts, we apply the R package ‘EpiEstim’ to the time series of historical daily incidence of confirmed cases, 2 March–15 December 2020. The epidemic curve is shifted backward by nine days to account for the incubation period and delay to testing. Linear regression is performed between log_10_-transformed per capita cumulative case count and log_10_-transformed population size. We observe *R_t_* fluctuations as state and countywide policies are implemented. Policy changes are associated with increases or decreases at different time points. *R_t_* increases, following the reopening of schools for in-person instruction in August. Evidence suggests that counties with lower population size had a higher per capita cumulative case count on June 15 (slope = −0.10, *p* = 0.04) and October 15 (slope = −0.05, *p* = 0.03), but not on August 15 (slope = −0.04, *p* = 0.09), nor December 15 (slope = −0.02, *p* = 0.41). We found extensive community transmission of SARS-CoV-2 across all 18 health districts in Georgia with median 7-day-sliding window *R_t_* estimates between 1 and 1.4 after March 2020.

## 1. Introduction

By 15 December 2020, the United States (U.S.) had reported 16,545,464 confirmed coronavirus disease 2019 (COVID-19) cases, 301,264 deaths and 6,298,082 recoveries [1], since its first laboratory-confirmed case on 20 January 2020 [2]. The COVID-19 pandemic, caused by the severe acute respiratory syndrome coronavirus 2 (SARS-CoV-2), has had a significant impact on the general public’s physical and mental well-being and financial status [3,4,5,6]. These challenges influenced and transformed both individual behavior and government policies. Common symptoms of COVID-19 include fever, cough, myalgia, and fatigue, but the risk of severe disease is contingent on age, sex, and the presence of any underlying medical conditions [7,8]. COVID-19 primarily spreads through the respiratory tract, by droplets, respiratory secretions, and direct contact [9]. Studies reported that the median age of COVID-19 patients was 47–56 years, and 42–46% of patients were females [10,11,12]. In the state of Georgia, the median age of the general population is 37.2 years, 57.8% are of the white race, and 51.3% females [13,14].

With growing pressure from increasing unemployment rates and a real possibility of an economic recession [15,16,17], many state governments lifted or relaxed lockdown measures. On 14 March, the state of Georgia declared a public health state of emergency, which was followed by school closures. A statewide shelter-in-place executive order was in place on 2 April. By 27 April, businesses in Georgia started to reopen with social distancing and prevention measures in place. Georgia was one of the few states first to open up after closures in April (Table 1).

**Figure 1:**
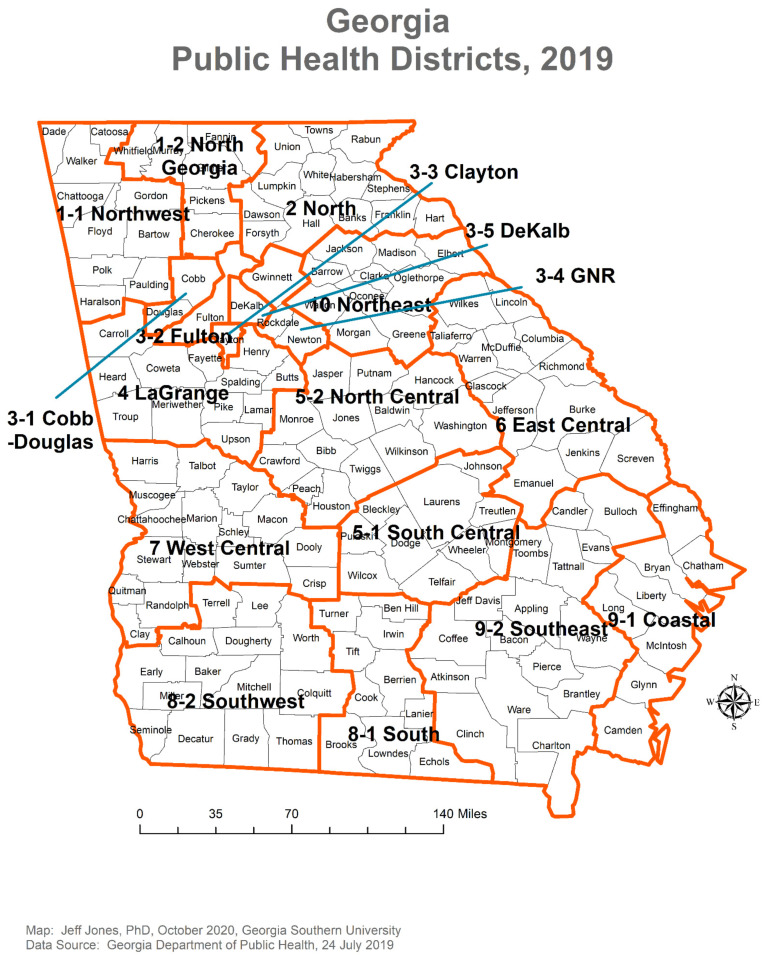
Public health districts in Georgia, USA, 2019.

There are 159 counties in Georgia, which are grouped into 18 public health districts (Figure 1) [18]. Public health districts are administrative extensions of the Georgia Department of Public Health (GDPH) and serve as local public health agencies in Georgia [18]. They vary by their population sizes and the intensities of public health interventions against COVID-19. Hence, an analysis of the changing transmission potential of SARS-CoV-2 over time in each of the 18 public health districts will shed light on the development of the pandemic in Georgia.

According to the GDPH, Georgia reported its first case on 2 March 2020. Similar to other Sunbelt states, Georgia experienced a summer surge in cases starting in late June, with daily increases peaking on 24 July at 4792 new confirmed cases. By October 2020, Georgia had collected almost 3 million SARS-CoV-2 polymerase chain reaction (PCR) tests for active infections. Confirmed cases had more than doubled since July to 318,026, including 7021 fatalities. By the end of October, all 159 Georgia counties have reported COVID-19 cases; all but one county, Taliaferro, have reported COVID-19 deaths. While the number of confirmed cases was concentrated in the Atlanta metropolitan area, rural Chattahoochee County reported the highest case rate at 15,499 per 100,000 population. Small, rural Hancock County (population 8193) similarly had the highest death rate at 512.6 per 100,000. In the early months of the pandemic, the majority of deaths were among African-American Georgians. By October, 53% of fatalities were among White Georgians over the age of 60. While African-Americans make up 31% of Georgia’s population, this group disproportionately accounts for 41% of COVID-19 deaths. By comparison, the typical confirmed case by October was a White female aged 18–29 with unknown co-morbidities [19].

To assess how the transmissibility of the virus changed over time, given the public health interventions and behavioral changes in place, we computed the time-varying reproduction number, denoted as *R_t_*, of COVID-19 for Georgia and each of its public health districts. To illustrate the differences in the impact of policy change on patterns of SARS-CoV-2 transmission between health districts, District 3-2 (Fulton County) and District 8-2 (South West) were highlighted in the main text. These two districts were chosen because of a difference in the impact of policies on the *R_t_* estimates. Details of the remaining public health districts are provided in the Online Supplementary Materials. To assess the power-law relationship between population size and cumulative COVID-19 case count on 15 June, 15 August, 15 October, and 15 December 2020, we performed a linear regression between the log_10_-transformed population size and log_10_-transformed cumulative COVID-19 case count, by health district and by county, respectively.

## 2. Materials and Methods

This study used historical data from the COVID-19 pandemic, 2 March–15 December 2020, in the state of Georgia and its 18 health districts. A detailed list and description of all counties within the 18 health districts are provided in Table 2.

### 2.1. Data Acquisition

The cumulative case count data of confirmed cases were downloaded from 2 March to 15 December 2020, for the entire state of Georgia and for each county in its 18 health districts from the New York Times (NYT) GitHub data repository [33]. Each jurisdiction included in our study has a start date corresponding to the first reported case for the area, according to NYT (Table 2). The first case in Georgia was reported on 2 March 2020 [33]. Our cutoff point for all jurisdictions was 15 December 2020, 7 months and 18 days after businesses in the state reopened on 27 April 2020, following the guidelines of social distancing and prevention measures, including the wearing of face coverings (Table 1) [20]. We verified the numbers with official statistical reports from the GDPH [18,19]. If any inconsistencies were found, the numbers from GDPH were used as the standard. During the data cleaning process, we discovered a major backlog event on 5 October 2020, with 21,349 confirmed cases [34]. We adjusted this single-day spike by evenly dividing the case count reported on 5 October 2020 into 31 portions. We kept two portions for 5 October 2020 and added the rest of 29 portions to the previous 29 days, respectively. We rounded down the number and added the remainder to 5 October 2020. All the calculations had been done using R. The management of negative incident case counts is described in Appendix A. We merged the county-level data from NYT to obtain the health district-level data. To estimate *R_t_*, we calculated the daily number of new cases based on the daily cumulative case counts reported. We also searched local government web pages to verify if any control measures were established. Such information is presented in Table 1 and Table S1 in the Online Supplementary Materials. We accessed 2019 total county population data from the U.S. Census Bureau and obtained an estimate for the power-law relationship between cumulative case count and population size [35].

### 2.2. Statistical Analysis

Time-varying reproduction number, also known as the instantaneous reproduction number (denoted as *R_t_*), was calculated by the R package ‘EpiEstim’ version 2.2-3 [36]. This measure was defined by Cori et al. [36] as the ratio between *I_t_*, the number of incident cases at the time *t*, to the total infectiousness of all persons with COVID-19 at the time *t*. We briefly describe this established statistical method below in Appendix B. For generating *R_t_* by assumed date of infection, we shifted nine days backward (assuming six days of the average incubation period plus three days mean delay of testing) according to Gostic et al. [37]. Besides using the default 7-day sliding window from the EpiEstim package, we also analyze *R_t_* by the different nonoverlapping time periods when different nonpharmaceutical interventions have been implemented in the state of Georgia (we call them policy change *R_t_*). We highlighted five major policies relating to COVID-19 in Georgia with dates: 18 March 2020—Closing of public elementary, secondary, and postsecondary schools; 23 March 2020—Limiting large gatherings statewide shelter in place for the vulnerable and closing of bars/nightclubs; 2 April 2020—Statewide shelter in place “for everyone”; 20 April 2020—Certain business moved to minimum operations; 5 August 2020—Schools began reopening. We estimated the time-varying reproduction number *R_t_* and policy change *R_t_* at the state and public health district levels. For the time windows taking the average of *R_t_*, we used a 7-day sliding window and a nonoverlapping window (policy change *R_t_*), respectively. We specified the serial interval (mean = 4.60 days; standard deviation = 5.55 days) according to You et al. [38].

Through bootstrapping, we calculated the percentage change for the nonoverlapping window *R_t_* and its 95% credible interval for the state of Georgia, and Public Health District 3-2 and 8-2. The policy change *R_t_* estimate at each policy interval will be compared to the previous policy interval, i.e., $\frac{t2-t1}{t1}$. We drew 1000 random numbers from each distribution of policy change *R_t_* estimate at each policy interval, so that the 95% credible interval can be calculated. We also calculated the percentage change of the nonoverlapping window *R_t_* for the rest of the 16 Districts (Table S2–S20).

We characterized the power-law relationship between the per capita cumulative number of COVID-19 cases and population size, following C~N^g^ (C, cumulative case count; N, population size; g, exponent) [39]. We performed a linear regression between the log_10_-transformed per capita cumulative case count and the log_10_-transformed population size, i.e., log_10_(C/N) = m log_10_(N) where m = g − 1 [39]. We conducted linear regression between the log_10_-transformed per capita cumulative case count and the log_10_-transformed population size, at four different dates: 15 June, 15 August, 15 October, and 15 December.

Per capita cumulative case count would be exactly proportional to population size, and there was no heterogeneity of per capita cumulative case count across geographic units of different population sizes if m = 0 (i.e., g = 1). Geographical units with lower population sizes would have a higher per capita cumulative case count if m < 0 (i.e., g < 1) and lower per capita cumulative case count if m > 0 (i.e., g > 1) [39]. 

**Figure 2:**
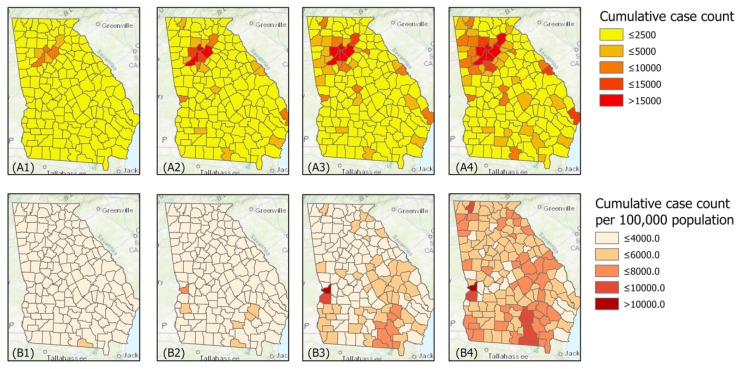
Maps of Georgia’s counties by cumulative case count (**top**), and cumulative case counts per 100,000 population (**bottom**) on 15 June, 15 August, 15 August, and 15 December 2020.

Statistical analysis was performed using R 3.6.2 to 4.0.0 (R Core Team, R Foundation for Statistical Computing, Vienna, Austria). Figure 1 was created using ArcMap 10.6 (Esri, Redlands, CA, USA). Figure 2 was created using ArcGIS Pro Version 2.4.0 (Esri, Redlands, CA, USA), with color codes arranged according to quintiles of the values. 

### 2.3. Ethics

The Georgia Southern University Institutional Review Board made a nonhuman subjects determination for this project (H20364) under the G8 exemption category.

## 3. Results

As of 15 December 2020, there were a cumulative total of 534,488 confirmed COVID-19 cases in the state of Georgia. Figure 2 presents the spatial variation of cumulative case count, the population size, and the cumulative case count per 100,000 population by county in Georgia, over time at four time points: 15 June; 15 August; 15 October; and 15 December 2020. While there have been many cases in the Atlanta metropolitan area, this area is also the population hub of Georgia. By 15 December 2020, a high incidence rate was observed in rural Georgia counties. The counties with the highest cumulative case count (as of 15 December 2020) were those with the largest population sizes. The counties with the highest cumulative case count per 100,000 population were those with the smallest population sizes (Figure 2).

### 3.1. R_t_ Estimates

**Figure 3:**
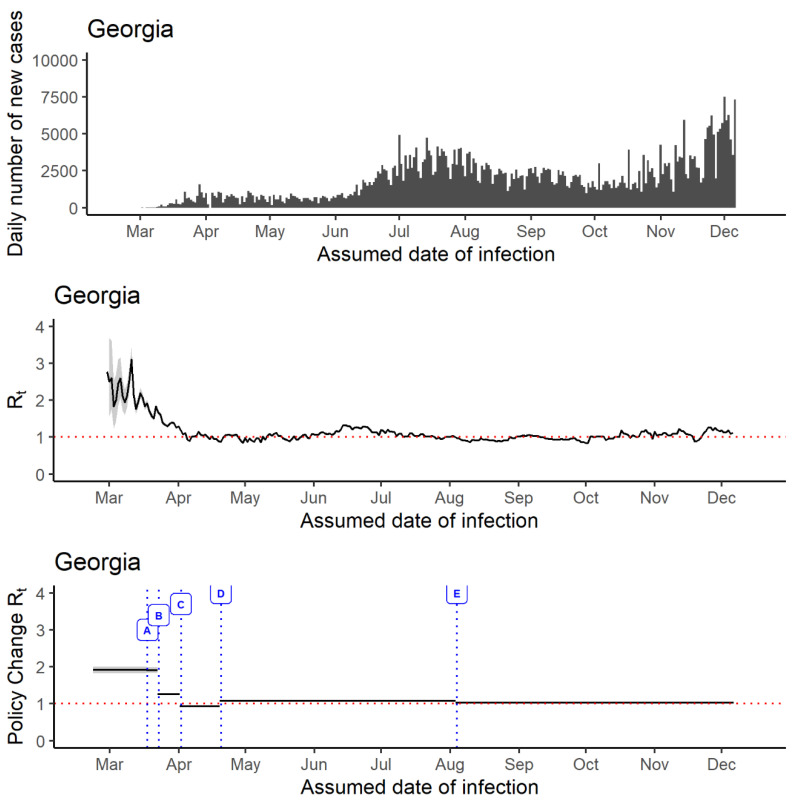
The daily number of incident cases (**upper panel**) in Georgia, USA, 2 March—15 December 2020, and *R_t_* estimated using the instantaneous reproduction number method implemented in the ‘EpiEstim’ package (**middle panel**: 7-day sliding window; lower panel: policy change *R_t_*). Policy A: School closures (18 March 2020). Policy B: Limiting large gatherings statewide shelter in place for vulnerable and closing bars/nightclubs for 14 days (23 March 2020) Policy C: Statewide shelter in place for everyone (2 April 2020) Policy D: Minimum operations among certain businesses (20 April 2020) Policy E: Reopening of schools in Georgia (5 August 2020).

From 2 March to 15 December 2020, Georgia’s daily incidence data show an extensive community transmission of SARS-CoV-2 with a surge of new cases in late-June and July (Figure 3). As of 15 December 2020, the median *R_t_* estimates of 1.13 (95% credible interval, CrI, 1.13, 1.14) were observed. The median 7-day sliding window *R_t_* estimates for every health district in Georgia were more than 1 for 15 December. The lowest median *R_t_* of 1.07 (95% CrI, 1.00, 1,11) was observed in District 3-2 (Fulton Board of Health), and the highest median *R_t_* of 1.25 (95% CrI, 1.14, 1.37) in District 5-1 (South Central Health District) (Table 2). As of 15 December, every district in Georgia demonstrated evidence of SARS-CoV-2 transmission: No districts showed the median 7-day sliding window *R_t_* below 1 (i.e., a sign of decline in the epidemic).

Social distancing measures were encouraged in Georgia, since the first case was reported in the state. The median 7-day sliding window *R_t_* estimate in Georgia was observed between 2 and 3 in mid-March. It remained below 2 from late-March to early-April, and then fluctuated around 1 afterward. The median 7-day sliding window *R_t_* estimate dropped below 1 for the first time in early-April. 7-day sliding window *R_t_* oscillated around 1 from early-April to early-June, and then increased to >1 and stayed above 1 until mid-July, and then fluctuated around 1 until mid-December (Figure 3). 

The median policy change *R_t_* estimates trend demonstrated a high *R_t_* during the initial phase of the pandemic, which reduced to around 1 after implementing nonpharmaceutical interventions (Table S2). After the implementation of the ‘school closure policy’ (Policy A), the policy change *R_t_* remained around 2 (median *R_t_* difference percentage: −0.14%, 95% CrI, −3.94%, 3.59%). ‘Limiting large gatherings statewide shelter in place for vulnerable and closing bars/nightclubs for 14 days’ (Policy B) impacted the policy change *R_t_* estimate significantly as it helped in reducing the policy change *R_t_* estimate below 1.5 (median *R_t_* difference percentage: −34.21%, 95% CrI, −33.1%, 35.3%). The state of Georgia observed a policy change *R_t_* estimate slightly below 1 (median *R_t_* difference percentage: −25.46%, 95% CrI, −25.2%, −25.8%) after the ‘Statewide shelter in place for everyone’ (Policy C) was imposed whereas, ‘minimum operations among certain businesses’ (Policy D) led to an increase in the policy change *R_t_* estimate above 1 (median *R_t_* difference percentage: 14.35%, 95% CrI, 12.2%, 16.3%). Reopening of schools in Georgia (Policy E: 5 August, schools began reopening for in-person instruction with virtual options offered) seemed to have a little impact on the policy change *R_t_* estimate (median *R_t_* difference percentage: −3.99%, 95% CrI, −3.9%, −4.1%). 

For the majority of the districts (Figures S1–S18 in the Online Supplementary Materials), the median 7-day sliding window *R_t_* estimates fluctuated above and below 1.5 in March and gradually decreased to around 1 in April. District 9-2 was the only district that experienced a median 7-day sliding window *R_t_* estimate as high as 10 in March (Figure S17). The measures continued to fluctuate around 1 until 15 December. For District 2 (North Health District), the median 7-day sliding window *R_t_* estimates fluctuated above 1 until late-April and then gradually decreased to around 1, and continued to decrease and reached the lowest median 7-day sliding window *R_t_* of approximately 0.5 in early-May. Following this time period, it gradually increased to around 1 in mid-May and fluctuated around 1 until 15 December (Figure S3). District 2 observed the lowest median 7-day sliding window *R_t_* (i.e., ≈ 0.5) in early-May, which showed that the transmission of infection was in a declining phase in May. Easing COVID-19 restrictions may have led to an increase in the 7-day sliding window *R_t_* magnitude later.

Policy changes impacted most districts in a similar manner. For example, after the implementation of the ‘school closure policy’ (Policy A), the policy change *R_t_* estimate remained below 2 for the majority of the district, whereas it remained above 2 in Districts 2, 3-3, 3-4, 8-2,9-1, 9-2, and above 3 in Districts 6 and 7 (Supplementary Tables S3–S20). All districts observed the policy change *R_t_* estimate between 1 and 1.56 after the implementation of ‘Limiting large gatherings statewide shelter in place for vulnerable and closing bars/nightclubs for 14 days’ (Policy B), whereas District 1-1 observed the policy change *R_t_* estimate slightly below 1. ‘Statewide shelter in place for everyone’ (Policy C) seemed to help reduce the policy change *R_t_* estimates in most of the district. A significant impact of Policy C was observed in Districts 3-1, 3-2, 3-3, 3-5, 4, 6, 7, 8-1, 8-2, 9-1, and 9-2 because these districts observed a policy change *R_t_* estimate almost equal to 1 or below 1. Districts 1-1, 3-1, 3-2, 3-3, 3-5, 4, 6, 7, 8-1, 8-2, 9-1, and 9-2 experienced an increase in the policy change *R_t_* estimate after the implementation of ‘minimum operations among certain businesses’ (Policy D); District 10 did not observe any change, whereas the rest of districts observed a decrease in the policy change *R_t_* estimate. Reopening of schools in Georgia (Policy E) did not significantly impact the policy change *R_t_* estimate; moreover, it led to a further decrease in policy change *R_t_* estimate to approximately 1 in some districts.

### 3.2. Comparison between Districts That Experienced Early and Late Arrival of Highest R_t_ Magnitude

**Figure 4:**
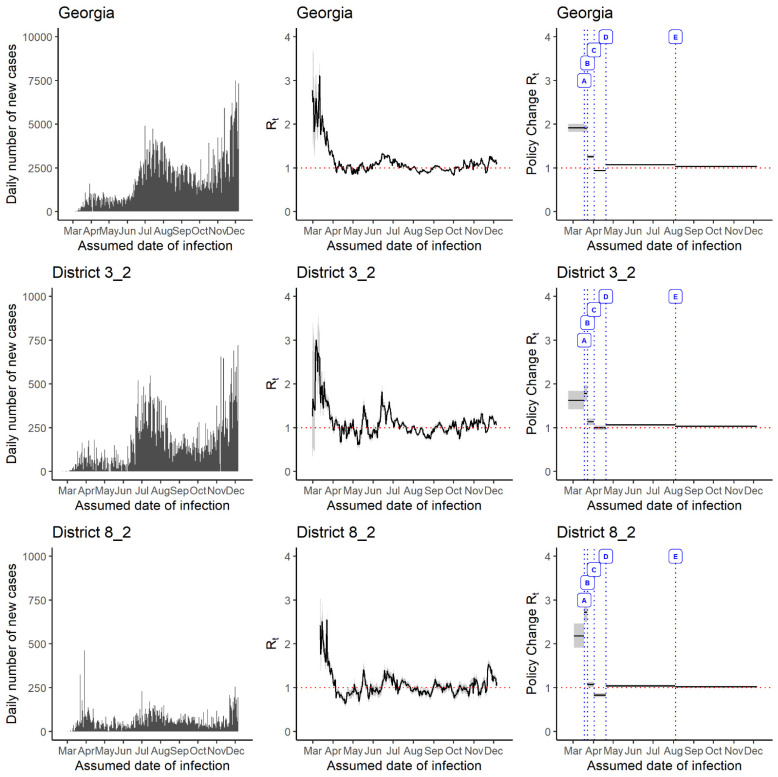
Daily number of incident cases (**left panel**), and time-varying reproduction number (*R_t_*) in 7-day sliding window (**middle panel**), and per policy change (**right panel**) in Georgia, Public Health District 3-2 (Fulton), and Public Health District 8-2, 2 March–15 December 2020, estimated using the instantaneous reproduction number method implemented in the ‘EpiEstim’ package. Policy A: School closures (18 March 2020). Policy B: Limiting large gatherings statewide shelter in place for vulnerable and closing bars/nightclubs for 14 days (23 March 2020) Policy C: Statewide shelter in place for everyone (2 April 2020) Policy D: Minimum operations among certain businesses (20 April 2020) Policy E: Reopening of schools in Georgia (5 August 2020).

As examples to further illustrate our points, we compared District 3-2 (Fulton) to District 8-2 (Southwest) (Figure 4). District 3-2 experienced an early arrival of the highest 7-day sliding window *R_t_* magnitude (i.e., in mid-March) for estimates. The 7-day sliding window *R_t_* estimate fluctuated around 2 until late-March and reached around 1 in early-April. It then continued to oscillate around 1 until mid-June and mostly remained above 1 until mid-July and then fluctuated around 1 until 15 December. 

After the implementation of the ‘school closure policy’ (Policy A), the policy change *R_t_* estimate remained below 2 (median *R_t_* difference percentage: 9.89%, 95% CrI, 1.52%, 18.17%). ‘Limiting large gatherings statewide shelter in place for vulnerable closing bars/nightclubs for 14 days’ (Policy B) and ‘Statewide shelter in place for everyone’ (Policy C) seemed to help reduce the policy change *R_t_* value to around 1 (median *R_t_* difference percentage: (−36.58, 95% CrI, −29.8, −43.0) & (−12.13, 95% CrI, −4.4, −19.6) for policy B and C, respectively). Whereas, the policy change *R_t_* becomes slightly above 1 (median *R_t_* difference percentage: 6.57, 95% CrI,1.33, 12.02), after the implementation of ‘minimum operations among certain businesses’ (Policy D), and a slight increase in the policy change *R_t_* estimate was observed after ‘schools began reopening in Georgia’ [Policy E: (median *R_t_* difference percentage: −2.95, 95% CrI, −2.72, −3.19)]. 

The policy change *R_t_* estimate for District 8-2 followed a trend similar to the policy change *R_t_* estimate of District 3-2. ‘Policy A’ kept the policy change *R_t_* estimate below 3 (median *R_t_* difference percentage: 25.06, 95% CrI, 8.98, 41.68). ‘Policy B’ seemed to significantly impact the policy change *R_t_* estimate and bring it down to around 1 (median *R_t_* difference percentage: −60.76, 95% CrI, −56.7, −64.5). After the implementation of policy C, the policy change *R_t_* estimate decreased to below 1 (median *R_t_* difference percentage: –22.97, 95% CrI, −22.9, −23.0). Whereas, Policy D and E led to an increase in policy change *R_t_* estimate slightly above 1 (median *R_t_* difference percentage: (25.92, 95% CrI, 20.4, 30.8) & (−2.01, 95% CrI, −1.73, −2.28), respectively).

### 3.3. Power-Law Relationship between Cumulative Case Count and Population Size

**Figure 5:**
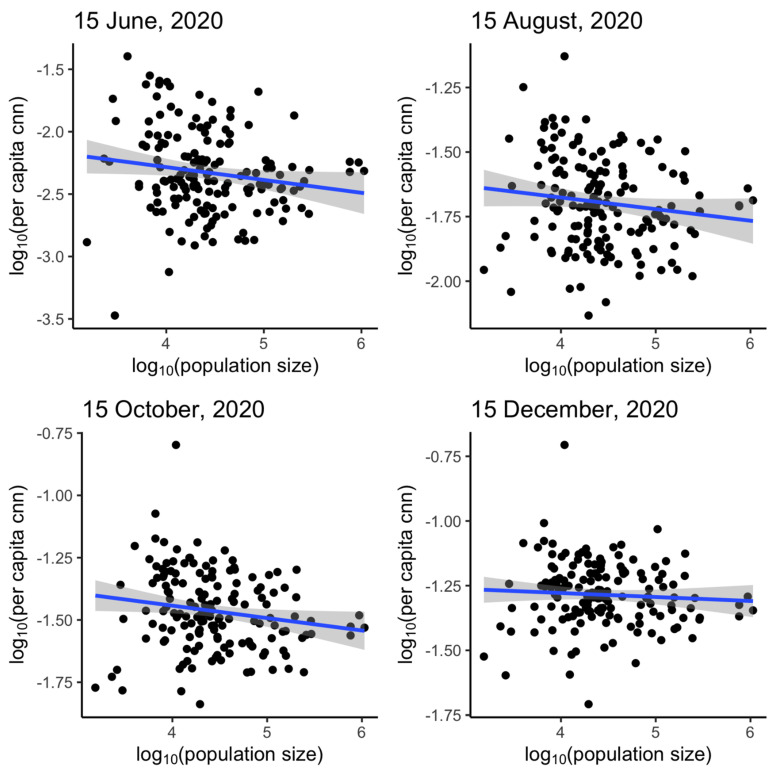
Power-law relationship as described by linear regression between log_10_-transformed per capita cumulative number of new cases (per capita cnn) and log_10_-transformed population size, by county in the state of Georgia.

Figure 5 shows a linear regression plot between the log_10_-transformed per capita cumulative case count and the log_10_-transformed population size for the 18 public health districts of Georgia. Each panel in Figure 5 corresponds to an assessed date, 15 June, 15 August, 15 October, and 15 December 2020, respectively. Table 3 shows that the slopes *m*, for 15 June, 15 August, and 15 October, were negative (statistically significant from 0). This indicates that public health districts with small population size had higher per capita cumulative case count in June, August, and October. However, the slope in December is approximately 0, indicating no heterogeneity of per capita cumulative case count across counties of different population sizes by the end of 2020. This finding echoes our understanding that as the pandemic unfolded, Georgia experienced an intensive community transmission across all counties, and differences in attack rates have diminished over time.

## 4. Discussion

### 4.1. Significance of Our Findings in the Context of Recent Literature

In this study, we estimated the *R_t_* of COVID-19 by public health district in Georgia, USA, from 2 March to 15 December 2020. There was an increase in the cumulative number of COVID-19 cases across various states in the U.S. throughout 2020 [40]. Our findings suggest that Georgia had a widespread COVID-19 outbreak across all health districts within the study period with medium *R_t_* estimates between 1 and 1.4. Our findings are consistent with the findings of Lau and colleagues (2020), who studied 5 Georgia counties over time: Cobb (in health district 3-1), Dekalb (health district 3-5), Fulton (health district 3-2), Gwinnett (in health district 3-4) and Dougherty (in health district 8-2). They showed similar results in the epidemic trajectory (peak in mid-March and subsequent decline to <1 by mid-May) using an entirely different approach [41]. Liu et al. (2020) observed that the estimated medium basic reproductive number *R*_0_ for COVID-19 was 2.79 based on 12 studies regarding *R*_0_ estimates in China between 1 January to 7 February 2020, which was considerably higher than the WHO estimate at 1.95 [42]. Choi et al. (2020) mentioned that in Daegu, North Gyeongsang Province, South Korea, the basic reproductive number was between 3.472 and 3.54 at the peak of the epidemic [43]. Our findings were also similar to that in Kucharski et al. (2020), a mathematical modeling study on the early dynamic of COVID-19 transmission, in which the median reproduction number (*R_t_*) in Wuhan declined from 2.35 (95% CI 1.15–4.77) to 1.05 (0.41–2.39) after travel restriction and shelter-in-place orders were enacted [44]. Along with these studies, our results support the protective benefits of public health measures, such as travel restrictions, physical distancing, and face coverings.

Furthermore, we observed that counties with smaller population sizes tend to have a higher per capita cumulative case count in June, August, and October 2020, but not December 2020 (Table 3). This corresponds to the surge of cases in rural Georgia over the summer after the state government reopened the economy. One recent study observed similar findings and reported that smaller population sizes were associated with higher per capita cumulative case count [45]. Whereas, Politis et al. (2021) reported no significant association between per capita cumulative case count and counties’ population sizes in Arkansas and Kentucky [46]. Future studies should further investigate the effect of population size on the spread of the COVID-19 pandemic.

Regarding the impact of policy change on the COVID-19 transmission, our findings are consistent with previous studies. Studies have reported that policies, such as school closure, shelter-in-place orders, bans on large social gatherings, were associated with a significant decline in the incidence of COVID-19 in the U.S. [47,48,49,50]. In our study, most of the districts observed a similar impact of policies on the COVID-19 transmission. A small difference in the transmission can be attributed to several factors, such as population size, urbanization, and demographic factors. A study by Singh et al. (2021) found that policies were more effective in counties with nonWhite populations, possibly due to a wider spread shutdown of businesses, and in counties with a higher median household income that can be attributed to potential stronger compliance, due to having more flexibility to work remotely in these counties [49].

### 4.2. Limitations

Our study is subject to several limitations. First, the data set acquired from the NYT GitHub did not include demographic information of confirmed COVID-19 cases. Thus, we are unable to do an individual-level analysis of risk factors associated with COVID-19. Meanwhile, during the period of data collection, official guidelines for prevention, diagnosis, and treatment were continually being revised [51]. The uncertainty associated with the quality of the data itself has to be acknowledged. Second, the population size for each district was an estimation created by the U.S. Census Bureau using the 2010 Census dataset. These estimates may or may not be an accurate representation of the actual population size. Third, the surge of new cases could partly be attributed to the availability of nationwide COVID-19 testing rather than an overall increased incidence of COVID-19. However, we believe that the increase in the number of confirmed cases in summer 2020 truly reflected a surge in the number of infections that occurred, due to increased test positivity rates from 5% in early June to 14% in mid-July [19]. Fourth, the dates in the dataset were dates of the case report and not the date of symptom onset. Given a mean incubation period of 6 days and a median delay of 3 days from symptom onset to SARS-CoV-2 testing among SARS-CoV-2 positive patients [52], the estimated *R_t_* reflected the transmission potential of SARS-CoV-2 approximately nine days earlier [37]. Fifth, for the estimation of policy change *R_t_*, for consistency, we choose to apply the same date (August 4; the date after the first school district reopen in-person instruction) to ‘Policy E’, across the state of Georgia and each of its health districts, while we fully understand that the exact date of resuming in-person instructions for the Fall semester (or term) in each school district varied. Nevertheless, we believed that it is a reasonable approach, such that we can compare across each health district and with the state.

## 5. Conclusions

This study documented the extensive community transmission of SARS-CoV-2 across the 18 public health districts in the state of Georgia from 2 March to 15 December 2020, with the median *R_t_* estimates (for both 7-day sliding window and for nonoverlapping window per policy change) between 1 and 1.4 over the time period. The trend of *R_t_* over time was fairly consistent across the 18 health districts. No significant differences were observed regarding the medium *R_t_* estimates across all 18 public health districts by 15 December 2020. We also observed that counties with smaller population sizes tended to have a higher per capita cumulative case count in June, August, and October 2020, but not December 2020. This corresponded to the surge of cases in rural Georgia over the summer after the state government reopened the economy. With an estimated *R_t_* greater than 1, the SARS-CoV-2 continued to be transmitted between individuals through Georgia and all its health districts as of 15 December 2020. The impact of nonpharmaceutical interventions was observed across all health districts in an almost identical manner.

## Figures and Tables

**Table 1:** Community-level, nonpharmaceutical interventions against COVID-19 implemented (and relaxed) by state and local government agencies in Georgia, USA, and its Health Districts 3-2 (Fulton) and 8-2 (South West), from 14 March through 15 December 2020.

	Date	Implemented Measure(s)
**Georgia [20]**		
	14 March 2020	Declaration of public health State of Emergency.
	16 March 2020	School closures to mitigate the spread of COVID-19.
	20 March 2020	Reducing regulations to assist the state’s response to the spread of COVID-19.
	23 March 2020	Executive order requiring individuals with increased risk of complications from COVID-19 to isolate, quarantine, or shelter in place; no gatherings involving ten or more individuals should take place; if there are gatherings, people are required to stand or sit 6 feet apart. The order will expire on 6 April 2020. Expanding temporary licensing of certain medical professions to assist the states’ response to the spread of COVID-19.
	24 March 2020	Reduced regulations to assist the states’ response to the spread of COVID-19.
	2 April 2020	Statewide shelter-in-place executive order. Executive order to limit physical interactions, including shelter-in-place if diagnosed with underlying conditions, closure of bars, and no gatherings involving ten or more individuals should take place.
	8 April 2020	Renewal of public health state of emergency. Renewed for 30 days (until 13 May 2020).
	15 April 2020	Statewide testing for all symptomatic individuals (referral is still needed).
	20 April 2020	Provision of flexibility for healthcare practices, moving certain businesses to minimum operations, and providing for emergency response.
	23 April 2020	Reviving a healthy Georgia. Wearing of face masks, sanitation practices following the guidelines published by the Centers for Disease Control and Prevention, and no gatherings of persons except cohabiting persons.
	27 April 2020	Businesses in the state will start opening following social distancing and prevention measures.
	30 April 2020	The public health state of emergency was issued on 14 March and renewed on 8 April to assist with the state’s COVID-19 response. Shelter in place was mandated for vulnerable individuals.
	12 May 2020	Residents and visitors must practice social distancing and refrain from gathering. Wearing of face coverings. Renewal of public health state of emergency until 30 October 2020.
	5 August 2020	Schools began reopening for both in-person and virtual instruction [21].
**Health district 3-2**		
Fulton County [22]	15 June 2020 17 August 2020	Fulton County reopening measures and service changes. Schools reopen for virtual instruction only [23].
**Health district 8-2**		
Baker County [20]	23 March 2020	Executive order requiring individuals with increased risk of complications from COVID-19 to isolate, quarantine, or shelter in place. No gatherings involving 10 or more individuals should take place, if gatherings require standing or sitting 6 feet apart; order expiring 6 April 2020. Expanding temporary licensing of certain medical professions to assist the states’ response to the spread of COVID-19.
	5 August 2020	Schools began reopening for both in-person and virtual instruction [21].
Calhoun County [24]	11 June 2020	Gatherings of 25 or more persons were banned unless social distancing measures are in place (increased from 10). Bars reopen with strict guidelines. Overnight camps allowed with restrictions. Restrictions for businesses that have been allowed to reopen.
	12 August 2020	Schools reopened the in-person mode of instruction with virtual options [23].
Colquitt County [20]	23 March 2020	Executive order requiring individuals with increased risk of complications from COVID-19 to isolate, quarantine, or shelter in place. No gatherings involving 10 or more individuals should take place, if gatherings require standing or sitting 6 feet apart; order expiring 6 April 2020. Expanding temporary licensing of certain medical professions to assist the states’ response to the spread of COVID-19.
	5 August 2020	Schools began reopening for both in-person and virtual instruction [21].
Decatur County [20]	23 March 2020	Executive order requiring individuals with increased risk of complications from COVID-19 to isolate, quarantine, or shelter in place. No gatherings involving 10 or more individuals should take place, if gatherings require standing or sitting 6feet apart; order expiring 6 April 2020. Expanding temporary licensing of certain medical professions to assist the states’ response to the spread of COVID-19.
	17 August 2020	Schools reopen for virtual instruction only [23].
Dougherty County [25]	25 March 2020	Statewide Shelter-In Place Order. Public or private gatherings. Indoor or outdoor gatherings of 10 persons or less may be permitted while maintaining 6 feet distance. Food serving establishments cease offering dine-in services, may continue offering food to customers via delivery, drive-through, or take-out. Public buildings, parks, and facilities restrictions.
	5 August 2020	Schools began reopening for both in-person and virtual instruction [21].
Early County [26]	16 June 2020	Shelter in place order no longer required for residents and visitors of Georgia who are 65 or older unless they meet certain criteria. Gatherings of more than 50 people were banned unless 6 feet distance is maintained. No longer party maximum for the number of people who can sit together at a restaurant.
	5 August 2020	Schools began reopening for both in-person and virtual instruction [21].
Grady County [27]	28 March 2020	Executive order requiring individuals with increased risk of complications from COVID-19 to isolate, quarantine, or shelter in place. No gatherings involving 10 or more individuals should take place, if gatherings require standing or sitting 6 feet apart; order expiring 6 April 2020. Countywide curfew between 10:00 p.m. until 5:00 a.m.
	5 August 2020	Schools began reopening for both in-person and virtual instruction [21].
Lee County [28]	24 March 2020	Voluntary stay safe at home. Prohibition of gatherings involving more than 10 persons at one time. Recreation and entertainment facilities closed except for facilities where less than 10 persons, including employees, are present at one time. Mandatory curfew between 10:00 p.m. and 6:00 a.m.
	5 August 2020	Schools began reopening for both in-person and virtual instruction [21].
Miller County [20]	23 March 2020	Executive order requiring individuals with increased risk of complications from COVID-19 to isolate, quarantine, or shelter in place. No gatherings involving 10 or more individuals should take place, if gatherings require standing or sitting 6 feet apart; order expiring 6 April 2020. Expanding temporary licensing of certain medical professions to assist the states’ response to the spread of COVID-19.
	5 August 2020	Schools began reopening for both in-person and virtual instruction [21].
Mitchell County [29]	23 March 2020	Prohibition of private or public gatherings of more than 10 individuals. Social distancing measures must be in place for gathering with over 10 individuals. Closure of indoor recreational facilities and business requiring sustained physical contact. Countywide shelter in place for persons with underlying conditions likely to increase the spread of COVID-19.
	5 August 2020	Schools began reopening for both in-person and virtual instruction [21].
Seminole County [30]	25 March 2020	Declaration of state of emergency. Activation of county emergency operations plan and adoption of emergency management ordinances. No gatherings involving 10 or more individuals should take place if gatherings require standing or sitting 6 feet apart.
	5 August 2020	Schools began reopening for both in-person and virtual instruction [21].
Terell County [31]	2 April 2020	Closures of nonessential businesses providing body care not supervised under a licensed medical professional. Closure of indoor and outdoor recreation, fitness, and entertainment facilities. Prohibition of gatherings involving more than 10 persons at one time. Social distancing and sanitation practices.
	5 August 2020	Schools began reopening for both in-person and virtual instruction [21].
Thomas County [20]	23 March 2020	Executive order requiring individuals with increased risk of complications from COVID-19 to isolate, quarantine, or shelter in place. No gatherings involving 10 or more individuals should take place if gatherings require standing or sitting 6 feet apart.
	5 August 2020	Schools began reopening for both in-person and virtual instruction [21].
Worth County [32]	24 March 2020	Executive order requiring individuals with increased risk of complications from COVID-19 to isolate, quarantine, or shelter in place. No gatherings involving 10 or more individuals should take place if gatherings require standing or sitting 6 feet apart; order expiring 6 April 2020.
	5 August 2020	Schools began reopening for both in-person and virtual instruction [21].

**Table 2:** Estimates for the time-varying reproduction number, *R_t_*, on 15 December 2020, for the state of Georgia and its eighteen health districts, using the instantaneous reproduction number method as implemented in the R package ‘EpiEstim’. The analysis used a serial interval following a gamma distribution with a mean of 4.60 days and a standard deviation of 5.55 days, and assumed α = 0.05 a priori.

		15 December 2020 (7-Day Sliding Window)	
Location	First Reported Case (dd-mm-yy)	Median *R_t_* (2.5%, 97.5% Quantiles)	Mean *R_t_* (Standard Deviation)
Georgia	2-Mar-20	1.13 (1.13, 1.14)	1.13 (0.00)
District 1-1 (Northwest Georgia Health District)	6-Mar-20	1.15 (1.11, 1.19)	1.15 (0.02)
District 1-2 (North Georgia Health District)	8-Mar-20	1.13 (1.09, 1.18)	1.13 (0.02)
District 2 (North Health District)	16-Mar-20	1.17 (1.13, 1.21)	1.17 (0.02)
District 3-1 (Douglas Health District)	7-Mar-20	1.12 (1.11, 1.16)	1.12 (0.02)
District 3-2 (Fulton Board of Health)	2-Mar-20	1.07 (1.06, 1.11)	1.07 (0.02)
District 3-3 (Clayton County Health District)	15-Mar-20	1.11 (1.09, 1.19)	1.12 (0.04)
District 3-4 (East Metro Health District)	7-Mar-20	1.13 (1.12, 1.16)	1.13 (0.02)
District 3-5 (Dekalb Health District)	9-Mar-20	1.08 (1.06, 1.12)	1.08 (0.02)
District 4 (District 4 Health District)	9-Mar-20	1.11 (1.07, 1.14)	1.11 (0.02)
District 5-1 (South Central Health District)	19-Mar-20	1.25 (1.14, 1.37)	1.25 (0.06)
District 5-2 (North Central Health District)	18-Mar-20	1.17 (1.12, 1.23)	1.17 (0.03)
District 6 (East Central Health District)	17-Mar-20	1.12 (1.07, 1.16)	1.12 (0.02)
District 7 (West Central Health District)	20-Mar-20	1.14 (1.08, 1.21)	1.14 (0.04)
District 8-1 (South Health District)	11-Mar-20	1.14 (1.12, 1.21)	1.14 (0.03)
District 8-2 (Southwest Health District)	11-Mar-20	1.12 (1.10, 1.19)	1.12 (0.03)
District 9-1 (Coastal Health District)	19-Mar-20	1.10 (1.08, 1.17)	1.10 (0.03)
District 9-2 (Southeast Health District)	10-Mar-20	1.14 (1.12, 1.21)	1.14 (0.03)
District 10 (Northeast Health District)	15-Mar-20	1.17 (1.12, 1.21)	1.17 (0.02)

**Table 3:** The slope (and 95% Confidence Intervals) of the regression line between per capita cumulative number of new cases and population size for the state of Georgia on 7 June, 7 August, 7 October, and 7 December 2020.

	15 June	15 August	15 October	15 December
Georgia	−0.1029 (−0.2022, −0.0036)	−0.0450 (−0.0973, 0.0073)	−0.0498 (−0.0953, −0.0043)	−0.0154 (−0.0525, 0.0216)

## Data Availability

The dataset used in this paper is freely available at NYT Github data repository [33].

## Supplementary Materials

The following are available online at https://www.mdpi.com/article/10.3390/epidemiologia2020014/s1, Online Supplementary Materials. Figure S1: The Daily number of new cases (upper panel) in District 1-1, Georgia, USA, 2 March–15 December 2020, and *R_t_* estimated using the instantaneous reproduction number method implemented in ‘EpiEstim’ package (middle panel: 1-week sliding window; lower panel: policy change *R_t_*), Figure S2: The Daily number of new cases (upper panel) in District 1-2, Georgia, USA, 2 March–15 December 2020, and *R_t_* estimated using the instantaneous reproduction number method implemented in ‘EpiEstim’ package (middle panel: 1-week sliding window; lower panel: policy change *R_t_*), Figure S3: The Daily number of new cases (upper panel) in District 2, Georgia, USA, 2 March–15 December 2020, and *R_t_* estimated using the instantaneous reproduction number method implemented in ‘EpiEstim’ package (middle panel: 1-week sliding window; lower panel: policy change *R_t_*), Figure S4: The Daily number of new cases (upper panel) in District 3-1, Georgia, USA, 2 March–15 December 2020, and *R_t_* estimated using the instantaneous reproduction number method implemented in ‘EpiEstim’ package (middle panel: 1-week sliding window; lower panel: policy change *R_t_*), Figure S5: The Daily number of new cases (upper panel) in District 3-2, Georgia, USA, 2 March–15 December 2020, and *R_t_* estimated using the instantaneous reproduction number method implemented in ‘EpiEstim’ package (middle panel: 1-week sliding window; lower panel: policy change *R_t_*), Figure S6: The Daily number of new cases (upper panel) in District 3-3, Georgia, USA, 2 March–15 December 2020, and *R_t_* estimated using the instantaneous reproduction number method implemented in ‘EpiEstim’ package (middle panel: 1-week sliding window; lower panel: policy change *R_t_*), Figure S7: The Daily number of new cases (upper panel) in District 3-4, Georgia, USA, 2 March–15 December 2020, and *R_t_* estimated using the instantaneous reproduction number method implemented in ‘EpiEstim’ package (middle panel: 1-week sliding window; lower panel: policy change *R_t_*), Figure S8: The Daily number of new cases (upper panel) in District 3-5, Georgia, USA, 2 March–15 December 2020, and *R_t_* estimated using the instantaneous reproduction number method implemented in ‘EpiEstim’ package (middle panel: 1-week sliding window; lower panel: policy change *R_t_*), Figure S9: The Daily number of new cases (upper panel) in District 4, Georgia, USA, 2 March–15 December 2020, and *R_t_* estimated using the instantaneous reproduction number method implemented in ‘EpiEstim’ package (middle panel: 1-week sliding window; lower panel: policy change *R_t_*), Figure S10: The Daily number of new cases (upper panel) in District 5-1, Georgia, USA, 2 March–15 December 2020, and *R_t_* estimated using the instantaneous reproduction number method implemented in ‘EpiEstim’ package (middle panel: 1-week sliding window; lower panel: policy change *R_t_*), Figure S11: The Daily number of new cases (upper panel) in District 5-2, Georgia, USA, 2 March–15 December 2020, and *R_t_* estimated using the instantaneous reproduction number method implemented in ‘EpiEstim’ package (middle panel: 1-week sliding window; lower panel: policy change *R_t_*), Figure S12: The Daily number of new cases (upper panel) in District 6, Georgia, USA, 2 March–15 December 2020, and *R_t_* estimated using the instantaneous reproduction number method implemented in ‘EpiEstim’ package (middle panel: 1-week sliding window; lower panel: policy change *R_t_*), Figure S13: The Daily number of new cases (upper panel) in District 7, Georgia, USA, 2 March–15 December 2020, and *R_t_* estimated using the instantaneous reproduction number method implemented in ‘EpiEstim’ package (middle panel: 1-week sliding window; lower panel: policy change *R_t_*), Figure S14: The Daily number of new cases (upper panel) in District 8-1, Georgia, USA, 2 March–15 December 2020, and *R_t_* estimated using the instantaneous reproduction number method implemented in ‘EpiEstim’ package (middle panel: 1-week sliding window; lower panel: policy change *R_t_*), Figure S15: The Daily number of new cases (upper panel) in District 8-2, Georgia, USA, 2 March–15 December 2020, and *R_t_* estimated using the instantaneous reproduction number method implemented in ‘EpiEstim’ package (middle panel: 1-week sliding window; lower panel: policy change *R_t_*), Figure S16: The Daily number of new cases (upper panel) in District 9-1, Georgia, USA, 2 March–15 December 2020, and *R_t_* estimated using the instantaneous reproduction number method implemented in ‘EpiEstim’ package (middle panel: 1-week sliding window; lower panel: policy change *R_t_*), Figure S17: The Daily number of new cases (upper panel) in District 9-2, Georgia, USA, 2 March–15 December 2020, and *R_t_* estimated using the instantaneous reproduction number method implemented in ‘EpiEstim’ package (middle panel: 1-week sliding window; lower panel: policy change *R_t_*), Figure S18: The Daily number of new cases (upper panel) in District 10, Georgia, USA, 2 March–15 December 2020, and *R_t_* estimated using the instantaneous reproduction number method implemented in ‘EpiEstim’ package (middle panel: 1-week sliding window; lower panel: policy change *R_t_*), Table S1: Control measures implemented by state and local government agencies in eighteen districts of Georgia, USA. Table S2: Time-varying reproduction number in the state of Georgia estimated using nonoverlapping time windows (median and 95% credible interval) and its change between each time window (median and 95% credible interval), Table S3: Time-varying reproduction number in District 1-1 estimated using nonoverlapping time windows (median and 95% credible interval) and its change between each time window (median and 95% credible interval), Table S4: Time-varying reproduction number in District 1-2 estimated using nonoverlapping time windows (median and 95% credible interval) and its change between each time window (median and 95% credible interval), Table S5: Time-varying reproduction number in District 2 estimated using nonoverlapping time windows (median and 95% credible interval) and its change between each time window (median and 95% credible interval), Table S6: Time-varying reproduction number in District 3-1 estimated using nonoverlapping time windows (median and 95% credible interval) and its change between each time window (median and 95% credible interval), Table S7: Time-varying reproduction number in District 3-2 estimated using nonoverlapping time windows (median and 95% credible interval) and its change between each time window (median and 95% credible interval), Table S8: Time-varying reproduction number in District 3-3 estimated using nonoverlapping time windows (median and 95% credible interval) and its change between each time window (median and 95% credible interval), Table S9: Time-varying reproduction number in District 3-4 estimated using nonoverlapping time windows (median and 95% credible interval) and its change between each time window (median and 95% credible interval), Table S10: Time-varying reproduction number in District 3-5 estimated using nonoverlapping time windows (median and 95% credible interval) and its change between each time window (median and 95% credible interval), Table S11: Time-varying reproduction number in District 4 estimated using nonoverlapping time windows (median and 95% credible interval) and its change between each time window (median and 95% credible interval), Table S12: Time-varying reproduction number in District 5-1 estimated using nonoverlapping time windows (median and 95% credible interval) and its change between each time window (median and 95% credible interval), Table S13: Time-varying reproduction number in District 5-2 estimated using nonoverlapping time windows (median and 95% credible interval) and its change between each time window (median and 95% credible interval), Table S14: Time-varying reproduction number in District 6 estimated using nonoverlapping time windows (median and 95% credible interval) and its change between each time window (median and 95% credible interval), Table S15: Time-varying reproduction number in District 7 estimated using nonoverlapping time windows (median and 95% credible interval) and its change between each time window (median and 95% credible interval), Table S16: Time-varying reproduction number in District 8-1 estimated using nonoverlapping time windows (median and 95% credible interval) and its change between each time window (median and 95% credible interval), Table S17: Time-varying reproduction number in District 8-2 estimated using nonoverlapping time windows (median and 95% credible interval) and its change between each time window (median and 95% credible interval), Table S18: Time-varying reproduction number in District 9-1 estimated using nonoverlapping time windows (median and 95% credible interval) and its change between each time window (median and 95% credible interval), Table S19: Time-varying reproduction number in District 9-2 estimated using nonoverlapping time windows (median and 95% credible interval) and its change between each time window (median and 95% credible interval), Table S20: Time-varying reproduction number in District 10 estimated using nonoverlapping time windows (median and 95% credible interval) and its change between each time window (median and 95% credible interval).

## Appendix A. Handling of Negative Incident Case Count Data

If there were negative incident case counts in the data (i.e., when public health agencies made corrections to their cumulative case count data), that was how the data was handled. For all 18 health districts, author CAO and author AKM handled negative incident case counts by correcting the incidence on prior days such that the cumulative case count of the prior days would not exceed the cumulative case count of the day that reported negative incident case counts.

## Appendix B. The Instantaneous Reproduction Number *R_t_* (‘EpiEstim’ Package)

The time-varying reproduction number is denoted as *R_t_* in this paper. Its implementation in the R package ‘EpiEstim’ as described in Cori et al. [36] is also known as the instantaneous reproduction number. *R_t_* was calculated as the ratio between *I_t_*, the number of incident cases at the time *t*, to the total infectiousness of all persons with COVID-19 at the time *t*. Based on Cori et al. [36], we summarized the method as follows.

Λ_*t*_ is mathematically interpreted as:

(A1) $$\Lambda_{t}=\sum_{s=1}^{t}I_{t-s}w_{s}$$

that is, the sum of the occurrence of infection up to *t* − 1, weighted by the infectivity function *w_s_*, a probability distribution that describes the average infection and is typically expressed by the serial interval distribution. Hence, incident cases at time *t* are Poisson-distributed with a mean of

(A2) $$R_{t}\sum_{s=1}^{t}I_{t-s}w_{s}.$$

Contingent on the number of incident cases in previous time points, *I*_0_, …, *I_t_*_−1_, and given the reproduction number *R_t_*, the likelihood of the incidence *I_t_* is as follows:

(A3) $$P\left(I_{t}|I_{0},\ldots ,I_{t-1},w,R_{t}\right)=\frac{{\left(R_{t}\Lambda_{t}\right)}^{I_{t}}\left(e^{-R_{t}\Lambda_{t}}\right)}{I_{t}!}$$

where the total infectiousness of individuals with COVID-19 at time *t* [36],

(A4) $$\Lambda_{t}=\sum_{s=1}^{t}I_{t-s}w_{s}.^{\;}$$

Thus,

(A5) $$R_{t}=\frac{I_{t}}{\sum_{s=1}^{t}I_{t-s}w_{s}}$$

can be interpreted as the average number of secondary cases infected at time *t* by an infectious person if conditions remain the same at the time *t*.

Since this formulation can result in a highly variable *R_t_* estimate over time, the method of instantaneous reproduction number as implemented in ‘EpiEstim’ assumes that the *R_t_* is constant over a given time frame of size *τ* ending at time *t*. This will enable a precise estimate to be estimated with limited variability. Given that transmissibility is assumed to be constant over a period of time, from (*t* − *τ* + 1) to *t*, and is denoted by a reproduction number, *R_t,τ_*, the possibility of the incidence during the time period, from *I*_(*t*−*τ*+1)_ to *I_t_*, is contingent on incidence prior to the time period, i.e., from *I*_0_, to *I*_(*t*−*τ*)_ [36]. For the purpose of this study, we used a 1-week sliding window.

Using a Bayesian framework with a Gamma-distributed prior to *R_t,τ_*, Cori et al. [36] derived an analytical expression of the posterior distribution of *R_t_*, and thus, estimated its median, the variance, and the 95% credible interval [36].

In this paper, *R_t_* analysis was conducted using ‘EpiEstim’ version 2.2-3 [36].
