# Supplementary material for: Spatially Refined Time-Varying Reproduction Numbers of COVID-19 by Health District in Georgia, USA, March–December 2020
Source: Epidemiologia (Basel). 2021 May 28;2(2):179–97. doi: 10.3390/epidemiologia2020014 (PMC9620885; doi:10.3390/epidemiologia2020014)
Supplement: Supplementary file 1 [file epidemiologia-02-00014-s001.zip › epidemiologia-1037287-supplementary.pdf]

Supplementary Materials: Spatially Refined Time-Varying Reproduction Numbers of COVID-19 by Health District in Georgia, USA, March–December 2020

Chigozie A. Ogwara, Arshpreet Kaur Mallhi, Xinyi Hua, Kamalich Muniz-Rodriguez, Jessica S. Schwind, Xiaolu Zhou, Jeffery A. Jones, Joanne Chopak-Foss, Gerardo Chowell, Isaac Chun-Hai Fung

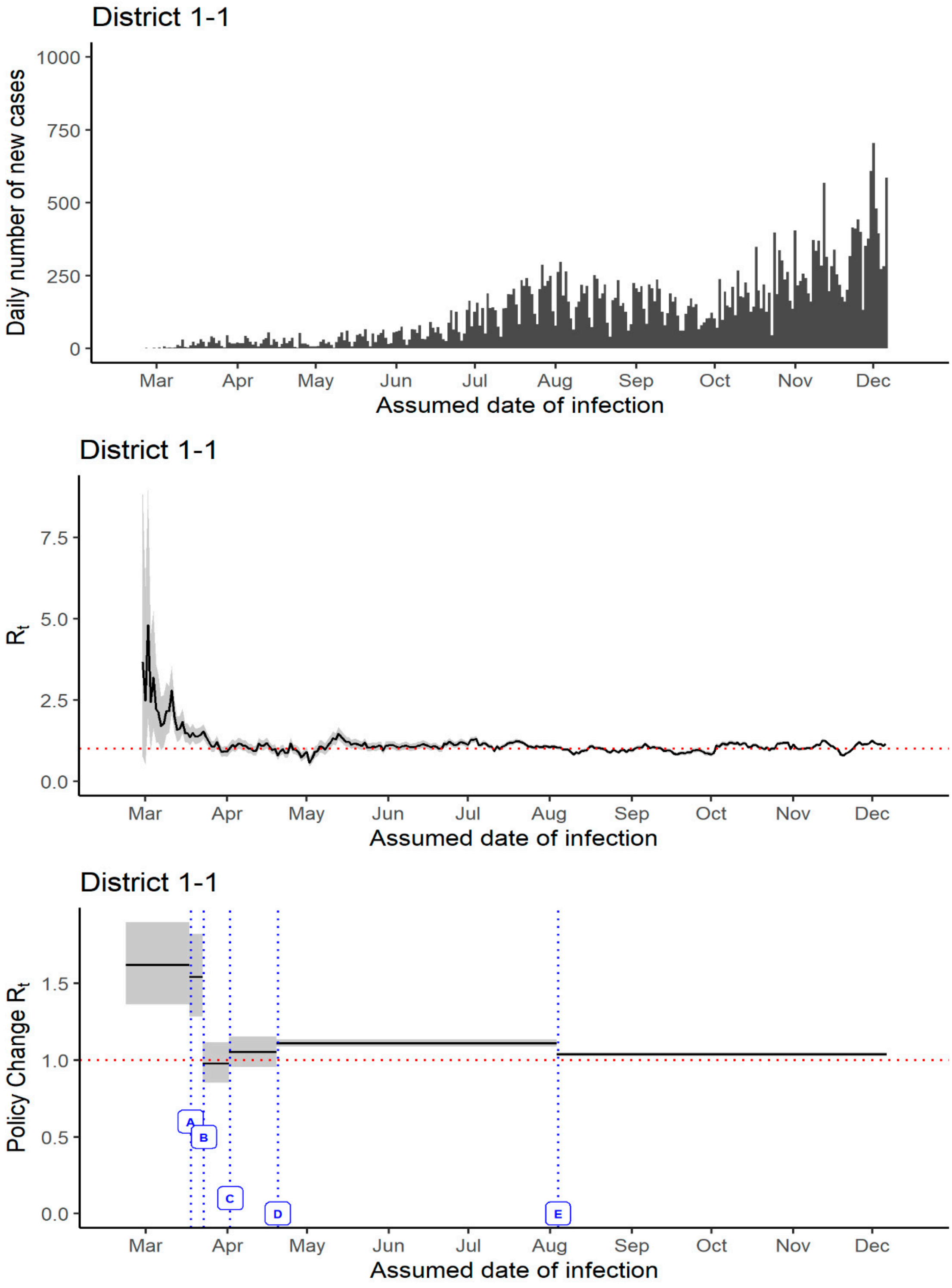

Figure S1. The daily number of new cases (upper panel) in District 1-1, Georgia, USA, 2 March–15 December 2020, and R_t estimated using the instantaneous reproduction number method implemented in 'EpiEstim' package (middle panel: 1-week sliding window; lower panel: policy change R_t).

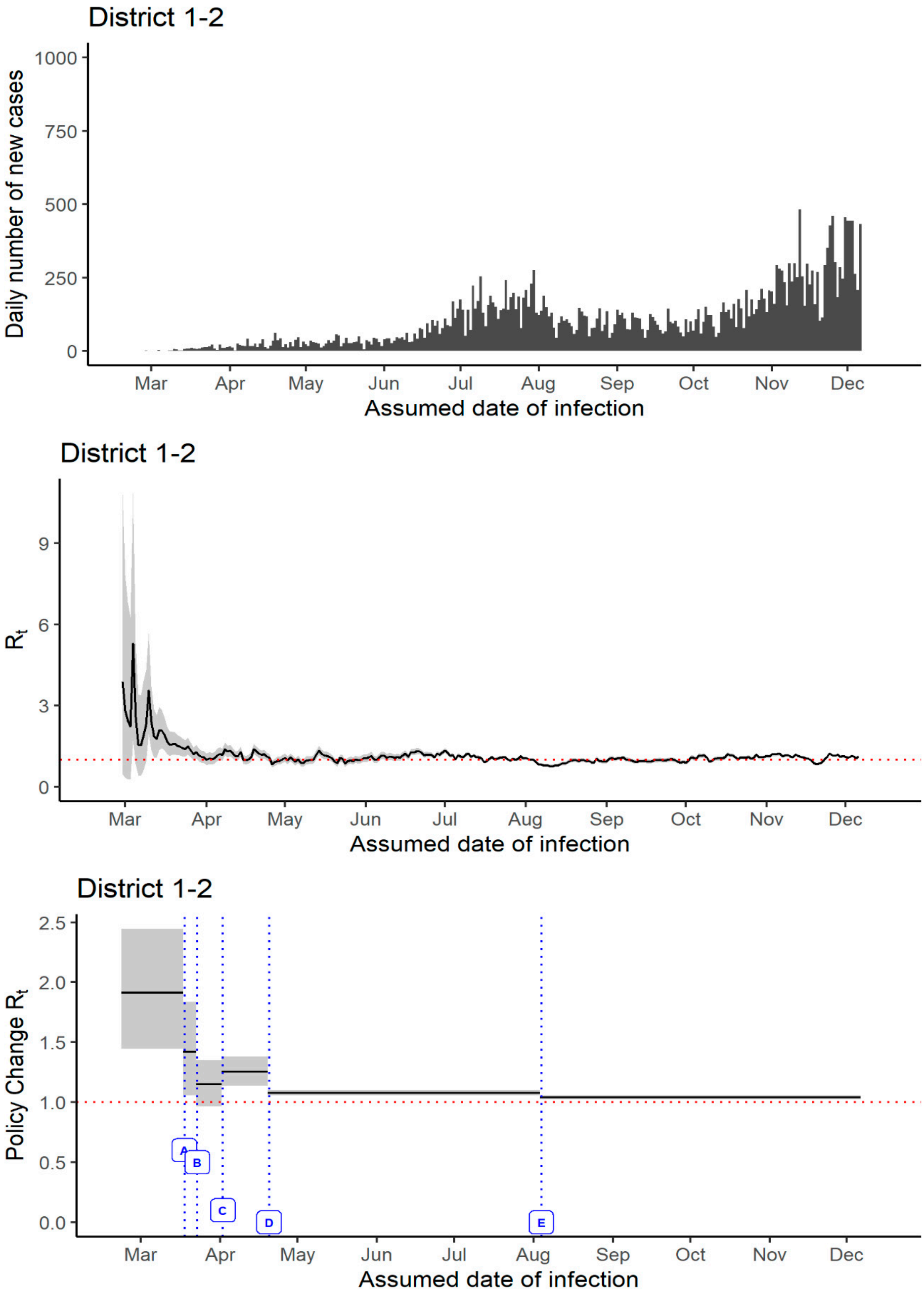

Figure S2. The daily number of new cases (upper panel) in District 1-2, Georgia, USA, 2 March–15 December 2020, and R_t estimated using the instantaneous reproduction number method implemented in 'EpiEstim' package (middle panel: 1-week sliding window; lower panel: policy change R_t).

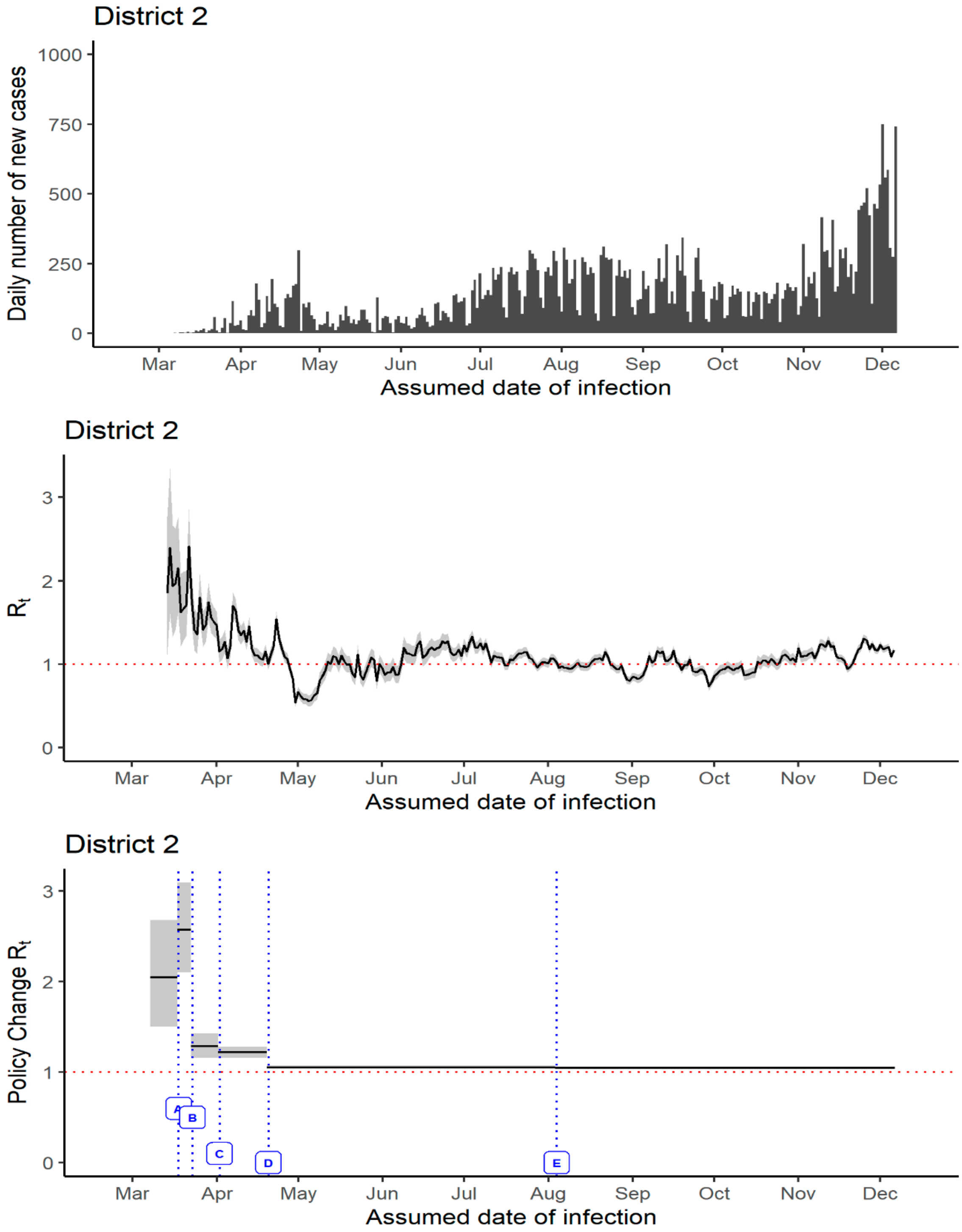

Figure S3. The daily number of new cases (upper panel) in District 2, Georgia, USA, 2 March–15 December 2020, and R_t estimated using the instantaneous reproduction number method implemented in 'EpiEstim' package (middle panel: 1-week sliding window; lower panel: policy change R_t).

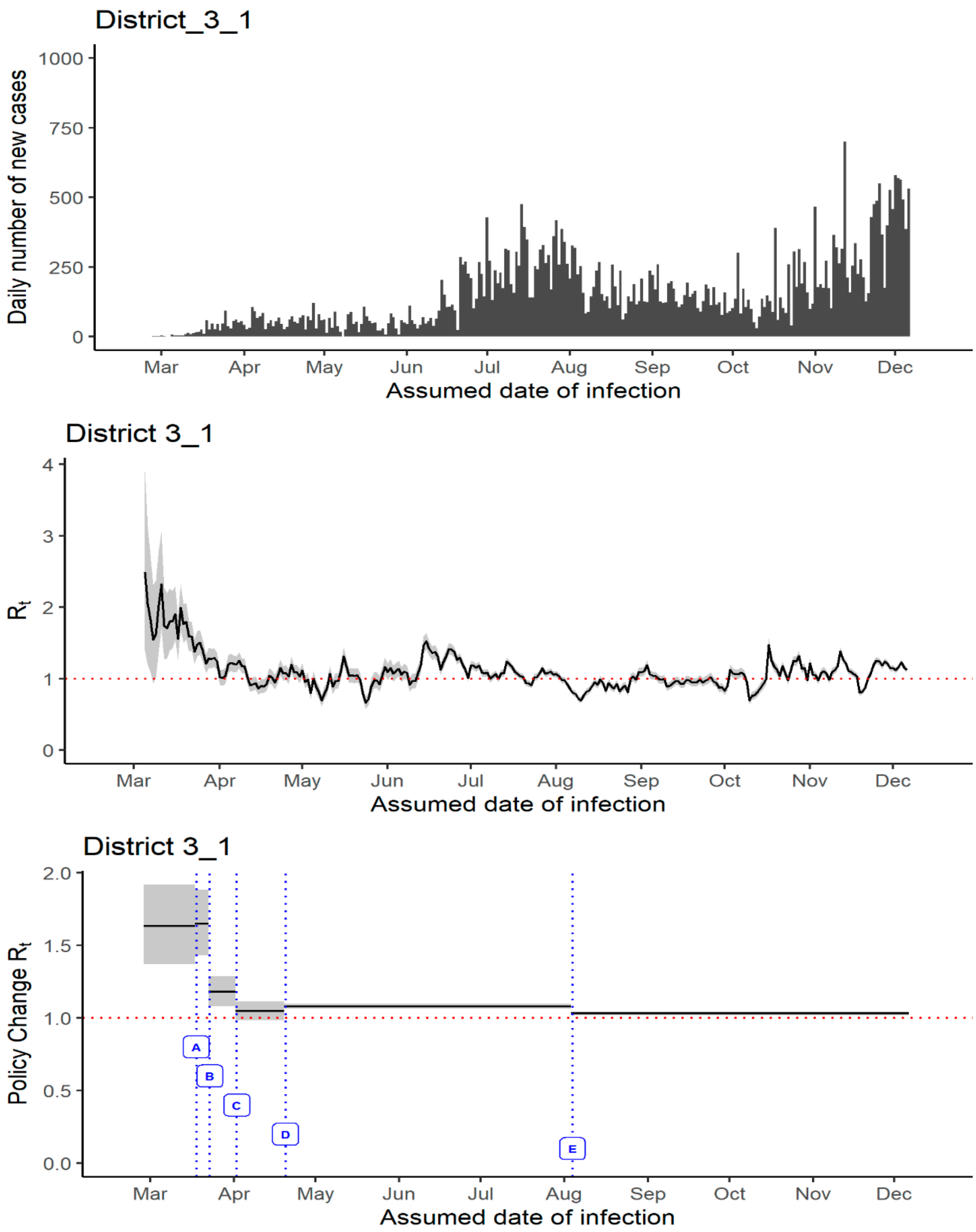

Figure S4. The daily number of new cases (upper panel) in District 3-1, Georgia, USA, 2 March–15 December 2020, and R_t estimated using the instantaneous reproduction number method implemented in 'EpiEstim' package (middle panel: 1-week sliding window; lower panel: policy change R_t).

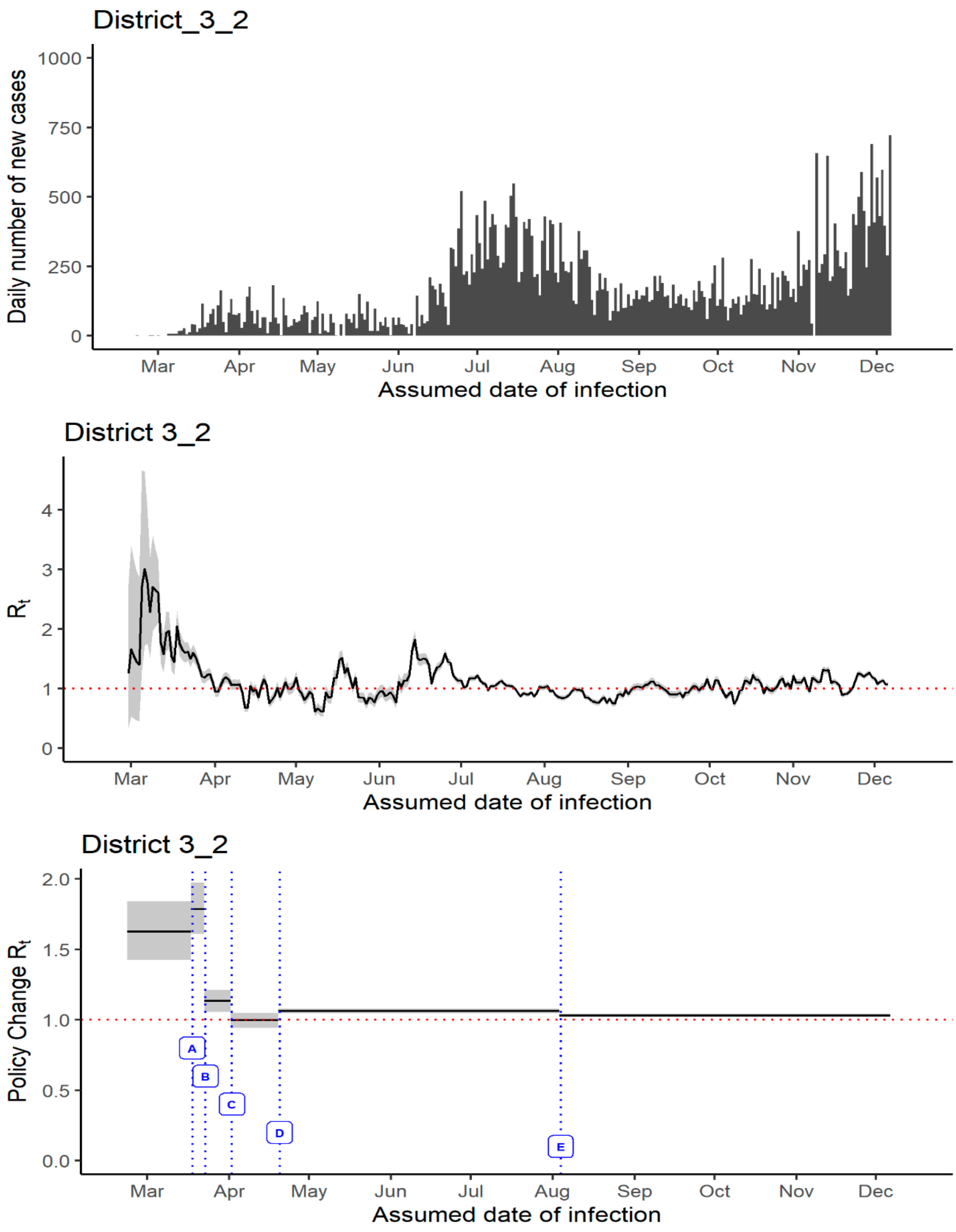

Figure S5. The daily number of new cases (upper panel) in District 3-2, Georgia, USA, 2 March–15 December 2020, and R_t estimated using the instantaneous reproduction number method implemented in 'EpiEstim' package (middle panel: 1-week sliding window; lower panel: policy change R_t).

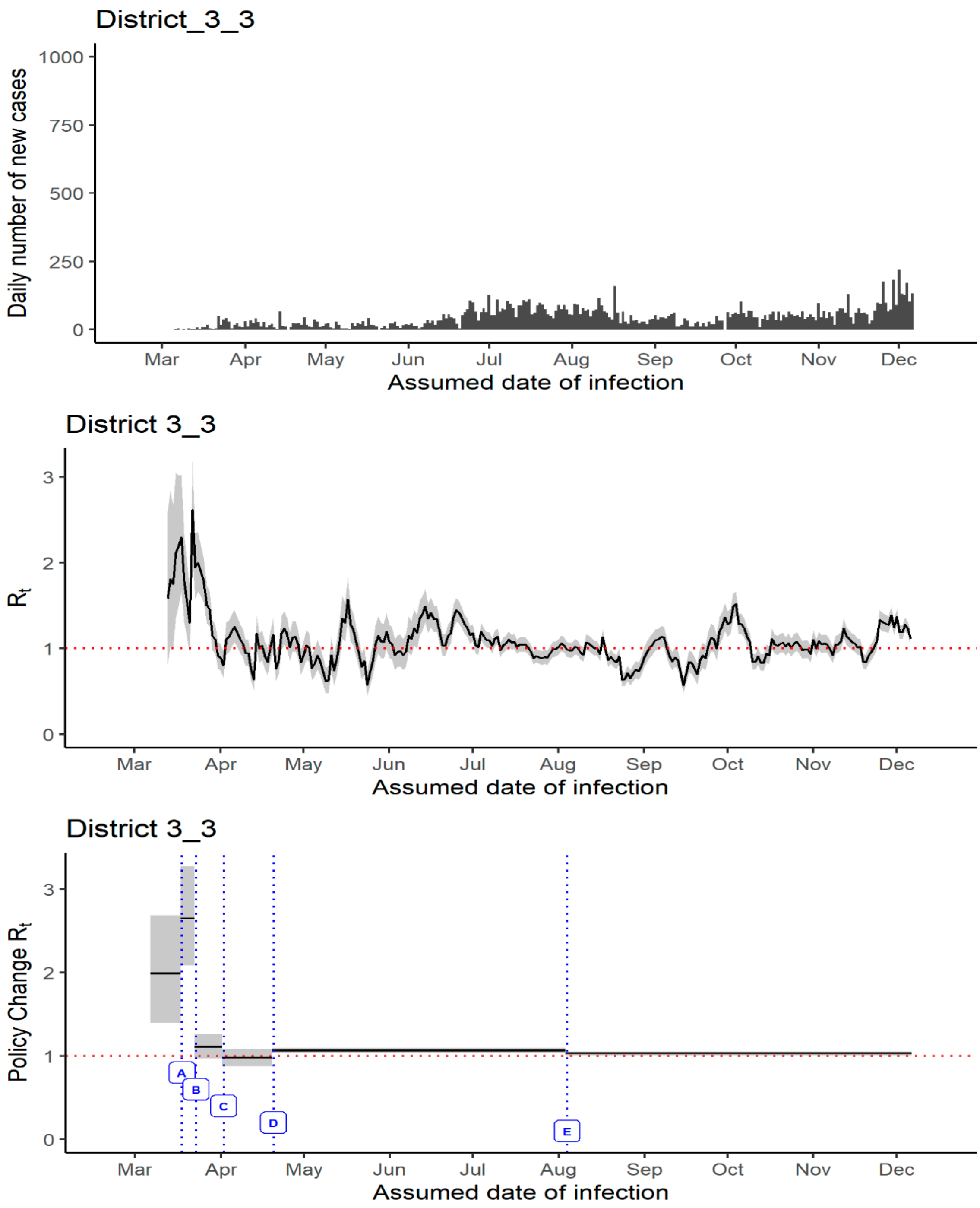

Figure S6. The daily number of new cases (upper panel) in District 3-3, Georgia, USA, 2 March–15 December 2020, and R_t estimated using the instantaneous reproduction number method implemented in 'EpiEstim' package (middle panel: 1-week sliding window; lower panel: policy change R_t).

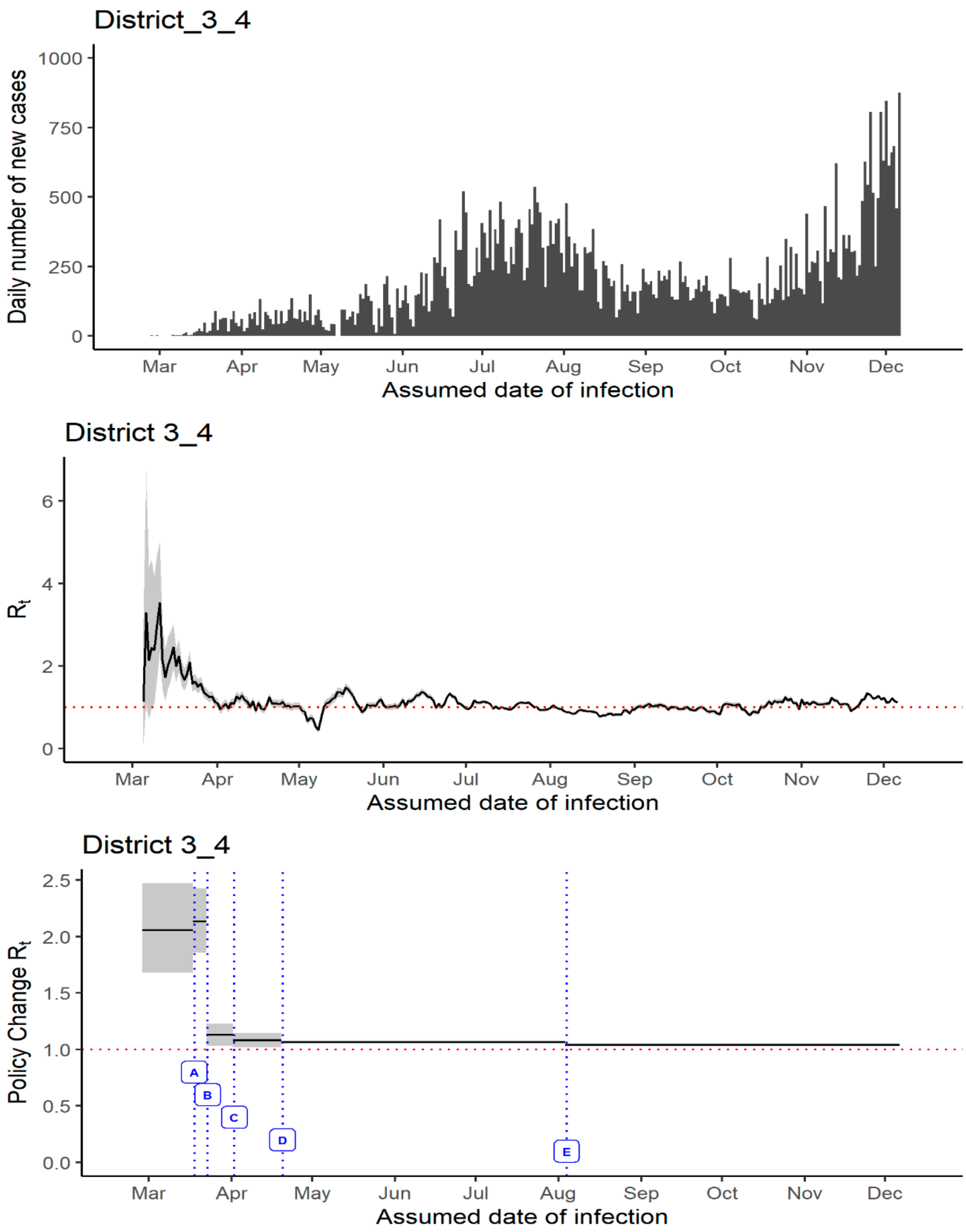

Figure S7. The daily number of new cases (upper panel) in District 3-4, Georgia, USA, 2 March–15 December 2020, and R_t estimated using the instantaneous reproduction number method implemented in 'EpiEstim' package (middle panel: 1-week sliding window; lower panel: policy change R_t).

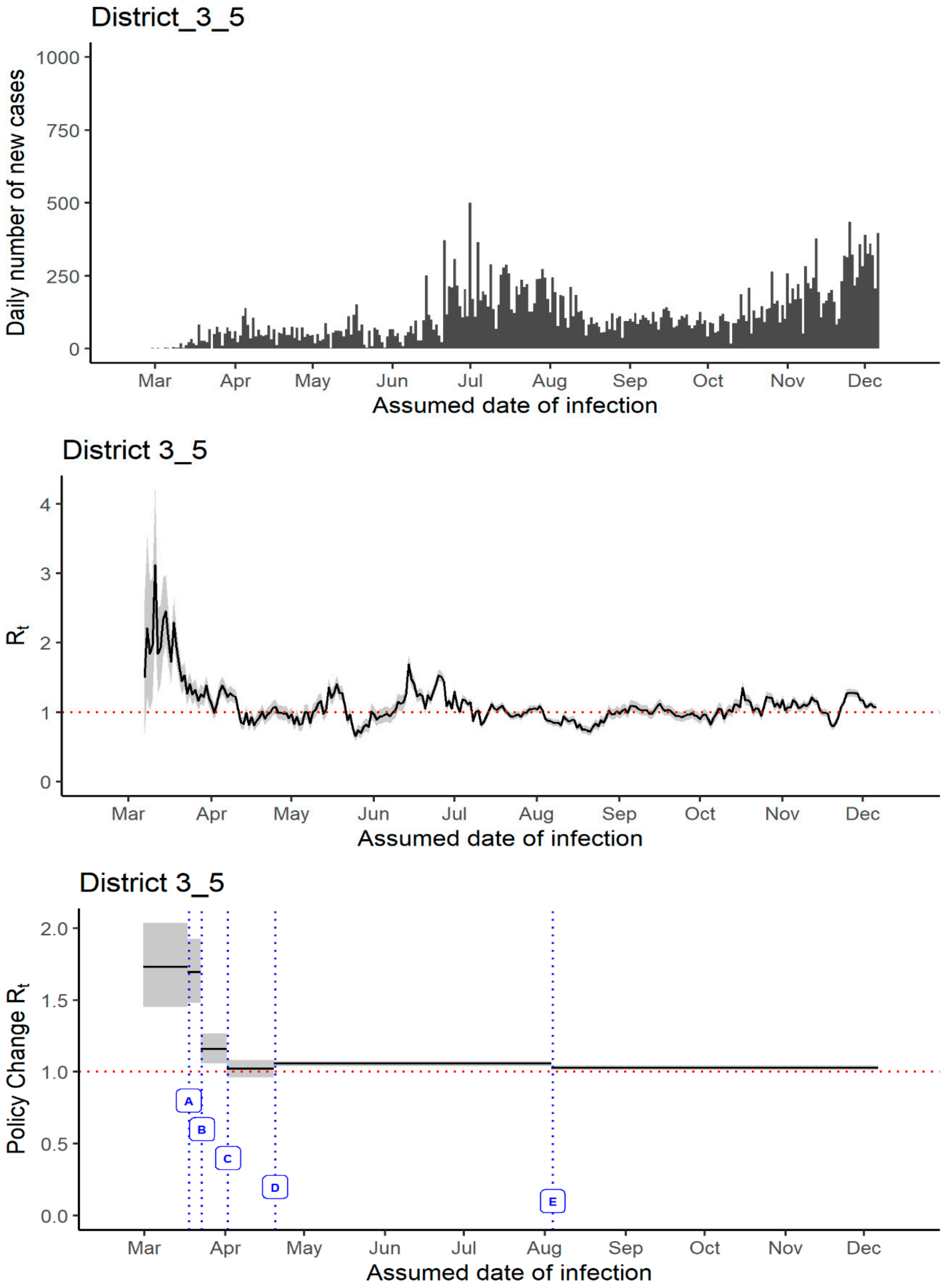

Figure S8. The daily number of new cases (upper panel) in District 3-5, Georgia, USA, 2 March–15 December and R_t estimated using the instantaneous reproduction number method implemented in 'EpiEstim' package (middle panel: 1-week sliding window; lower panel: policy change R_t).

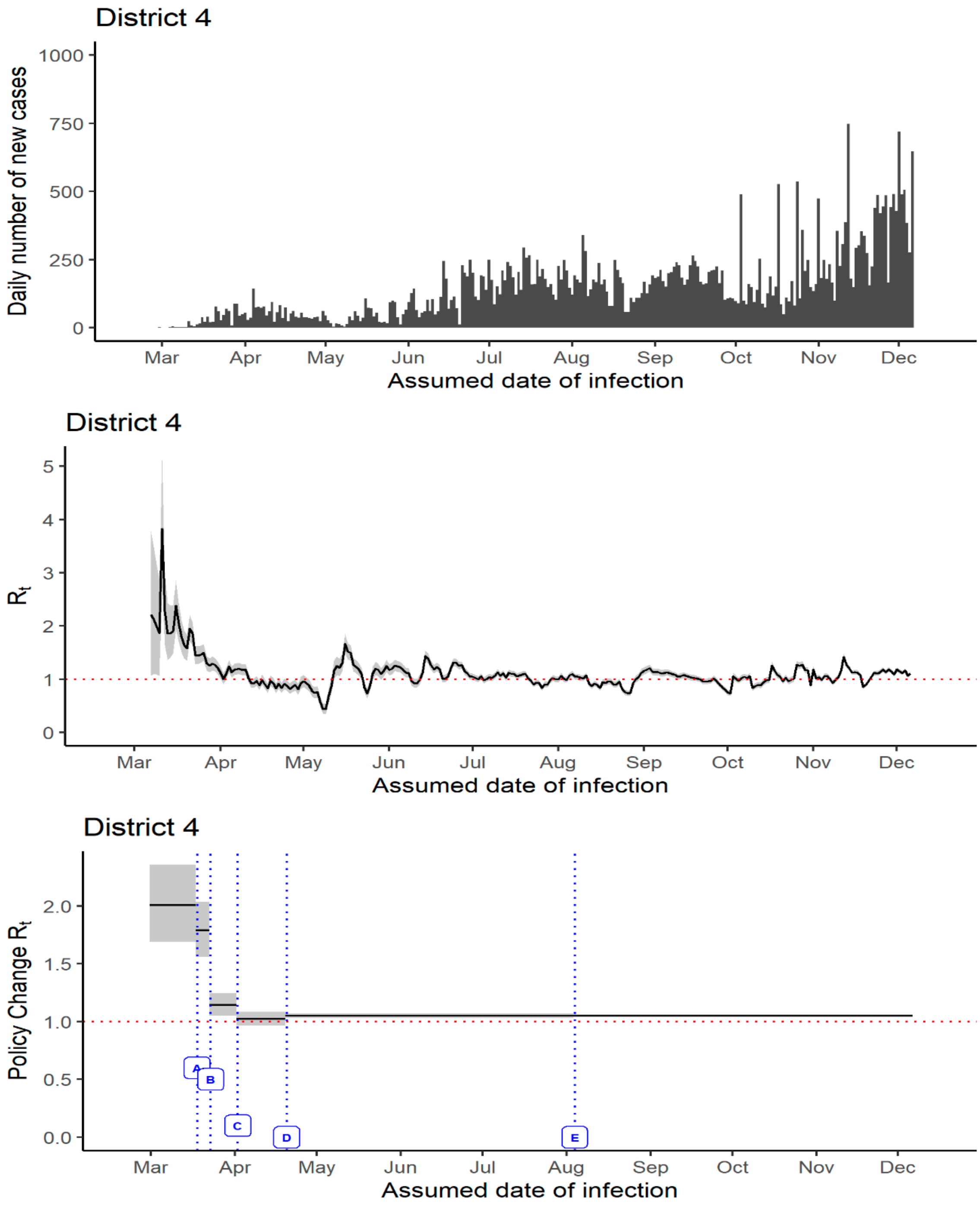

Figure S9. The daily number of new cases (upper panel) in District 4, Georgia, USA, 2 March–15 December and R_t estimated using the instantaneous reproduction number method implemented in 'EpiEstim' package (middle panel: 1-week sliding window; lower panel: policy change R_t).

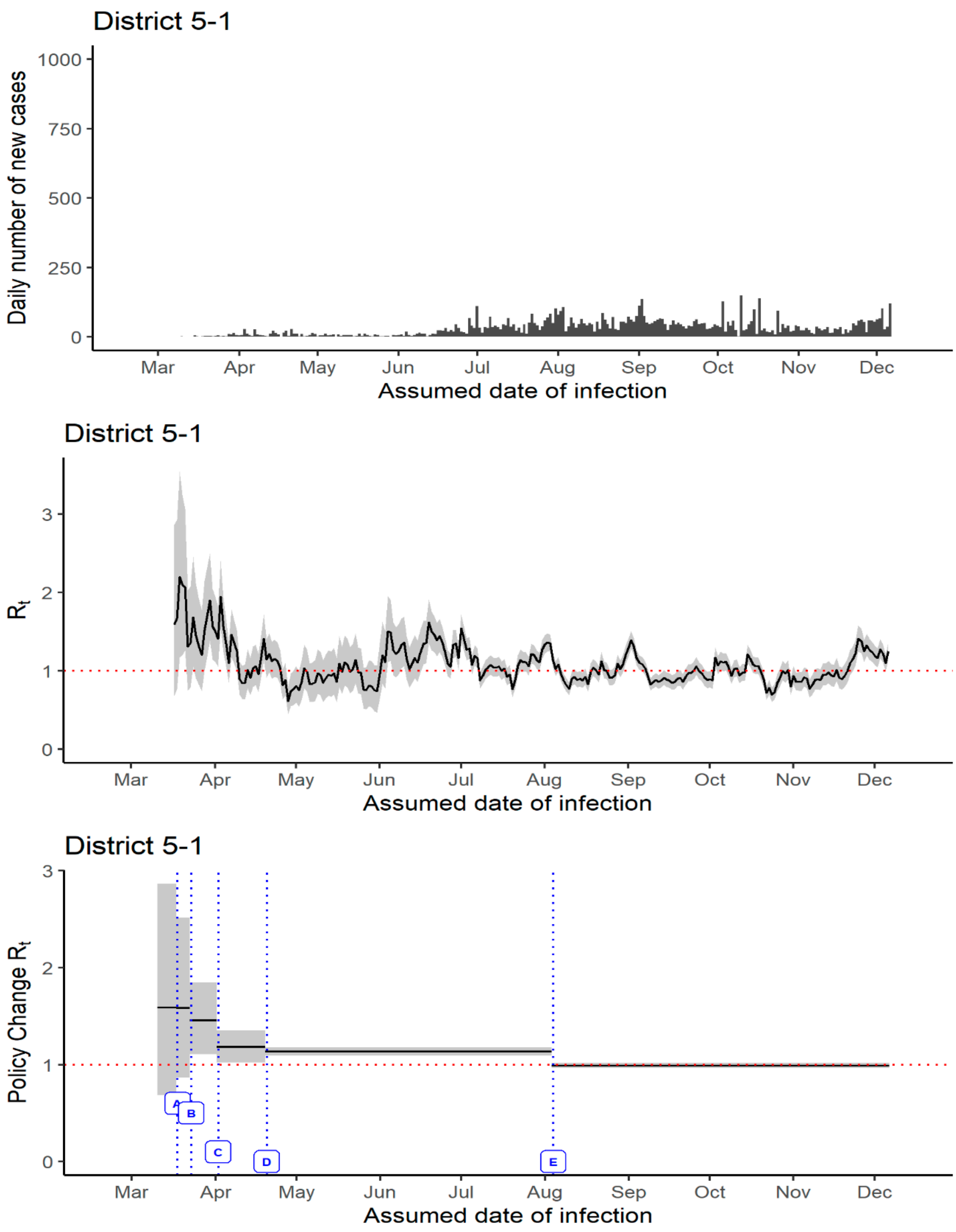

Figure S10. The daily number of new cases (upper panel) in District 5-1, Georgia, USA, 2 March–15 December 2020, and R_t estimated using the instantaneous reproduction number method implemented in 'EpiEstim' package (middle panel: 1-week sliding window; lower panel: policy change R_t).

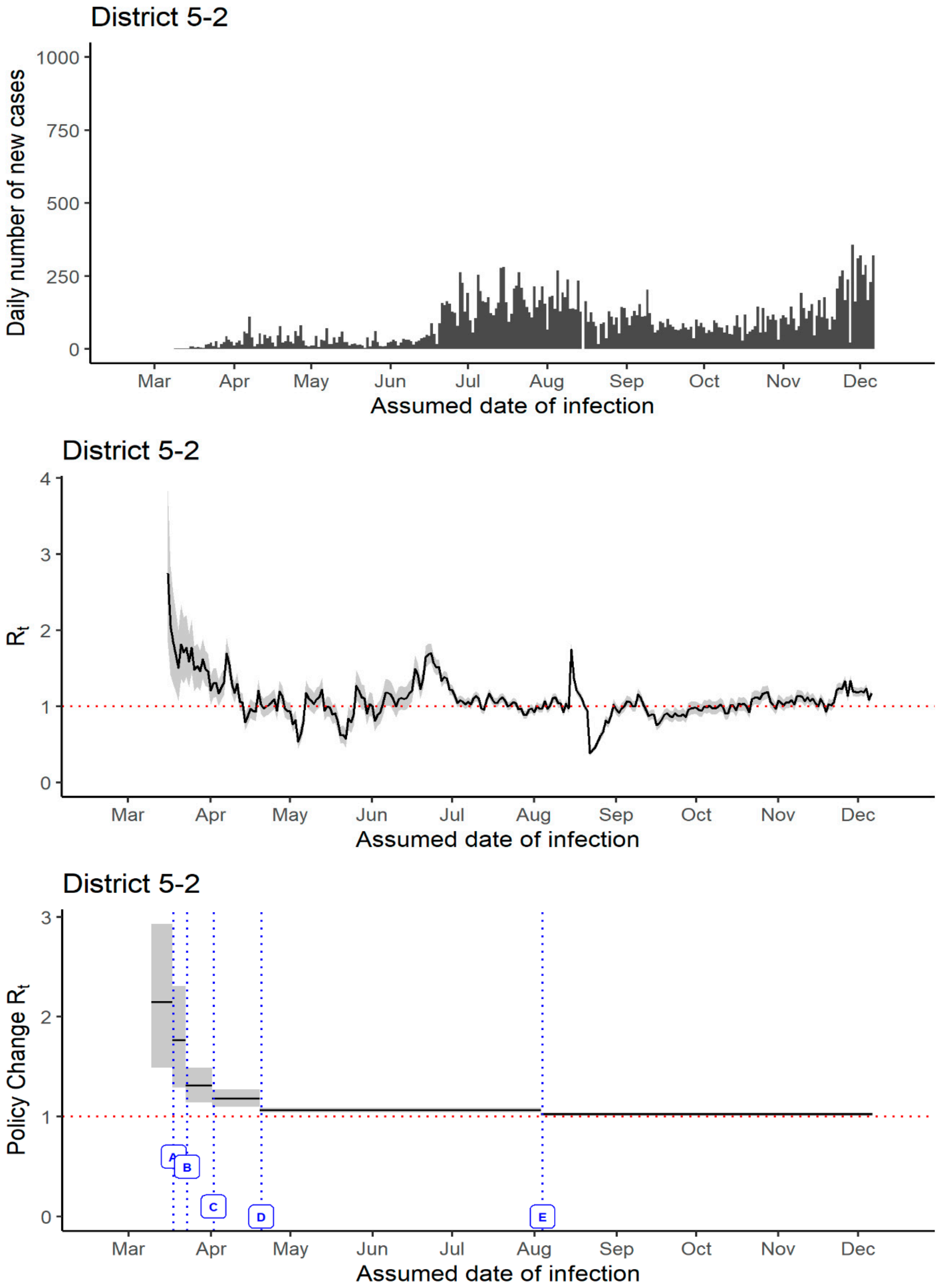

Figure S11. The daily number of new cases (upper panel) in District 5-2, Georgia, USA, 2 March–15 December 2020, and R_t estimated using the instantaneous reproduction number method implemented in 'EpiEstim' package (middle panel: 1-week sliding window; lower panel: policy change R_t).

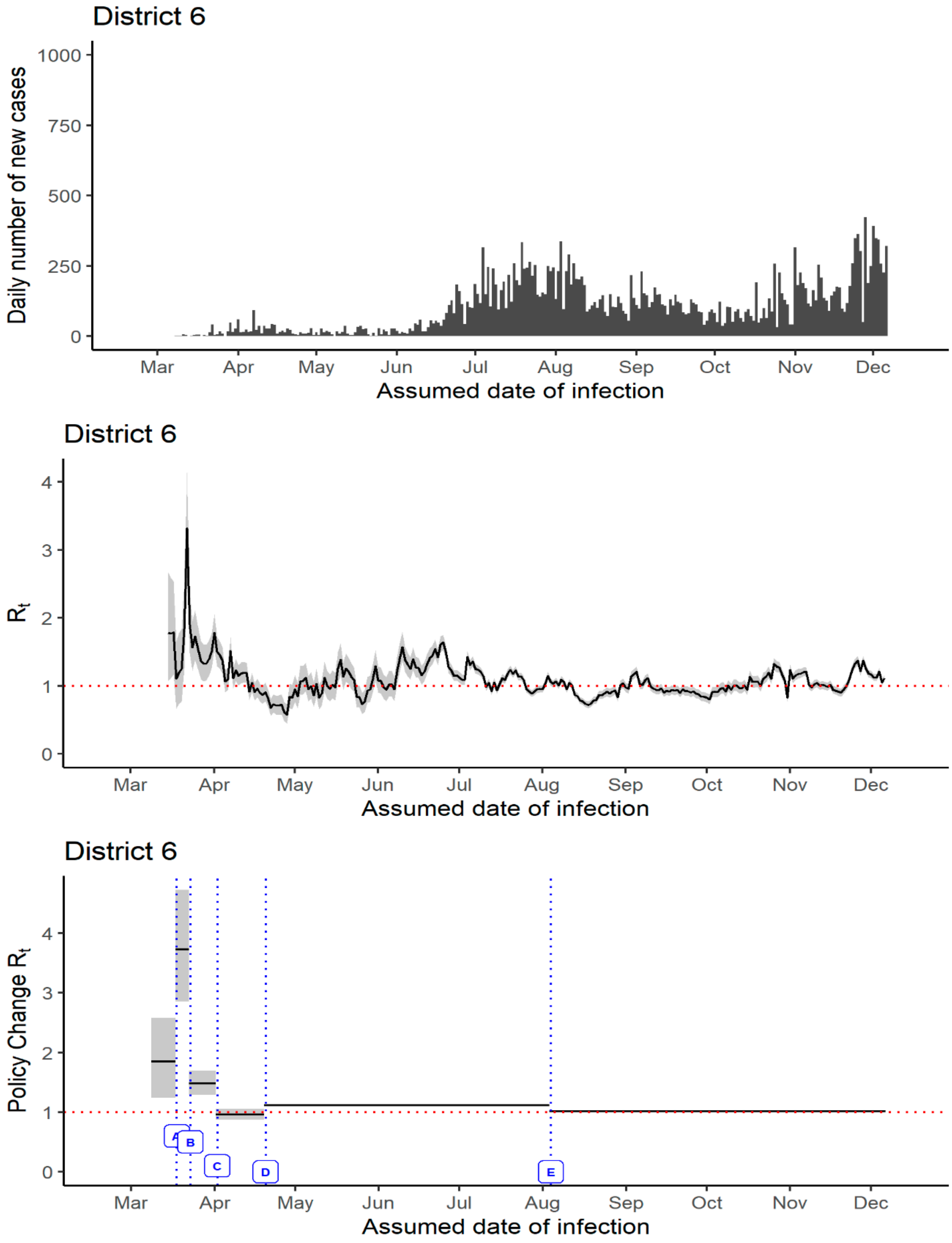

Figure S12. The daily number of new cases (upper panel) in District 6, Georgia, USA, 2 March–15 December 2020, and R_t estimated using the instantaneous reproduction number method implemented in 'EpiEstim' package (middle panel: 1-week sliding window; lower panel: policy change R_t).

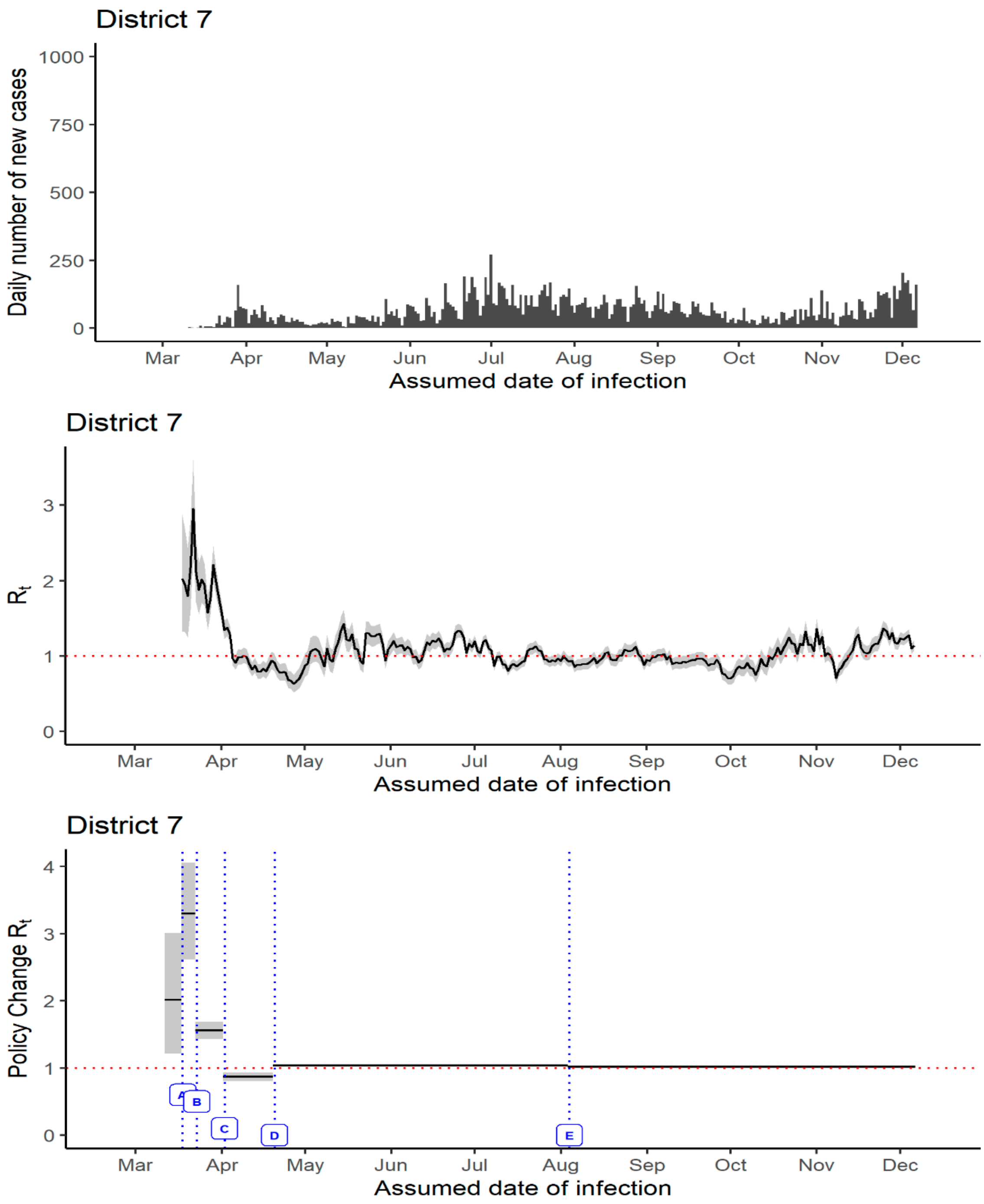

Figure S13. The daily number of new cases (upper panel) in District 7, Georgia, USA, 2 March–15 December 2020, and R_t estimated using the instantaneous reproduction number method implemented in 'EpiEstim' package (middle panel: 1-week sliding window; lower panel: policy change R_t).

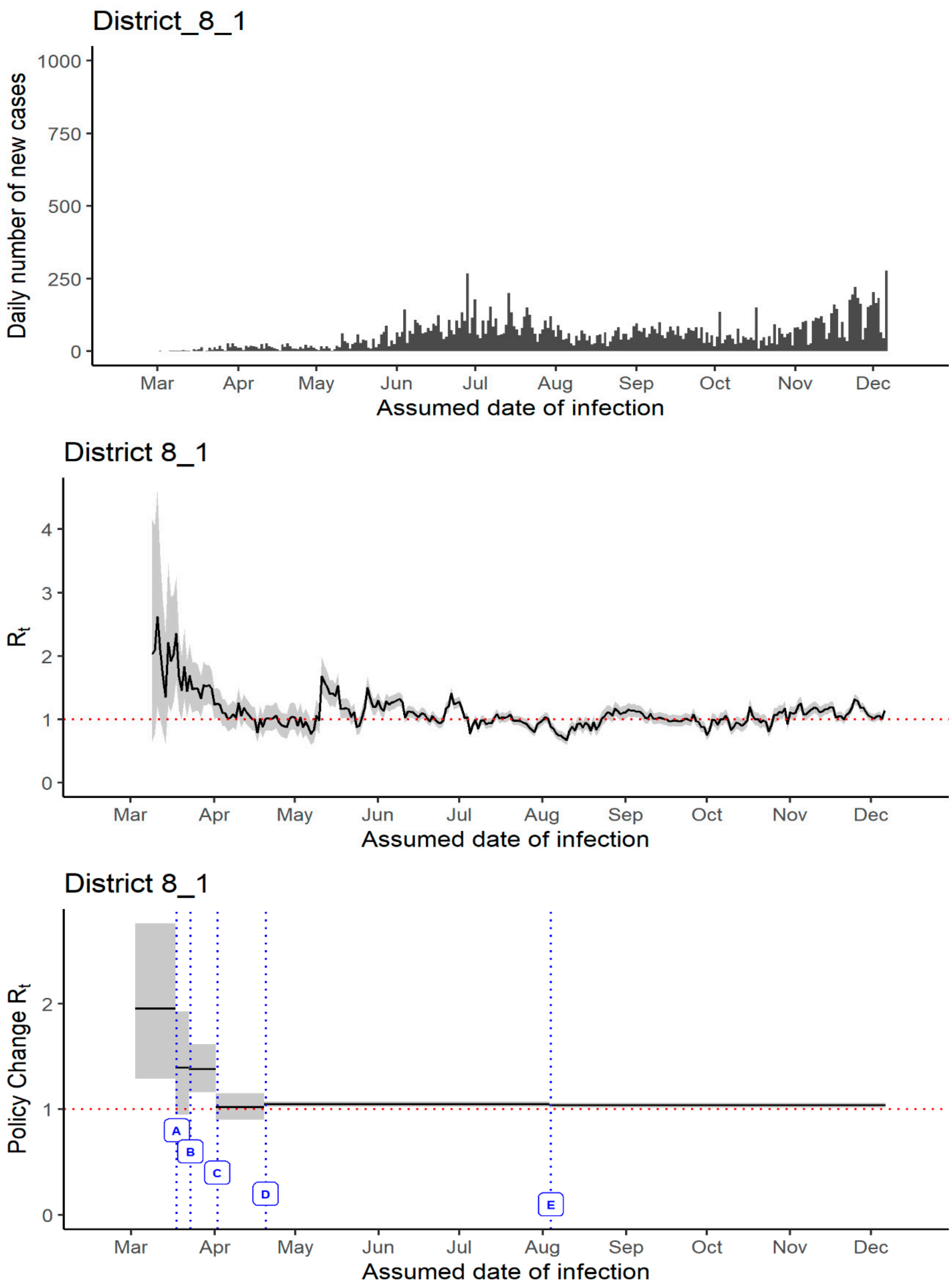

Figure S14. The daily number of new cases (upper panel) in District 8-1, Georgia, USA, 2 March–15 December 2020, and R_t estimated using the instantaneous reproduction number method implemented in 'EpiEstim' package (middle panel: 1-week sliding window; lower panel: policy change R_t).

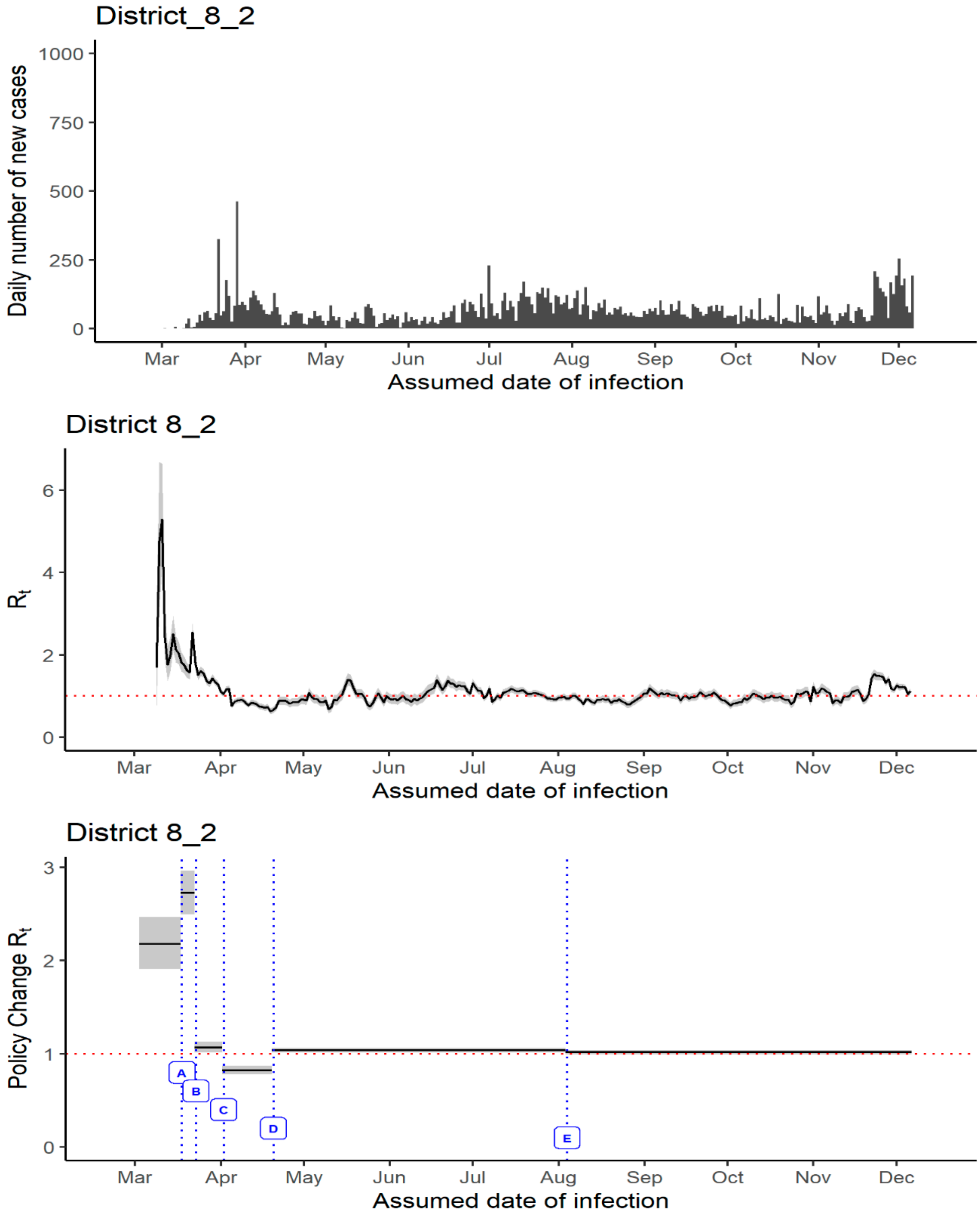

Figure S15. The daily number of new cases (upper panel) in District 8-2, Georgia, USA, 2 March–15 December 2020, and R_t estimated using the instantaneous reproduction number method implemented in 'EpiEstim' package (middle panel: 1-week sliding window; lower panel: policy change R_t).

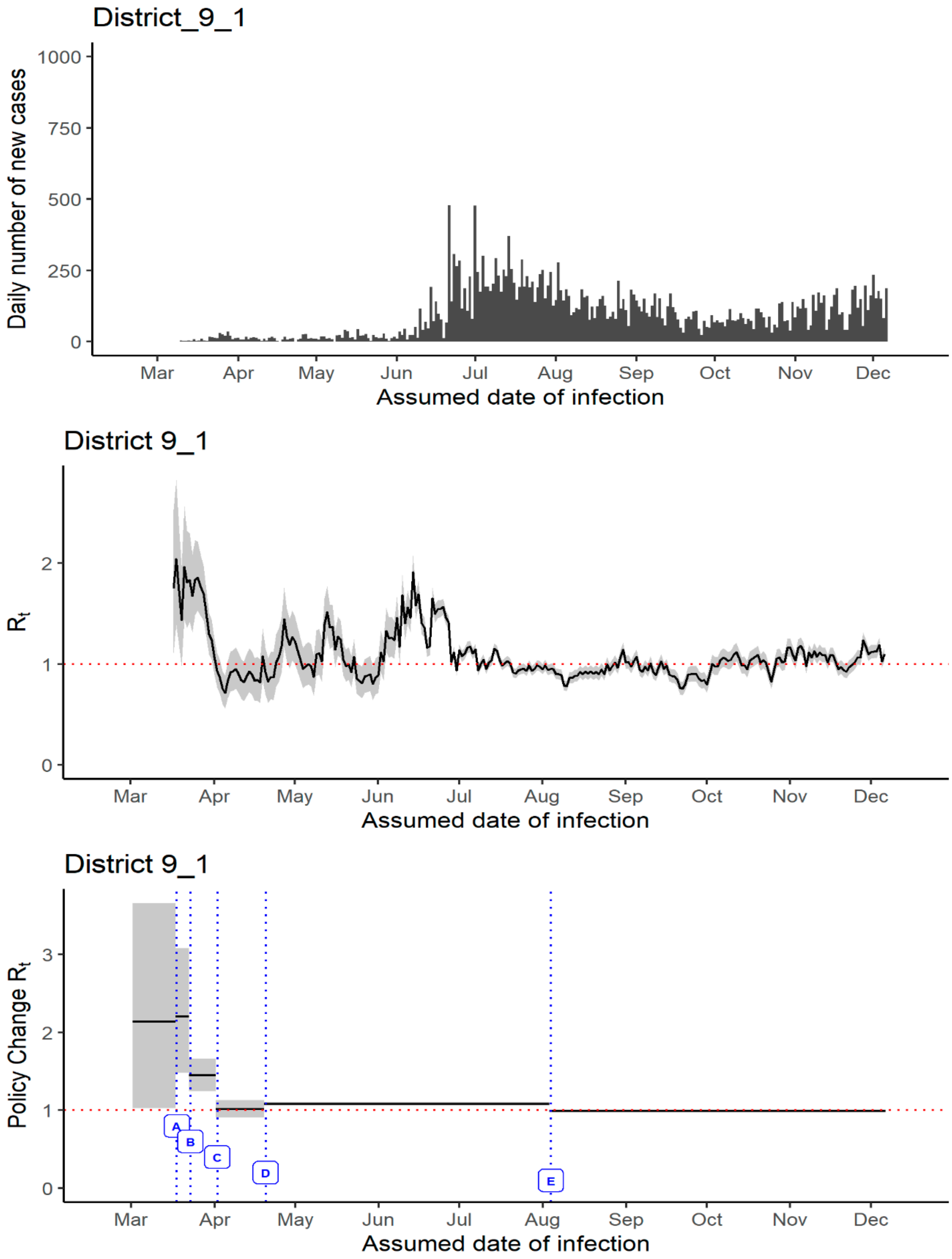

Figure S16. The daily number of new cases (upper panel) in District 9-1, Georgia, USA, 2 March–15 December 2020, and R_t estimated using the instantaneous reproduction number method implemented in 'EpiEstim' package (middle panel: 1-week sliding window; lower panel: policy change R_t).

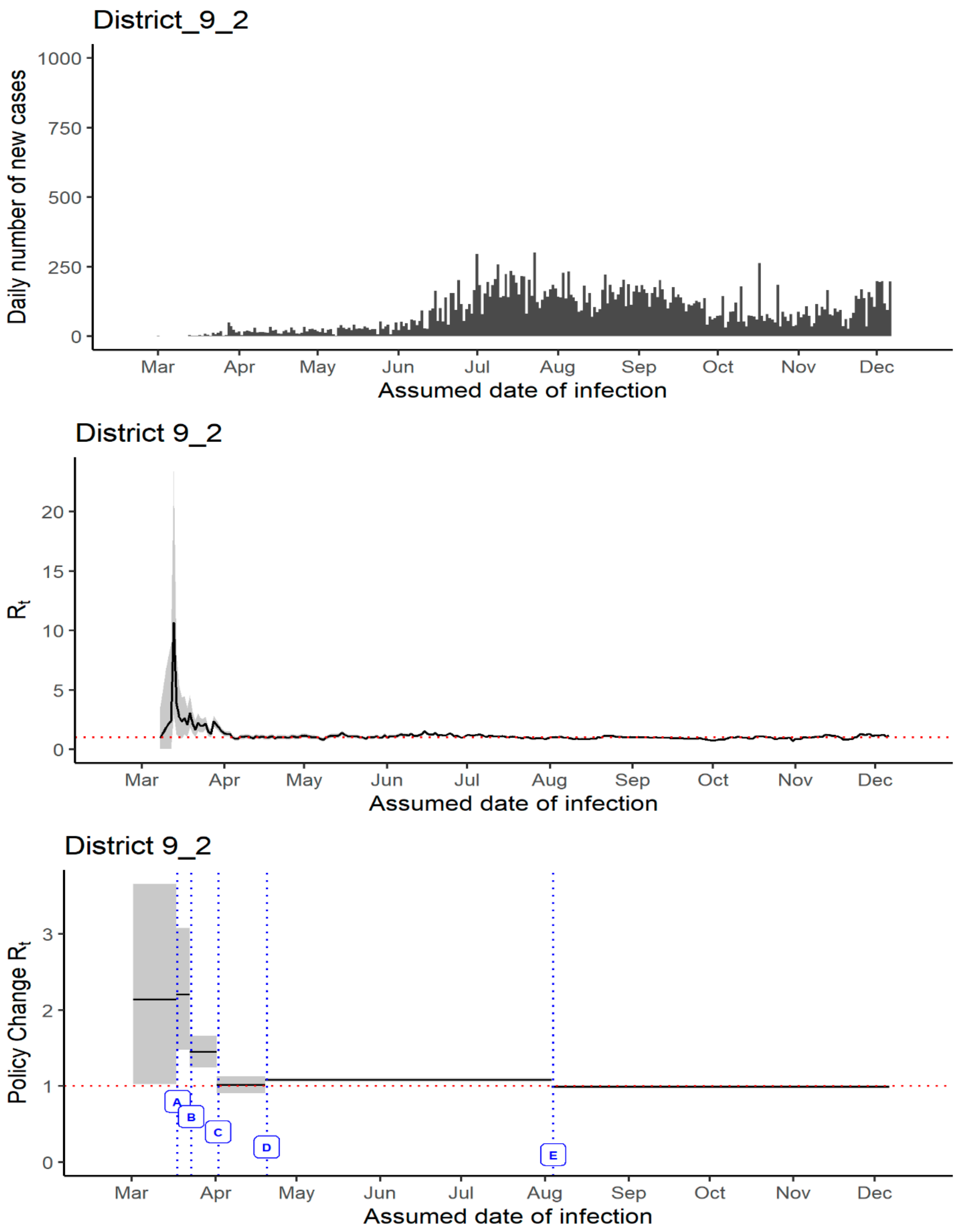

Figure S17. The daily number of new cases (upper panel) in District 9-2, Georgia, USA, 2 March–15 December 2020, and R_t estimated using the instantaneous reproduction number method implemented in 'EpiEstim' package (middle panel: 1-week sliding window; lower panel: policy change R_t).

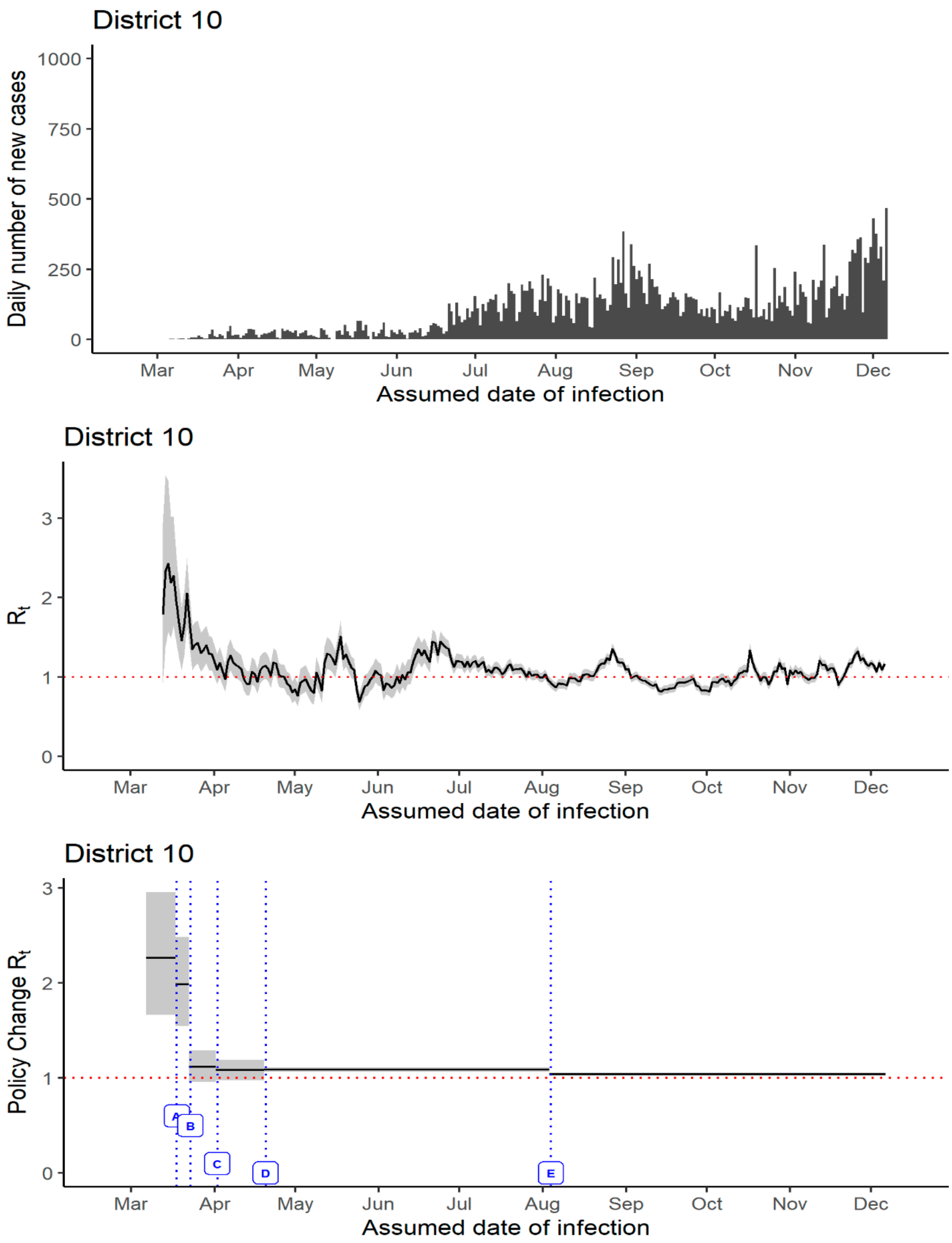

Figure S18. The daily number of new cases (upper panel) in District 10, Georgia, USA, 2 March–15 December 2020, and R_t estimated using the instantaneous reproduction number method implemented in 'EpiEstim' package (middle panel: 1-week sliding window; lower panel: policy change R_t).

Table S1. Control measures implemented by state and local government agencies in eighteen districts of Georgia, USA.

	**Date**	**Implemented measure(s)**
Georgia [1]	14 March 2020	Declaration of public health state of emergency.
	16 March 2020	School closures to mitigate the spread of Covid-19.
	20 March 2020	Reducing Regulations to assist the state's response to the spread of Covid-19

	23 March 2020	Executive order requiring individuals with increased risk of complications from Covid-19 to isolate, quarantine or shelter in place.<br>No gatherings involving 10 or more individuals should take place, if gatherings require standing or sitting 6feet apart; order expiring April 6, 2020.<br>Expanding temporary licensing of certain medical professions to assist the states response to the spread of Covid-19.
	24 March 2020	Reduced regulations to assist the states response to the spread of Covid-19.
	2 April 2020	State-wide shelter-in-place executive order. Executive order to limit physical interactions including shelter-in-place if diagnosed with underlying conditions, closure of bars, and no gatherings involving 10 or more individuals should take place.
	8 April 2020	Renewal of public health state of emergency. Renewed for 30days (until May 13, 2020)
	15 April 2020	State-wide testing for all symptomatic individuals (referral is still needed).
	20 April 2020	Provision of flexibility for healthcare practices, moving certain businesses to minimum operations, and providing for emergency response.
	23 April 2020	Reviving a healthy Georgia. Wearing of face masks, sanitation practices in accordance with the guidelines published by the Centers for Disease Control and Prevention and no gatherings of persons except cohabiting persons
	27 April 2020	Businesses in the state will start opening following social distancing and prevention measures.
	30 April 2020	Public health state of emergency renewed issued on March 14 and renewed April 8 to assist with state's covid-19 response. Shelter in place for vulnerable individuals.
	12 May 2020	Residents and visitors must practice social distancing and refrain from gathering. Wearing of face coverings. Renewal of public health state of emergency until 30 October 2020.
	5 August 2020	Schools began re-opening for both in-person and virtual instruction [2].

Northwest GA Health District 1-1

Bartow County [3]	24 March 2020	State of emergency declared. Indoor and outdoor sitting capacity reduced to 10 persons at one time, additionally, a 6-foot distance should be maintained at all times. All publicly owned active recreational facilities closed until further notice. Order effective until 3 April 2020.
	5 August 2020	Schools opened for in-person instruction with virtual options offered [4].
Catoosa County [5]	16 March 2020	Declaration of precautionary public health emergency, in effect until 15 April 2020.
	24 March 2020	State of emergency declared. Indoor and outdoor sitting capacity reduced to 10 persons at one time, additionally, a 6-foot distance should be maintained at all times. All publicly owned active recreational facilities closed until further notice. Order effective until 3 April 2020.
	24 April 2020	Medical practices and health care institutions should begin patient treatment in accordance with the CDC guidelines. Gyms, fitness centers hair designers may begin in-person minimum basic operations as defined by executive order.

	5 August 2020	Schools began re-opening for both in-person and virtual instruction [2].
Chattooga County [6]	23 March 2020	Executive order requiring individuals with increased risk of complications from Covid-19 to isolate, quarantine or shelter in place.
	12 May 2020	Restaurants, salons, & gyms allowed to open.
	28 May 2020	Bars & nightclubs are allowed to open.
	29 June 2020	Live performance venues are allowed to open.
	30 July 2020	Schools re-open in-person instruction with virtual option [4].
Dade County [7]	25 March 2020	Closures of non-essential businesses providing body care not supervised under a licensed medical professional. Closure of indoor and outdoor recreation, fitness, and entertainment facilities.
	16 June 2020	Gatherings of more than 50 people banned unless there is at least 6ft distance between each person. No party maximum for number of people who can sit at a restaurant.
	5 August 2020	Schools began re-opening for both in-person and virtual instruction [2].
Floyd County [8]	23 March 2020	Executive order requiring individuals with increased risk of complications from Covid-19 to isolate, quarantine or shelter in place.
	7 April 2020	Shelter in place order for all residents and visitors. Social distancing order.
	12 May 2020	Live performance venues, bars, nightclubs, and operators of amusement park rides must remain closed through May 31, 2020.
	13 August 2020	Still undecided [4].
Gordon County [9]	23 March 2020	Executive order requiring individuals with increased risk of complications from Covid-19 to isolate, quarantine or shelter in place.
	7 April 2020	Shelter in place order for all residents and visitors. Social distancing order (6ft part).
	12 August 2020	Schools re-open for virtual instruction only [4].
Haralson County [10]	23 March 2020	Executive order requiring individuals with increased risk of complications from Covid-19 to isolate, quarantine or shelter in place.
	7 April 2020	Shelter in place order for all residents and visitors. Social distancing order.
	13 April 2020	Public health state of emergency and executive order extended.
	4 August 2020	School begins re-opening with virtual option with in-person beginning on 11 August [4].
Paulding County [11]	26 March 2020	County wide state of emergency.

	3 August 2020	Schools begin in-person instruction with virtual options offered [4].
Polk County [12]	30 March 2020	State of emergency until 12 June 2020.
	5 May 2020	Statement concerning wearing of face mask.
	3 August 2020	Schools begin in-person instruction with virtual options offered [4].
Walker County [13]	22 April 2020	Minimum operations executive order.
	13 August 2020	Schools begin in-person instruction with virtual options offered [4].

Dalton North GA Health District 1-2

Cherokee County [14]	14 March 2020	State-wide shelter in place order.
	3 August 2020	Schools re-open for in-person instruction with virtual options offered [4].
Fannin County [15]	4 May 2020	Re-opening of recreational facilities.
	7 August 2020	Schools re-open for in-person instruction with virtual options offered [4].
Gilmer County [16]	23 March 2020	State of emergency declared. Indoor and outdoor sitting capacity reduced to 10 persons at one time, additionally, a 6-foot distance should be maintained at all times. All publicly owned active recreational facilities closed until further notice.
	7 August 2020	Schools re-open for in-person instruction with virtual options offered [4].
Murray County [1]	23 May 2020	Shelter in place order for citizens >65 and those with underlying health conditions.<br>Gatherings of 25 or more persons banned unless social distancing measures are in place (increased from 10).<br>Bars re-open with strict guidelines.<br>Overnight camps allowed with restrictions.<br>Restrictions for businesses that have been allowed to re-open.
	8 August 2020	Schools began re-opening for both in-person and virtual instruction [2].
Pickens County [17]	22 April 2020	Re-opening of businesses for minimum basic operations.
	17 August 2020	Schools begin re-opening with in-person instruction and virtual options offered [4].
Whitfield County [18]	23 March 2020	Indoor and outdoor sitting capacity reduced to 10 persons at one time, additionally, a 6-foot distance should be maintained at all times. All publicly owned active recreational facilities closed until further notice.

Gainesville North GA Health District 2

Banks County [19]	30 March 2020	Social gatherings of 50 or more cancelled for a two-week period. Limit on exposure to crowds for a two-week period.
	19 August 2020	Schools re-opened in person with virtual option [4].

Dawson County [20]	31 March 2020	Executive order requiring individuals with increased risk of complications from Covid-19 to isolate, quarantine or shelter in place.<br>No gatherings involving 10 or more individuals should take place, if gatherings require standing or sitting 6feet apart.
	7 August 2020	Schools re-open for in-person instruction with virtual option [4].
Forsyth County [1]	25 March 2020	State of emergency declared. Indoor and outdoor sitting capacity reduced to 10 persons at one time, additionally, a 6-foot distance should be maintained at all times. All publicly owned active recreational facilities closed until further notice.
	13 August 2020	Schools re-open for in-person instruction with virtual option offered [4].
Franklin County [21]	23 May 2020	Shelter in place order for citizens >65 and those with underlying health conditions.<br>Gatherings of 25 or more persons banned unless social distancing measures are in place (increased from 10).<br>Bars re-open with strict guidelines.<br>Overnight camps allowed with restrictions.<br>Restrictions for businesses that have been allowed to re-open.
	7 August 2020	Schools re-open for in-person instruction with virtual option offered [4].
Habersham County [22]	29 June 2020	Executive order renewing public health of emergency.<br>Executive order empowering a healthy Georgia.
	8 September 2020	Schools re-open for in-person instruction with virtual option offered [4].
Hall County [23]	18 May 2020	State of emergency extended until 12 July 2020.
	24 August 2020	Schools re-open for hybrid instruction [4].
Hart County [24]	23 March 2020	Executive order requiring individuals with increased risk of complications from Covid-19 to isolate, quarantine or shelter in place.<br>No gatherings involving 10 or more individuals should take place, if gatherings require standing or sitting 6feet apart; order expiring 6 April 2020.
	2 April 2020	State-wide shelter-in-place executive order. Executive order to limit physical interactions including shelter-in-place if diagnosed with underlying conditions, closure of bars, and no gatherings involving 10 or more individuals should take place.
	5 August 2020	Schools began re-opening for both in-person and virtual instruction [2].

Lumpkin County [25]	23 March 2020	Voluntary stay safe at home.<br>Prohibition of gatherings involving more than 10 persons at one time.<br>Recreation and entertainment facilities closed except for facilities where less than 10 persons including employees are present at one time.
	10 August 2020	Schools begin in-person instruction with virtual option [4].
Rabun County [1]	23 March 2020	Limited number of persons at one time at retail stores-grocery/non-grocery, pharmaceutical, and building supplies.<br>Close down of all indoor dining room facilities.<br>Countywide curfew between 9:00pm until 6:00am.<br>Gatherings limited to 10 individuals.<br>Cancellation of in-house services at churches.<br>Mandatory social distancing of 6-foot.
	12 August 2020	Schools begin in-person instruction with virtual options offered [4].
Stephens County [26]	24 March 2020	Prohibition of private or public gatherings of more than 10 individuals. Social distancing measures must be in place for gathering with over 10 individuals.<br>Closure of indoor recreational facilities and business requiring sustained physical contact.<br>Countywide shelter in place for persons with underlying conditions likely to increase spread of Covid-19.
	1 May 2020	Shelter in place order lifted for most Georgia residents excluding the elderly and medically fragile.
	5 August 2020	Schools began re-opening for both in-person and virtual instruction [4].
Towns County [27]	24 January 2020	Declaration of state of emergency. Activation of county emergency operations plan and adoption of emergency management ordinances.
	17 August 2020	School re-open for in-person instruction with virtual options offered [4].
Union County [28]	23 March 2020	Closures of non-essential businesses providing body care not supervised under a licensed medical professional.<br>Closure of indoor and outdoor recreation, fitness, and entertainment facilities.<br>Prohibition of gatherings involving more than 10 persons at one time.
	17 August 2020	Schools re-open for in-person instruction with virtual option [4].
White County [29]	23 March 2020	Executive order requiring individuals with increased risk of complications from Covid-19 to isolate, quarantine or shelter in place.<br>No gatherings involving 10 or more individuals should take place if gatherings require standing or sitting 6feet apart.

	14 August 2020	Schools re-open for in-person instruction with virtual option offered [4].

Health districts in Atlanta (3-1, 3-2, 3-3, 3-4, 3-5)

Cobb County [30]	11 June 2020	Gatherings of 25 or more persons banned unless social distancing measures are in place (increased from 10).<br>Bars re-open with strict guidelines.<br>Overnight camps allowed with restrictions.<br>Restrictions for businesses that have been allowed to reopen.
	17 August 2020	Schools re-open for virtual instruction only [4].
Douglas County[31]	25 March 2020	Executive order requiring individuals with increased risk of complications from Covid-19 to isolate, quarantine or shelter in place. Shelter in place order for citizens >65 and those with underlying health conditions.<br>Prohibition of private or public gatherings of more than 10 individuals. Social distancing measures must be in place for gathering with over 10 individuals.
	17 August 2020	Schools re-open for virtual instruction only [4].
Fulton County [32]	15 June 2020	Fulton County re-opening measures and service changes.
	17 August 2020	Schools re-open for virtual instruction only [4].
Clayton County [33]	23 March 2020	Statewide shelter in place order, isolate or quarantine for individuals with increased risk of complications from Covid-19. No gatherings of more than 10 individuals unless gatherings require standing or sitting 6feet apart.
	10 August 2020	Schools re-open for virtual mode of instruction only [4].
Gwinnett County [34]	27 March 2020	Shelter-In Place Order.<br>Public or private gatherings. Indoor or outdoor gatherings of 10 persons or less maybe permitted while maintaining 6feet distance.<br>Food serving establishments cease offering dine-in services, may continue offering food to customers via delivery, drive through or take-out.<br>Public buildings, parks and facilities restrictions.
	12 August 2020	Schools re-open for virtual mode of instruction only [4].

Newton County [35]	31 March 2020	Shelter-In Place Order.<br>Public or private gatherings. Indoor or outdoor gatherings of 10 persons or less maybe permitted while maintaining 6feet distance.<br>Food serving establishments cease offering dine-in services, may continue offering food to customers via delivery, drive through or take-out.<br>Public buildings, parks and facilities restrictions.<br>Executive orders on 'essential travels' only.
	8 September 2020	Schools re-open for virtual instruction only [4].
Rockdale County [36]	3 April 2020	Rockdale County courthouse remains open for essential court business during public health emergency.
	24 August 2020	Schools re-open for virtual instruction only [4].
Dekalb County [37]	23 March 2020	Voluntary stay safe at home.<br>Prohibition of gatherings involving more than 10 persons at one time.<br>Recreation and entertainment facilities closed except for facilities where less than 10 persons including employees are present at one time.
	17 August 2020	Schools re-open for virtual instruction only [4].

Lagrange Health District 4

Butts County [38]	2 April 2020	Executive order to shelter in place for Georgia.
	17 August 2020	Schools re-open with virtual mode of instruction only [4].
Carroll County [39]	31 March 2020	Executive order requiring individuals with increased risk of complications from Covid-19 to isolate, quarantine or shelter in place.<br>No gatherings involving 10 or more individuals should take place, if gatherings require standing or sitting 6feet apart.
	4 May 2020	Parks re-open.
	24 August 2020	Schools re-open for in person instruction with virtual options offered [4].
Coweta County [40]	29 June 2020	Extension of state of public health emergency until 11 August 2020.<br>Governor extends social distancing guidelines until 15 July 2020.
	13 August 2020	Schools re-open for virtual instruction only [4].
Fayette County [41]	29 May 2020	Shelter in place order for citizens >65 and those with underlying health conditions.<br>Gatherings of 25 or more persons banned unless social distancing measures are in place (increased from 10).<br>Bars re-open with strict guidelines.<br>Overnight camps allowed with restrictions.<br>Restrictions for businesses that have been allowed to re-open.

	17 August 2020	Schools re-open for in-person instruction with virtual options offered [4].
Heard County [1]	23 March 2020	Executive order requiring individuals with increased risk of complications from Covid-19 to isolate, quarantine or shelter in place.<br>No gatherings involving 10 or more individuals should take place, if gatherings require standing or sitting 6feet apart; order expiring 6 April 2020.<br>Expanding temporary licensing of certain medical professions to assist the states response to the spread of Covid-19.
	5 August 2020	Schools re-open with in-person with virtual option beginning 12 August [4].
Henry County [42]	23 March 2020	Indoor and outdoor sitting capacity reduced to 10 persons at one time, additionally, a 6-foot distance should be maintained at all times. All publicly owned active recreational facilities closed until further notice.
	17 August 2020	Schools re-open with virtual instruction [4].
Lamar County [1]	23 March 2020	Executive order requiring individuals with increased risk of complications from Covid-19 to isolate, quarantine or shelter in place.<br>No gatherings involving 10 or more individuals should take place, if gatherings require standing or sitting 6feet apart; order Expiring 6 April 2020.
	2 April 2020	State-wide shelter-in-place executive order. Executive order to limit physical interactions including shelter-in-place if diagnosed with underlying conditions, closure of bars, and no gatherings involving 10 or more individuals should take place.
	12 August 2020	Schools re-open in-person instruction with virtual option [4].
Meriwether County [43]	29 June 2020	State of public health emergency extended through 11 August 2020.<br>Social distancing continues, ban on gatherings of more than 50 people unless there is 6ft between each person.<br>Sheltering in place for those living in long-term care facilities and medically fragile.<br>Rules for school re-opening.
	24 August 2020	Schools re-open for in-person instruction with virtual option offered [4]..
Pike County [44]	6 July 2020	Covid-19 executive order extended. Public health state of emergency and existing Covid-19 safety measures.
	11 August 2020	Schools re-open for in-person instruction with virtual option offered [4].

Spalding County [1]	23 March 2020	Prohibition of private or public gatherings of more than 10 individuals. Social distancing measures must be in place for gathering with over 10 individuals.<br>Closure of indoor recreational facilities and business requiring sustained physical contact.<br>Countywide shelter in place for persons with underlying conditions likely to increase spread of Covid-19.
	17 August 2020	Schools re-open for virtual instructions only [4].
Troup County [1]	23 March 2020	Declaration of state of emergency. Activation of county emergency operations plan and adoption of emergency management ordinances.
	17 August 2020	Schools re-open for in-person instruction with virtual option [4].
Upson County [1]	23 March 2020	Closures of non-essential businesses providing body care not supervised under a licensed medical professional.<br>Closure of indoor and outdoor recreation, fitness, and entertainment facilities.<br>Prohibition of gatherings involving more than 10 persons at one time.
	5 August 2020	Schools began re-opening for both in-person and virtual instruction [2].

South Central Health District 5-1

Bleckley County [45]	23 March 2020	Curfew from 10:00pm until 6:00am with few exceptions.
	5 August 2020	Schools began re-opening for both in-person and virtual instruction [2].
Dodge County [46]	23 March 2020	Executive order requiring individuals with increased risk of complications from Covid-19 to isolate, quarantine or shelter in place.<br>No gatherings involving 10 or more individuals should take place, if gatherings require standing or sitting 6 feet apart.
	5 August 2020	Schools began re-opening for both in-person and virtual instruction [2].
Johnson County [1]	23 March 2020	State of emergency declared. Indoor and outdoor sitting capacity reduced to 10 persons at one time, additionally, a 6-foot distance should be maintained at all times. All publicly owned active recreational facilities closed until further notice.
	5 August 2020	Schools began re-opening for both in-person and virtual instruction [2].
Laurens County [47]	2 April 2020	Statewide shelter in place order to stop the spread of Covid-19.
	5 August 2020	Schools began re-opening for both in-person and virtual instruction [2].

Montgomery County [48]	17 June 2020	Montgomery county buildings open to public as long as CDC guidelines are followed to protect residents from Covid-19.
	5 August 2020	Schools began re-opening for both in-person and virtual instruction [2].
Pulaski County [49]	23 March 2020	Indoor and outdoor sitting capacity reduced to 10 persons at one time, additionally, a 6-foot distance should be maintained at all times. All publicly owned active recreational facilities closed until further notice.<br>County wide curfew from 10:00pm until 6:00am.
	5 August 2020	Schools began re-opening for both in-person and virtual instruction [2].
Telfair County [50]	23 March 2020	Executive order requiring individuals with increased risk of complications from Covid-19 to isolate, quarantine or shelter in place.<br>No gatherings involving 10 or more individuals should take place, if gatherings require standing or sitting 6feet apart; order expiring 6 April 2020.
	2 April 2020	State-wide shelter-in-place executive order. Executive order to limit physical interactions including shelter-in-place if diagnosed with underlying conditions, closure of bars, and no gatherings involving 10 or more individuals should take place.
	5 August 2020	Schools began re-opening for both in-person and virtual instruction [2].
Treutlen County [51]	13 May 2020	Voluntary stay safe at home.<br>Prohibition of gatherings involving more than 10 persons at one time unless there is 6ft distance between each person.<br>Recreation and entertainment facilities closed except for facilities where less than 10 persons including employees are present at one time.
	5 August 2020	Schools began re-opening for both in-person and virtual instruction [2].
Wheeler County [1]	23 March 2020	Limited number of persons at one time at retail stores-grocery/non-grocery, pharmaceutical, and building supplies.<br>Close down of all indoor dining room facilities.<br>Countywide curfew between 9:00pm until 6:00am.<br>Gatherings limited to 10 individuals.<br>Cancellation of in-house services at churches.<br>Mandatory social distancing of 6-foot.
	5 August 2020	Schools began re-opening for both in-person and virtual instruction [2].

Wilcox County [1]	23 March 2020	Prohibition of private or public gatherings of more than 10 individuals. Social distancing measures must be in place for gathering with over 10 individuals.<br>Closure of indoor recreational facilities and business requiring sustained physical contact.<br>Countywide shelter in place for persons with underlying conditions likely to increase spread of Covid-19.
	5 August 2020	Schools began re-opening for both in-person and virtual instruction [2].

North Central Health District 5-2

Baldwin County [52]	11 May 2020	All residents and visitors adhere to Baldwin County guidelines provided by CDC.
	5 August 2020	Schools began re-opening for both in-person and virtual instruction [2].
Bibb County [53]	9 July 2020	Members of the public should wear face coverings as often as possible especially when in close proximity with others.
	5 August 2020	Schools began re-opening for both in-person and virtual instruction [2].
Crawford County [54]	23 March 2020	State of emergency declared. Indoor and outdoor sitting capacity reduced to 10 persons at one time, additionally, a 6-foot distance should be maintained at all times. All publicly owned active recreational facilities closed until further notice.
	5 August 2020	Schools began re-opening for both in-person and virtual instruction [2].
Hancock County [55]	24 March 2020	Prohibition of private or public gatherings of more than 10 individuals. Social distancing measures must be in place for gathering with over 10 individuals.<br>Curfew imposed on non-essential travels from 10:00pm to 6:00am.<br>Closure of indoor recreational facilities and business requiring sustained physical contact.<br>Countywide shelter in place for persons with underlying conditions likely to increase spread of Covid-19.
	5 August 2020	Schools began re-opening for both in-person and virtual instruction [2].
Houston County [56]	29 May 2020	Public health state of emergency extended through August 11, 2020.<br>Order requiring social distancing, ban on gathering of more than 50 people unless there is 6ft distance between each person.<br>Shelter in place order for those living in long- term care facilities and the medically fragile.

	5 August 2020	Schools began re-opening for both in-person and virtual instruction [2].
Jasper County [57]	23 March 2020	Indoor and outdoor sitting capacity reduced to 10 persons at one time, additionally, a 6-foot distance should be maintained at all times. All publicly owned active recreational facilities closed until further notice.
	3 April 2020<br><br>26 August 2020	State-wide shelter-in-place executive order<br><br>Schools re-open with in-person instruction and virtual options offered: original start date was 12 August but delayed 2 weeks [4].
Jones County [58]	23 March 2020	Executive order requiring individuals with increased risk of complications from Covid-19 to isolate, quarantine or shelter in place.<br>No gatherings involving 10 or more individuals should take place, if gatherings require standing or sitting 6feet apart; order expiring 6 April 2020.
	2 April 2020	State-wide shelter-in-place executive order. Executive order to limit physical interactions including shelter-in-place if diagnosed with underlying conditions and closure of bars.
	5 August 2020	Schools began re-opening for both in-person and virtual instruction [2].
Monroe County [59]	13 May 2020	Re-opening measures in place.
	5 August 2020	Schools began re-opening for both in-person and virtual instruction [2].
Peach County [60]	24 March 2020	Limited number of persons at one time at retail stores-grocery/non-grocery, pharmaceutical, and building supplies.<br>Close down of all indoor dining room facilities.<br>All businesses ordered to close indoor access to general public daily by 10:00pm.<br>Gatherings limited to 10 individuals.<br>Cancellation of in-house services at churches.<br>Mandatory social distancing of 6-foot.
	5 August 2020	Schools began re-opening for both in-person and virtual instruction [2].
Putnam County [61]	3 April 2020	Statewide shelter in place order.
	31 August 2020	Schools begin in-person instruction with virtual options offered [4].
Twiggs County [62]	9 April 2020	Shelter in place and social distancing measures remain in place until 13 May 2020 unless extended or terminated by executive order.

	5 August 2020	Schools began re-opening for both in-person and virtual instruction [2].
Washington County [63]	15 July 2020	All county offices closed to the public indefinitely due to Covid-19.
	5 August 2020	Schools began re-opening for both in-person and virtual instruction [2].
Wilkinson County [1]	19 March 2020	Executive order requiring individuals with increased risk of complications from Covid-19 to isolate, quarantine or shelter in place. No gatherings involving 10 or more individuals should take place if gatherings require standing or sitting 6 feet apart.
	5 August 2020	Schools began re-opening for both in-person and virtual instruction [2].

East Central Health District 6

Burke County [64]	1 April 2020	Social gatherings of 50 or more cancelled for a two-week period unless 6ft distance is maintained. Funerals shall be conducted only as private graveside services with 10 or less persons in attendance.<br>Restaurants open to take-out, drive through or curbside service.
	5 August 2020	Schools began re-opening for both in-person and virtual instruction [2].
Columbia County [65]	18 June 2020	Professional sports teams and organizations must follow the rules and guidelines set by respective leagues.<br>Shelter in place for residents >65 no longer required unless they are in long term care facilities or medically fragile.<br>No party maximum for people who can sit together in restaurants and dining rooms.<br>Live performances may re-open for business if compliant with specific criteria.
	5 August 2020	Schools began re-opening for both in-person and virtual instruction [2].
Emanuel County [66]	1 April 2020	All public and private gatherings of greater than 10 people occurring outside the household prohibited.<br>Grocery stores, pharmacies, and other businesses remain open during emergency but must inform consumers to maintain 6 ft personal distance, more than 10 people shall not be allowed at one time.<br>Restaurants open to take-out, drive through or curbside service.
	5 August 2020	Schools began re-opening for both in-person and virtual instruction [2].

Glascock County [1]	29 May 2020	Shelter in place order for citizens >65 and those with underlying health conditions.<br>Gatherings of 25 or more persons banned unless social distancing measures are in place (increased from 10).<br>Bars re-open with strict guidelines.<br>Overnight camps allowed with restrictions.<br>Restrictions for businesses that have been allowed to re-open.
	5 August 2020	Schools began re-opening for both in-person and virtual instruction [2].
Jefferson County [1]	23 March 2020	Executive order requiring individuals with increased risk of complications from Covid-19 to isolate, quarantine or shelter in place.<br>No gatherings involving 10 or more individuals should take place, if gatherings require standing or sitting 6feet apart; order expiring 6 April 2020.<br>Expanding temporary licensing of certain medical professions to assist the states response to the spread of Covid-19.
	31 July 2020	Schools re-open with in-person instruction [4].
Jenkins County [1]	23 March 2020	Indoor and outdoor sitting capacity reduced to 10 persons at one time, additionally, a 6-foot distance should be maintained at all times. All publicly owned active recreational facilities closed until further notice.
	5 August 2020	Schools began re-opening for both in-person and virtual instruction [2].
Lincoln County [1]	23 March 2020	Executive order requiring individuals with increased risk of complications from Covid-19 to isolate, quarantine or shelter in place.<br>No gatherings involving 10 or more individuals should take place, if gatherings require standing or sitting 6feet apart; order expiring 6 April 2020.
	2 April 2020<br><br>August 5, 2020	State-wide shelter-in-place executive order. Executive order to limit physical interactions including shelter-in-place if diagnosed with underlying conditions, closure of bars, and no gatherings involving 10 or more individuals should take place.<br>Schools began re-opening for both in-person and virtual instruction [2].
McDuffie County [67]	2 April 2020	Prohibition of gatherings involving more than 10 persons at one time.<br>Recreation and entertainment facilities closed except for facilities where less than 10 persons including employees are present at one time.

	5 August 2020	Schools began re-opening for both in-person and virtual instruction [2].
Richmond County [68]	24 June 2020	Face coverings in public area.
	5 August 2020	Schools began re-opening for both in-person and virtual instruction [2].
Screven County [1]	23 March 2020	Prohibition of private or public gatherings of more than 10 individuals. Social distancing measures must be in place for gathering with over 10 individuals.<br>Closure of indoor recreational facilities and business requiring sustained physical contact.<br>Countywide shelter in place for persons with underlying conditions likely to increase spread of Covid-19.
	5 August 2020	Schools began re-opening for both in-person and virtual instruction [2].
Taliaferro County [69]	4 April 2020	Shelter in place order through 13 April 2020.<br>Closure of gyms, fitness centers, bowling allies, theaters, live performance venues, amusement parks, hair designers, beauty shops, cosmetology schools, barbershops and massage therapist. Dine-in service at restaurants and social clubs no longer permitted.
	5 August 2020	Schools began re-opening for both in-person and virtual instruction [2].
Warren County [70]	2 April 2020	Shelter in place order through 13 April 2020.<br>Closure of gyms, fitness centers, bowling allies, theaters, live performance venues, amusement parks, hair designers, beauty shops, cosmetology schools, barbershops and massage therapist. Dine-in service at restaurants and social clubs no longer permitted.
	5 August 2020	Schools began re-opening for both in-person and virtual instruction [2].
Wilkes County [71]	15 July 2020	Enforcing social distancing at all times.<br>Shelter in place order for residents in long term facilities and are 'medically fragile'.
	5 August 2020	Schools began re-opening for both in-person and virtual instruction [2].

West Central Health District 7

Chattahoochee County [72]	25 March 2020	County halts public access to buildings, facilities through 21 April 2020.
	5 August 2020	Schools began re-opening for both in-person and virtual instruction [2].

Clay County [73]	23 March 2020	Executive order requiring individuals with increased risk of complications from Covid-19 to isolate, quarantine or shelter in place.<br>No gatherings involving 10 or more individuals should take place, if gatherings require standing or sitting 6 feet apart.
	5 August 2020	Schools began re-opening for both in-person and virtual instruction [2].
Crisp County [74]	20 March 2020	Voluntary shelter in place.<br>Prohibition on gatherings.<br>A restaurant shall immediately limit its occupancy at 50% of its current building occupancy.
	5 August 2020	Schools began re-opening for both in-person and virtual instruction [2].
Dooly County[1]	23 March 2020	Executive order requiring individuals with increased risk of complications from Covid-19 to isolate, quarantine or shelter in place.<br>No gatherings involving 10 or more individuals should take place, if gatherings require standing or sitting 6feet apart.
	5 August 2020	Schools began re-opening for both in-person and virtual instruction [2].
Harris County [75]	16 March 2020	Countywide Shelter-In Place Order.<br>School closure.<br>Prohibition against overcharging for goods, material, services and housing.<br>Public or private gatherings. Indoor or outdoor gatherings of 10 persons or less maybe permitted while maintaining 6feet distance.<br>Food serving establishments cease offering dine-in services, may continue offering food to customers via delivery, drive through or take-out.<br>Restricted access to public buildings, parks and facilities.
	5 August 2020	Schools began re-opening for both in-person and virtual instruction [2].
Macon County [1]	23 March 2020	Indoor and outdoor sitting capacity reduced to 10 persons at one time, additionally, a 6-foot distance should be maintained at all times. All publicly owned active recreational facilities closed until further notice.
	5 August 2020	Schools began re-opening for both in-person and virtual instruction [2].
Muscogee County [1]	23 March 2020	Executive order requiring individuals with increased risk of complications from Covid-19 to isolate, quarantine or shelter in place.<br>No gatherings involving 10 or more individuals should take place, if gatherings require standing or sitting 6feet apart; order expiring 6 April 2020.

	2 April 2020	State-wide shelter-in-place executive order. Executive order to limit physical interactions including shelter-in-place if diagnosed with underlying conditions, closure of bars, and no gatherings involving 10 or more individuals should take place.
	5 August 2020	Schools began re-opening for both in-person and virtual instruction [2].
Marion County [76]	28 May 2020	Shelter in place order for citizens >65 and those with underlying health conditions.<br>Gatherings of 25 or more persons banned unless social distancing measures are in place (increased from 10).<br>Bars re-open with strict guidelines.<br>Overnight camps allowed with restrictions.<br>Restrictions for businesses that have been allowed to re-open.
	5 August 2020	Schools began re-opening for both in-person and virtual instruction [2].
Quitman County [77]	8 July 2020	Curfew imposed from 11:00pm to 6:00am effective immediately until 22 July 2020 except exempt individuals.
	5 August 2020	Schools began re-opening for both in-person and virtual instruction [2].
Randolph County [1]	23 March 2020	Prohibition of private or public gatherings of more than 10 individuals. Social distancing measures must be in place for gathering with over 10 individuals.<br>Closure of indoor recreational facilities and business requiring sustained physical contact.<br>Countywide shelter in place for persons with underlying conditions likely to increase spread of Covid-19.
	5 August 2020	Schools began re-opening for both in-person and virtual instruction [2].
Schley County [1]	23 March 2020	Declaration of state of emergency. Activation of county emergency operations plan and adoption of emergency management ordinances.
	5 August 2020	Schools began re-opening for both in-person and virtual instruction [2].
Stewart County [1]	23 March 2020	Closures of non-essential businesses providing body care not supervised under a licensed medical professional.<br>Closure of indoor and outdoor recreation, fitness, and entertainment facilities.<br>Prohibition of gatherings involving more than 10 persons at one time.
	5 August 2020	Schools began re-opening for both in-person and virtual instruction [2].

Sumter County [1]	23 March 2020	Executive order requiring individuals with increased risk of complications from Covid-19 to isolate, quarantine or shelter in place.<br>No gatherings involving 10 or more individuals should take place if gatherings require standing or sitting 6feet apart.
	5 August 2020	Schools began re-opening for both in-person and virtual instruction [2].
Talbot County [78]	31 March 2020	Executive order requiring individuals with increased risk of complications from Covid-19 to isolate, quarantine or shelter in place.<br>No gatherings involving 10 or more individuals should take place if gatherings require standing or sitting 6feet apart.<br>State of emergency declared until 30 April 2020.<br>Take-out, drive-through or delivery at places where food is offered.<br>All bars and private social clubs are closed.<br>Restriction within county within the hours of 10pm and 6am with some exemptions.
	5 August 2020	Schools began re-opening for both in-person and virtual instruction [2].
Taylor County [1]	23 March 2020	Immediate closure of indoor and outdoor recreation, fitness and entertainment facilities.<br>Businesses shall allow no more than 10 persons to be gathered at a single location.<br>Cessation of dine-in services.
	5 August 2020	Schools began re-opening for both in-person and virtual instruction [2].
Webster County [79]	30 March 2020	Immediate closure of indoor and outdoor recreation, fitness and entertainment facilities.<br>Businesses shall allow no more than 10 persons to be gathered at a single location.<br>Cessation of dine-in services.<br>No worship services if attended by more than 10 persons.<br>Providers of 'essential services' not affected by the order.<br>Curfew between the hours of 9pm and 5am. Fine of $500 for anyone who violates.
	5 August 2020	Schools began re-opening for both in-person and virtual instruction [2].

South health district (8-1)

Ben Hill County [80]	23 March 2020	No gatherings involving 10 or more individuals should take place, if gatherings require standing or sitting 6feet apart; order expiring 6 April 2020.

	28 May 2020	Extension of state of public health emergency, to expire on 12 June 2020, to 12 July 2020.<br>Guidance for reviving a healthy Georgia in response to Covid-19
	5 August 2020	Schools began re-opening for both in-person and virtual instruction [2].
Berrien County [81]	23 March 2020	Executive order requiring individuals with increased risk of complications from Covid-19 to isolate, quarantine or shelter in place.<br>No gatherings involving 10 or more individuals should take place, if gatherings require standing or sitting 6feet apart.
	13 May 2020	Non-critical businesses, establishments, corporation, or organizations that continue in-person operations shall meet minimum criteria to be open.<br>Retail businesses including food establishments must meet minimum criteria to remain open.
	5 August 2020	Schools began re-opening for both in-person and virtual instruction [2].
Brooks County [82]	30 April 2020	Shelter in place through 12 June 2020 for individuals with increased risk of complications from Covid-19.
	13 May 2020	Executive order requiring individuals with increased risk of complications from Covid-19 to isolate, quarantine or shelter in place.<br>No gatherings involving 10 or more individuals should take place, if gatherings require standing or sitting 6feet apart.
	5 August 2020	Schools began re-opening for both in-person and virtual instruction [2].
Cook County [83]	25 March 2020<br><br><br><br><br><br><br><br><br><br><br><br><br>5 August 2020	Shelter in place order for citizens >65, those with underlying health conditions and persons in nursing homes and long-term care facilities.<br>Gatherings of 25 or more persons banned unless social distancing measures are in place (increased from 10).<br>Voluntary stay safe at home policy.<br>Curfew imposed from 9:00pm to 5:00am for residents with exception for "exempt individuals".<br>Prohibition of gatherings of more than 10 persons.<br>Closure of recreational and entertainment facilities including gyms and fitness centers.<br>Suspension of all on-premises food and drinks consumption at restaurants.<br>Schools began re-opening for both in-person and virtual instruction [2].
Echols County [84]	1 April 2020	Statewide Shelter-In Place Order.

	5 August 2020	Schools began re-opening for both in-person and virtual instruction [2].
Irwin County [1]	23 March 2020	Executive order requiring individuals with increased risk of complications from Covid-19 to isolate, quarantine or shelter in place.<br>No gatherings involving 10 or more individuals should take place, if gatherings require standing or sitting 6feet apart; order expiring 6 April 2020.<br>Expanding temporary licensing of certain medical professions to assist the states response to the spread of Covid-19.
	5 August 2020	Schools began re-opening for both in-person and virtual instruction [2].
Lanier County [85]	23 March 2020	Executive order requiring individuals with increased risk of complications from Covid-19 to isolate, quarantine or shelter in place.<br>No gatherings involving 10 or more individuals should take place, if gatherings require standing or sitting 6feet apart; order expiring 6 April 2020.
	1 April 2020	State-wide shelter-in-place executive order. Executive order to limit physical interactions including shelter-in-place if diagnosed with underlying conditions, closure of bars, and no gatherings involving 10 or more individuals should take place.
	5 August 2020	Schools began re-opening for both in-person and virtual instruction [2].
Lowndes County [86]	2 April 2020	Statewide Shelter-In Place Order.<br>Voluntary stay safe at home.<br>Prohibition of gatherings involving more than 10 persons at one time.
	5 August 2020	Schools began re-opening for both in-person and virtual instruction [2].
Tift County [87]	24 March 2020	Limited number of persons at one time at retail stores-grocery/non-grocery, pharmaceutical, and building supplies.<br>Close down of all indoor dining room facilities.<br>Shelter in place order.<br>Countywide curfew between 8:00pm until 5:00am for all residents unless "exempt individuals".<br>Gatherings limited to 10 individuals.<br>Cancellation of in-house services at churches.<br>Mandatory social distancing of 6-foot.
	5 August 2020	Schools began re-opening for both in-person and virtual instruction [2].

Turner County [88]	24 March 2020	Prohibition of private or public gatherings of more than 10 individuals. Social distancing measures must be in place for gathering with over 10 individuals.<br>Closure of indoor recreational facilities and business requiring sustained physical contact.<br>Countywide shelter in place for persons with underlying conditions likely to increase spread of Covid-19.
	5 August 2020	Schools began re-opening for both in-person and virtual instruction [2].

Southwest health district (8-2)

Baker County [1]	23 March 2020	Executive order requiring individuals with increased risk of complications from Covid-19 to isolate, quarantine or shelter in place.<br>No gatherings involving 10 or more individuals should take place, if gatherings require standing or sitting 6feet apart; order expiring 6 April 2020.<br>Expanding temporary licensing of certain medical professions to assist the states response to the spread of Covid-19.
	5 August 2020	Schools began re-opening for both in-person and virtual instruction [2].
Calhoun County [89]	23 March 2020	Executive order requiring individuals with increased risk of complications from Covid-19 to isolate, quarantine or shelter in place. Shelter in place order for citizens >65 and those with underlying health conditions.<br>Prohibition of private or public gatherings of more than 10 individuals. Social distancing measures must be in place for gathering with over 10 individuals.
	11 June 2020	Gatherings of 25 or more persons banned unless social distancing measures are in place (increased from 10).<br>Bars re-open with strict guidelines.<br>Overnight camps allowed with restrictions.<br>Restrictions for businesses that have been allowed to re-open.
	12 August 2020	Schools re-opened in-person mode of instruction with virtual options [4].
Colquitt County [1]	23 March 2020	Executive order requiring individuals with increased risk of complications from Covid-19 to isolate, quarantine or shelter in place.<br>No gatherings involving 10 or more individuals should take place, if gatherings require standing or sitting 6feet apart; order expiring 6 April 2020.<br>Expanding temporary licensing of certain medical professions to assist the states response to the spread of Covid-19.
	5 August 2020	Schools began re-opening for both in-person and virtual instruction [2].

Decatur County [1]	23 March 2020	Executive order requiring individuals with increased risk of complications from Covid-19 to isolate, quarantine or shelter in place.<br>No gatherings involving 10 or more individuals should take place, if gatherings require standing or sitting 6feet apart; order expiring 6 April 2020.<br>Expanding temporary licensing of certain medical professions to assist the states response to the spread of Covid-19.
	17 August 2020	Schools re-open for virtual instruction only [4].
Dougherty County [90]	25 March 2020	Statewide Shelter-In Place Order.<br>Public or private gatherings. Indoor or outdoor gatherings of 10 persons or less maybe permitted while maintaining 6feet distance.<br>Food serving establishments cease offering dine-in services, may continue offering food to customers via delivery, drive through or take-out.<br>Public buildings, parks and facilities restrictions.
	5 August 2020	Schools began re-opening for both in-person and virtual instruction [2].
Early County [91]	16 June 2020	Shelter in place order no longer required for residents and visitors of Georgia who are 65 or older unless they meet certain criteria.<br>Gatherings of more than 50 people banned unless 6 feet distance is maintained.<br>No longer party maximum for the number of people who can sit together at a restaurant.
	5 August 2020	Schools began re-opening for both in-person and virtual instruction [2].
Grady County [92]	28 March 2020	Executive order requiring individuals with increased risk of complications from Covid-19 to isolate, quarantine or shelter in place.<br>No gatherings involving 10 or more individuals should take place, if gatherings require standing or sitting 6feet apart; order expiring 6 April 2020.<br>Countywide curfew between 10:00pm until 5:00am.
	5 August 2020	Schools began re-opening for both in-person and virtual instruction [2]
Lee County [93]	24 March 2020	Voluntary stay safe at home.<br>Prohibition of gatherings involving more than 10 persons at one time.<br>Recreation and entertainment facilities closed except for facilities where less than 10 persons including employees are present at one time.<br>Mandatory curfew between 10:00pm and 6:00am.
	5 August 2020	Schools began re-opening for both in-person and virtual instruction [2].

Miller County [1]	23 March 2020	Executive order requiring individuals with increased risk of complications from Covid-19 to isolate, quarantine or shelter in place.<br>No gatherings involving 10 or more individuals should take place, if gatherings require standing or sitting 6feet apart; order expiring 6 April 2020.<br>Expanding temporary licensing of certain medical professions to assist the states response to the spread of Covid-19.
	5 August 2020	Schools began re-opening for both in-person and virtual instruction [2].
Mitchell County [94]	23 March 2020	Prohibition of private or public gatherings of more than 10 individuals. Social distancing measures must be in place for gathering with over 10 individuals.<br>Closure of indoor recreational facilities and business requiring sustained physical contact.<br>Countywide shelter in place for persons with underlying conditions likely to increase spread of Covid-19.
	5 August 2020	Schools began re-opening for both in-person and virtual instruction [2].
Seminole County [95]	25 March 2020	Declaration of state of emergency. Activation of county emergency operations plan and adoption of emergency management ordinances.<br>No gatherings involving 10 or more individuals should take place, if gatherings require standing or sitting 6feet apart; order
	5 August 2020	Schools began re-opening for both in-person and virtual instruction [2].
Terrell County [96]	2 April 2020	Closures of non-essential businesses providing body care not supervised under a licensed medical professional.<br>Closure of indoor and outdoor recreation, fitness, and entertainment facilities.<br>Prohibition of gatherings involving more than 10 persons at one time.<br>Social distancing and sanitation practices.
	5 August 2020	Schools began re-opening for both in-person and virtual instruction [2].
Thomas County [1]	23 March 2020	Executive order requiring individuals with increased risk of complications from Covid-19 to isolate, quarantine or shelter in place.<br>No gatherings involving 10 or more individuals should take place if gatherings require standing or sitting 6feet apart.
	5 August 2020	Schools began re-opening for both in-person and virtual instruction [2].

Worth County[97]	24 March 2020	Executive order requiring individuals with increased risk of complications from Covid-19 to isolate, quarantine or shelter in place.<br>No gatherings involving 10 or more individuals should take place if gatherings require standing or sitting 6feet apart; order expiring 6 April 2020.
	5 August 2020	Schools began re-opening for both in-person and virtual instruction [2].

Coastal Health District (9-1)

Bryan County [98]	24 March 2020	State of emergency declared. Indoor and outdoor sitting capacity reduced to 10 persons at one time, additionally, a 6-foot distance should be maintained at all times. All publicly owned active recreational facilities closed until further notice. Order effective until 3 April 2020.
	5 August 2020	Schools began re-opening for both in-person and virtual instruction [2].
Camden County [99]	16 March 2020	Declaration of precautionary public health emergency, in effect until 15 April 2020.
	5 August 2020	Schools began re-opening for both in-person and virtual instruction [2].
Chatham County [100]	23 March 2020	Executive order requiring individuals with increased risk of complications from Covid-19 to isolate, quarantine or shelter in place.
	7 April 2020	Shelter in place order for all residents and visitors.<br>Social distancing practices.
	12 May 2020	Reviving Georgia order allowing swimming pools to be reopened.
	5 August 2020	Schools began re-opening for both in-person and virtual instruction [2].
Glynn County [101]	25 March 2020	Closures of non-essential businesses providing body care not supervised under a licensed medical professional.<br>Closure of indoor and outdoor recreation, fitness, and entertainment facilities.
	5 August 2020	Schools began re-opening for both in-person and virtual instruction [2].
Liberty County [1]	23 March 2020	Executive order requiring individuals with increased risk of complications from Covid-19 to isolate, quarantine or shelter in place.
	7 April 2020	Shelter in place order for all residents and visitors.<br>Social distancing order.
	12 May 2020	Reviving Georgia order allowing swimming pools to be reopened.
	5 August 2020	Schools began re-opening for both in-person and virtual instruction [2].

McIntosh County [1]	23 March 2020	Executive order requiring individuals with increased risk of complications from Covid-19 to isolate, quarantine or shelter in place.
	7 April 2020	Shelter in place order for all residents and visitors.<br>Social distancing order (6 feet part).
	5 August 2020	Schools began re-opening for both in-person and virtual instruction [2].
Effingham County [1]	23 March 2020	Executive order requiring individuals with increased risk of complications from Covid-19 to isolate, quarantine or shelter in place.
	7 April 2020	Shelter in place order for all residents and visitors.<br>Social distancing order.
	12 May 2020	Reviving Georgia order allowing swimming pools to be reopened.
	5 August 2020	Schools began re-opening for both in-person and virtual instruction [2].
Long County [102]	22 March 2020	Closures of non-essential businesses providing body care not supervised under a licensed medical professional through 30 April 2020.<br>Closure of indoor and outdoor recreation, fitness, and entertainment facilities.
	5 August 2020	Schools began re-opening for both in-person and virtual instruction [2].

Southeast Health District (9-2)

Appling County [103]	16 March 2020	Social gatherings of 50 or more cancelled for a two-week period. Limit on exposure to crowds for a two-week period.
	5 August 2020	Schools began re-opening for both in-person and virtual instruction [2].
Atkinson County [104]	31 March 2020	Executive order requiring individuals with increased risk of complications from Covid-19 to isolate, quarantine or shelter in place.<br>No gatherings involving 10 or more individuals should take place, if gatherings require standing or sitting 6feet apart.
	5 August 2020	Schools began re-opening for both in-person and virtual instruction [2].
Bacon County [105]	25 March 2020	State of emergency declared. Indoor and outdoor sitting capacity reduced to 10 persons at one time, additionally, a 6-foot distance should be maintained at all times. All publicly owned active recreational facilities closed until further notice.
	5 August 2020	Schools began re-opening for both in-person and virtual instruction [2].

Brantley County [106]	29 May 2020	Shelter in place order for citizens >65 and those with underlying health conditions.<br>Gatherings of 25 or more persons banned unless social distancing measures are in place (increased from 10).<br>Bars re-open with strict guidelines.<br>Overnight camps allowed with restrictions.<br>Restrictions for businesses that have been allowed to re-open.
	5 August 2020	Schools began re-opening for both in-person and virtual instruction [2].
Bulloch County [107]	31 March 2020	Statewide Shelter-In Place Order.<br>Curfew throughout Bulloch County from 10:00pm to 6:00pm.<br>Prohibition against overcharging for goods, material, services and housing.<br>Public or private gatherings. Indoor or outdoor gatherings of 10 persons or less maybe permitted while maintaining 6feet distance.<br>Food serving establishments cease offering dine-in services, may continue offering food to customers via delivery, drive through or take-out.<br>Public buildings, parks and facilities restrictions.
	5 August 2020	Schools began re-opening for both in-person and virtual instruction [2].
Candler County [108]	30 March 2020	Indoor and outdoor sitting capacity reduced to 10 persons at one time, additionally, a 6-foot distance should be maintained at all times. All publicly owned active recreational facilities closed until further notice.
	5 August 2020	Schools began re-opening for both in-person and virtual instruction [2].
Charlton County [109]	23 March 2020	Executive order requiring individuals with increased risk of complications from Covid-19 to isolate, quarantine or shelter in place.<br>No gatherings involving 10 or more individuals should take place, if gatherings require standing or sitting 6feet apart; order expiring 6 April 2020.
	2 April 2020	State-wide shelter-in-place executive order. Executive order to limit physical interactions including shelter-in-place if diagnosed with underlying conditions, closure of bars, and no gatherings involving 10 or more individuals should take place.
	8 September 2020	Schools re-open in person with virtual options option [4].

Clinch County [110]	31 March 2020	Voluntary stay safe at home.<br>Prohibition of gatherings involving more than 10 persons at one time.<br>Recreation and entertainment facilities closed except for facilities where less than 10 persons including employees are present at one time.
	5 August 2020	Schools began re-opening for both in-person and virtual instruction [2].
Coffee County [111]	25 March 2020	Limited number of persons at one time at retail stores-grocery/non-grocery, pharmaceutical, and building supplies.<br>Close down of all indoor dining room facilities.<br>Countywide curfew between 9:00pm until 6:00am.<br>Gatherings limited to 10 individuals.<br>Cancellation of in-house services at churches.<br>Mandatory social distancing of 6-foot.
	5 August 2020	Schools began re-opening for both in-person and virtual instruction [2].
Evans County [112]	24 March 2020	Prohibition of private or public gatherings of more than 10 individuals. Social distancing measures must be in place for gathering with over 10 individuals.<br>Closure of indoor recreational facilities and business requiring sustained physical contact.<br>Countywide shelter in place for persons with underlying conditions likely to increase spread of Covid-19.
	5 August 2020	Schools began re-opening for both in-person and virtual instruction [2].
Jeff Davis County [113]	26 March 2020	Declaration of state of emergency. Activation of county emergency operations plan and adoption of emergency management ordinances.
	5 August 2020	Schools began re-opening for both in-person and virtual instruction [2].
Pierce County [114]	25 March 2020	Closures of non-essential businesses providing body care not supervised under a licensed medical professional.<br>Closure of indoor and outdoor recreation, fitness, and entertainment facilities.<br>Prohibition of gatherings involving more than 10 persons at one time.
	5 August 2020	Schools began re-opening for both in-person and virtual instruction [2].
Tattnall County [115]	19 March 2020	Executive order requiring individuals with increased risk of complications from Covid-19 to isolate, quarantine or shelter in place.

		No gatherings involving 10 or more individuals should take place if gatherings require standing or sitting 6feet apart.
	5 August 2020	Schools began re-opening for both in-person and virtual instruction [2].
Toombs County [116]	24 March 2020	Executive order requiring individuals with increased risk of complications from Covid-19 to isolate, quarantine or shelter in place.<br>No gatherings involving 10 or more individuals should take place if gatherings require standing or sitting 6feet apart; order expiring 6 April 2020.
	5 August 2020	Schools began re-opening for both in-person and virtual instruction [2].
Ware County [117]	26 March 2020	Immediate closure of indoor and outdoor recreation, fitness and entertainment facilities.<br>Businesses shall allow no more than 10 persons to be gathered at a single location.<br>Cessation of dine-in services.<br>No worship services if attended by more than 10 persons.
	5 August 2020	Schools began re-opening for both in-person and virtual instruction [2].
Wayne County [118]	24 March 2020	Immediate closure of indoor and outdoor recreation, fitness and entertainment facilities.<br>Businesses shall allow no more than 10 persons to be gathered at a single location.<br>Cessation of dine-in services.<br>No worship services if attended by more than 10 persons.<br>Providers of 'essential services' not affected by the order.
	5 August 2020	Schools began re-opening for both in-person and virtual instruction [2].

Northeast Health District 10

Barrow County [1]	23 March 2020	Executive order requiring individuals with increased risk of complications from Covid-19 to isolate, quarantine or shelter in place.<br>No gatherings involving 10 or more individuals should take place, if gatherings require standing or sitting 6feet apart; order expiring 6 April 2020.
	17 August 2020	Schools re-open with virtual mode of instruction only [4].
Clarke County [119]	7 July 2020	Wearing of facial covering required when in a public area.<br>All restaurants and businesses and pharmacies must require their employees to wear a facial covering at all times while having face-face interaction with the public.

	8 September 2020	School re-open with virtual mode of instruction only [4].
Elbert County [1]	2 April 2020	State-wide shelter-in-place executive order. Executive order to limit physical interactions including shelter-in-place if diagnosed with underlying conditions, closure of bars, and no gatherings involving 10 or more individuals should take place.
	5 August 2020	Schools began re-opening for both in-person and virtual instruction [2].
Greene County [120]	29 May 2020	Shelter in place order for citizens >65 and those with underlying health conditions.<br>Gatherings of 25 or more persons banned unless social distancing measures are in place (increased from 10).<br>Bars re-open with strict guidelines.<br>Overnight camps allowed with restrictions.<br>Restrictions for businesses that have been allowed to re-open.
	17 August 2020	Schools re-open in-person rotating schedule for PK-3, Virtual learning for grades 4-12 [4].
Jackson County [121]	11 June 2020	Campers and workers shall be tested for Covid-19 prior to beginning an overnight summer camp.<br>Shelter in place order for residents and visitors who are medically fragile or living in long term care facilities.
	12 August 2020	Schools re-open in person with virtual option [4].
Madison County [122]	3 April 2020	State-wide shelter-in-place executive order. Executive order to limit physical interactions including shelter-in-place if diagnosed with underlying conditions, closure of bars, and no gatherings involving 10 or more individuals should take place.
	14 August 2020	Schools re-open in-person instruction with virtual option [4].
Morgan County [123]	29 June 2020	Renewal of public health emergency.
	6 August 2020	Schools re-open in-person instruction with virtual option [4].
Oconee County [124]	23 April 2020	Campers and workers shall be tested for Covid-19 prior to beginning an overnight summer camp.<br>Shelter in place order for residents and visitors who are medically fragile or living in long term care facilities.
	5 August 2020	School begins in-person instruction with virtual option [4].
Oglethorpe County [125]	2 April 2020	State-wide shelter-in-place executive order. Executive order to limit physical interactions including shelter-in-place if diagnosed with underlying conditions, closure of bars, and no gatherings involving 10 or more individuals should take place.
	12 August 2020	School begins in-person instruction with virtual options offered [4].
Walton County [126]	7 July 2020	Wearing of facial covering required when in a public area.<br>All restaurants and businesses and pharmacies must require their employees to wear a facial covering at all times while having face-face interaction with the public.

	5 August 2020	Schools began re-opening for both in-person and virtual instruction [2].

Table S2. Time-varying reproduction number in the state of Georgia estimated using non-overlapping time windows (median and 95% credible interval) and its change between each time window (median and 95% credible interval).

	Median Rt & 95% CrI	Median Rt Difference Percentage Changes Comparing with Previous Policy Interval & 95% CrI
Before policy A	1.9137 (1.82, 2.01)	
A→ B	1.9112 (1.84, 1.99)	−0.14 (−3.94, 3.59)
B→ C	1.2579 (1.23, 1.29)	−34.21 (−33.1, 35.3)
C→ D	0.9376 (0.921, 0.955)	−25.46 (−25.2, −25.8)
D→ E	1.0724 (1.07, 1.08)	14.35 (12.2, 16.3)
Beyond E	1.0296 (1.029, 1.03)	−3.99 (−3.9, −4.1)

Table S3. Time-varying reproduction number in District 1-1 estimated using non-overlapping time windows (median and 95% credible interval) and its change between each time window (median and 95% credible interval).

	Median Rt & 95% CrI	Median Rt Difference Percentage Changes Comparing with Previous Policy Interval & 95% CrI
Before policy A	1.6153 (1.36, 1.89)	
A→ B	1.5386 (1.28, 1.82)	0.05 (0.038, 0.056)
B→ C	0.9779 (0.85, 1.12)	0.36 (0.34, 0.37)
C→ D	1.0511 (0.96, 1.15)	−0.07 (−0.24, 0.08)
D→ E	1.1097 (1.08, 1.13)	−0.054 (−0.158, 0.044)
Beyond E	1.0371 (1.02, 1.05)	0.07 (0.05, 0.085)

Table S4. Time-varying reproduction number in District 1-2 estimated using non-overlapping time windows (median and 95% credible interval) and its change between each time window (median and 95% credible interval).

	Median Rt & 95% CrI	Median Rt Difference Percentage Changes Comparing with Previous Policy Interval & 95% CrI
Before policy A	1.9007 (1.44, 2.44)	
A→ B	1.4112(1.06, 1.83)	0.26 (0.25,0.27)
B→ C	1.1471 (0.96, 1.35)	0.19 (−0.06, 0.39)
C→ D	1.2558 (1.14, 1.38)	−0.09 (−0.3, 0.085)
D→ E	1.0785 (1.05, 1.10)	0.14 (0.057, 0.23)
Beyond E	1.0415 (1.03, 1.06)	0.03 (0.01, 0.05)

Table S5. Time-varying reproduction number in District 2 estimated using non-overlapping time windows (median and 95% credible interval) and its change between each time window (median and 95% credible interval).

	Median Rt & 95%CrI	Median Rt Difference Percentage Changes Comparing with Previous Policy Interval & 95%CrI
Before policy A	2.0322 (1.5, 2.68)	
A→ B	2.5653 (2.1, 3.09)	−0.26 (−0.67, 0.07)
B→ C	1.2874 (1.16,1.43)	0.50 (0.39, 0.59)
C→ D	1.2175 (1.16,1.28)	0.05 (−0.06, 0.17)

D→ E	1.0531 (1.03, 1.07)	0.14 (0.103, 0.171)
Beyond E	1.0486 (1.04, 1.06)	0.005 (−0.014, 0.023)

Table S6. Time-varying reproduction number in District 3-1 estimated using non-overlapping time windows (median and 95% credible interval) and its change between each time window (median and 95% credible interval).

	**Median Rt & 95% CrI**	**Median Rt Difference Percentage Changes Comparing with Previous Policy Interval & 95% CrI**
Before policy A	1.6281 (1.37, 1.92)	
A→ B	1.6463 (1.43, 1.88)	−1.07 (−4.22, 1.72)
B→ C	1.1799 (1.08, 1.29)	28.41 (17.4, 38.6)
C→ D	1.0470 (0.98, 1.11)	11.30 (1.48, 20.2)
D→ E	1.0795 (1.06, 1.10)	−3.02 (−8.31, 2.26)
Beyond E	1.0303 (1.02, 1.04)	4.58 (2.96, 6.19)

Table S7. Time-varying reproduction number in District 3-2 estimated using non-overlapping time windows (median and 95% credible interval) and its change between each time window (median and 95% credible interval).

	**Median Rt & 95% CrI**	**Median Rt Difference Percentage Changes Comparing with Previous Policy Interval & 95% CrI**
Before policy A	1.6236 (1.42, 1.84)	
A→B	1.7851 (1.61, 1.79)	9.89 (1.52, 18.17)
B→C	1.1328 (1.06, 1.21)	−36.58 (−29.8, −43.0)
C→D	0.9957 (0.943, 1.05)	−12.13 (−4.4, −19.6)
D→E	1.0622 (1.05, 1.08)	6.57 (1.33, 12.02)
Beyond E	1.0309 (1.02, 1.04)	−2.95 (−2.72, −3.19)

Table S8. Time-varying reproduction number in District 3-3 estimated using non-overlapping time windows (median and 95% credible interval) and its change between each time window (median and 95% credible interval).

	**Median Rt & 95% CrI**	**Median Rt Difference Percentage Changes Comparing with Previous Policy Interval & 95% CrI**
Before policy A	1.9688 (1.39, 2.69)	
A→B	2.6340 (2.08, 3.27)	−33.37 (−84.76, 5.66)
B→C	1.1068 (0.97, 1.26)	58.09 (46.8, 67.2)
C→D	0.9750 (0.88, 1.08)	11.94 (5.1, 18.8)
D→E	1.0651 (1.03, 1.10)	−9.14 (−21.25, 2.73)
Beyond E	1.0309 (1.01, 1.06)	3.22 (2.67, 3.77)

Table S9. Time-varying reproduction number in District 3-4 estimated using non-overlapping time windows (median and 95% credible interval) and its change between each time window (median and 95% credible interval).

	**Median Rt & 95% CrI**	**Median Rt Difference Percentage Changes Comparing with Previous Policy Interval & 95% CrI**
Before policy A	2.0496 (1.68, 2.47)	
A→B	2.1294 (1.86, 2.43)	−3.58 (−27.5, 16.6)
B→C	1.1276 (1.03, 1.23)	47.07 (39.7, 54.1)
C→D	1.0819 (1.02, 1.15)	4.11 (−5.99, 13.29)
D→E	1.0656 (1.05, 1.08)	1.59 (−3.81, 6.93)

Beyond E	1.0411 (1.03, 1.05)	2.30 (0.69, 3.90)

Table S10. Time-varying reproduction number in District 3-5 estimated using non-overlapping time windows (median and 95% credible interval) and its change between each time window (median and 95% credible interval).

	**Median Rt & 95% CrI**	**Median Rt Difference Percentage Changes Comparing with Previous Policy Interval & 95% CrI**
Before policy A	1.7277 (1.45, 2.03)	
A→B	1.6921 (1.48, 1.92)	2.28 (−18.4, 19.7)
B→C	1.1594 (1.06, 1.27)	31.50 (22.0, 40.6)
C→D	1.0214 (0.96, 1.08)	11.97 (2.23, 20.75)
D→E	1.0577 (1.04, 1.08)	−3.49 (−8.40, 1.72)
Beyond E	1.0286 (1.01, 1.04)	2.75 (2.49, 3.03)

Table S11. Time-varying reproduction number in District 4 estimated using non-overlapping time windows (median and 95% credible interval) and its change between each time window (median and 95% credible interval).

	**Median Rt & 95% CrI**	**Median Rt Difference Percentage Changes Comparing with Previous Policy Interval & 95% CrI**
Before policy A	2.003 (1.69, 2.35)	
A→ B	1.7861 (1.56, 2.03)	0.11 (−0.078, 0.267)
B→ C	1.1444 (1.05, 1.24)	0.36 (0.27, 0.44)
C→ D	1.0227 (0.96, 1.08)	0.107 (0.013, 0.19)
D→ E	1.0501 (1.03, 1.07)	−0.026 (−0.07, 0.02)
Beyond E	1.0486 (1.04, 1.06)	0.002 (−0.02, 0.02)

Table S12. Time-varying reproduction number in District 5-1 estimated using non-overlapping time windows (median and 95% credible interval) and its change between each time window (median and 95% credible interval).

	**Median Rt & 95% CrI**	**Median Rt Difference Percentage Changes Comparing with Previous Policy Interval & 95% CrI**
Before policy A	1.5224 (0.68, 2.86)	
A→ B	1.5456 (0.87, 2.51)	−0.02 (−1.47, 0.55)
B→ C	1.4460 (1.11, 1.85)	0.056 (−0.78, 0.48)
C→ D	1.1782 (1.02, 1.35)	0.19 (−0.04, 0.37)
D→ E	1.1337 (1.09, 1.19)	0.04 (−0.12, 0.17)
Beyond E	0.9902 (0.96, 1.02)	0.13 (0.116, 0.138)

Table S13. Time-varying reproduction number in District 5-2 estimated using non-overlapping time windows (median and 95% credible interval) and its change between each time window (median and 95% credible interval).

	**Median Rt & 95% CrI**	**Median Rt Difference Percentage Changes Comparing with Previous Policy Interval & 95% CrI**
Before policy A	2.1274 (1.49, 2.93)	
A→ B	1.7511 (1.29, 2.31)	0.18 (−0.08, 0.38)
B→ C	1.3093 (1.14, 1.49)	0.25 (0.01, 0.45)
C→ D	1.1806 (1.09, 1.27)	0.099 (−0.02, 0.21)
D→ E	1.0653 (1.04, 1.09)	0.098 (0.029, 0.17)
Beyond E	1.0239 (1.01, 1.04)	0.04 (0.034, 0.044)

Table S14. Time-varying reproduction number in District 6 estimated using non-overlapping time windows (median and 95% credible interval) and its change between each time window (median and 95% credible interval).

	Median Rt & 95% CrI	Median Rt Difference Percentage Changes Comparing with Previous Policy Interval & 95% CrI
Before policy A	1.8275 (1.24, 2.58)	
A→ B	3.7092 (2.85, 4.72)	−1.03 (−1.66, −0.5)
B→ C	1.4797 (1.28, 1.69)	0.6 (0.49, 0.69)
C→ D	0.9611 (0.88, 1.05)	0.35 (0.25, 0.45)
D→ E	1.1162 (1.09, 1.14)	−0.16 (−0.27, −0.06)
Beyond E	1.0119 (0.99, 1.03)	0.09 (0.088, 0.098)

Table S15. Time-varying reproduction number in District 7 estimated using non-overlapping time windows (median and 95% credible interval) and its change between each time window (median and 95% credible interval).

	Median Rt & 95% CrI	Median Rt Difference Percentage Changes Comparing with Previous Policy Interval & 95% CrI
Before policy A	1.9765 (1.21, 3.01)	
A→ B	3.2833 (2.62, 4.05)	−0.67 (−1.76, −0.004)
B→ C	1.5565 (1.43, 1.69)	0.53 (0.41, 0.62)
C→ D	0.8681 (0.81, 0.93)	0.44 (0.39, 0.49)
D→ E	1.0374 (1.014, 1.06)	−0.19 (−0.28, −0.11)
Beyond E	1.0180 (0.99, 1.04)	0.018 (0.017, 0.019)

Table S16. Time-varying reproduction number in District 8-1 estimated using non-overlapping time windows (median and 95% credible interval) and its change between each time window (median and 95% credible interval).

	Median Rt & 95% CrI	Median Rt Difference Percentage Changes Comparing with Previous Policy Interval & 95% CrI
Before policy A	1.9296 (1.29, 2.76)	
A→B	1.3773 (0.94, 1.92)	28.62 (26.6, 30.2)
B→C	1.3771 (1.16, 1.62)	0.349 (−39.6, 28.2)
C→D	1.0196 (0.89, 1.15)	26.15 (11.2, 38.7)
D→E	1.0479 (1.02, 1.07)	−2.74 (−15.9, 9.78)
Beyond E	1.0379 (1.02, 1.06)	0.95 (0.64, 1.27)

Table S17. Time-varying reproduction number in District 8-2 estimated using non-overlapping time windows (median and 95% credible interval) and its change between each time window (median and 95% credible interval).

	Median Rt & 95% CrI	Median Rt Difference Percentage Changes Comparing with Previous Policy Interval & 95% CrI
Before policy A	2.1749 (1.91, 2.47)	
A→B	2.7213 (2.49, 2.96)	25.06 (8.98, 41.68)
B→C	1.0687 (1.01, 1.13)	−60.76 (−56.7, −64.5)
C→D	0.8233 (0.779, 0.869)	−22.97 (−22.9, −23.0)
D→E	1.0371 (1.01, 1.06)	25.92 (20.4, 30.8)
Beyond E	1.0164 (0.995, 1.038)	−2.01 (−1.73, −2.28)

Table S18. Time-varying reproduction number in District 9-1 estimated using non-overlapping time windows (median and 95% credible interval) and its change between each time window (median and 95% credible interval).

	Median Rt & 95% CrI	Median Rt Difference Percentage Changes Comparing with Previous Policy Interval & 95% CrI
Before policy A	1.7238 (1.11, 2.53)	
A→B	2.0167 (1.5, 2.64)	−17.42 (−95.4, 29.2)
B→C	1.2125 (1.05, 1.39)	39.97 (22.1, 54.5)
C→D	0.8828 (0.75, 1.02)	27.18 (26.7, 27.7)
D→E	1.0819 (1.06, 1.1)	−22.33 (−42.35, 4.84)
Beyond E	0.9827 (0.96, 0.99)	9.17 (9.06, 9.30)

Table S19. Time-varying reproduction number in District 9-2 estimated using non-overlapping time windows (median and 95% credible interval) and its change between each time window (median and 95% credible interval).

	Median Rt & 95% CrI	Median Rt Difference Percentage Changes Comparing with Previous Policy Interval & 95% CrI
Before policy A	2.0694 (1.03, 3.66)	
A→B	2.1816 (1.48, 3.08)	−6.62 (−130, 46.0)
B→C	1.4438 (1.25, 1.66)	34.10 (6.86, 52.82)
C→D	1.0128 (0.90, 1.13)	30.01 (17.6, 40.7)
D→E	1.0811 (1.06, 1.10)	−6.69 (−19.1, 5.20)
Beyond E	0.9909 (0.97, 1.01)	8.35 (8.03, 8.68)

Table S20. Time-varying reproduction number in District 10 estimated using non-overlapping time windows (median and 95% credible interval) and its change between each time window (median and 95% credible interval).

	Median Rt & 95% CrI	Median Rt Difference Percentage Changes Comparing with Previous Policy Interval & 95% CrI
Before policy A	2.2469 (1.66 ,2.95)	
A→ B	1.9766 (1.54, 2.48)	0.12 (−0.18, 0.36)
B→ C	1.1133 (0.96, 1.28)	0.44 (0.28, 0.56)
C→ D	1.08100 (0.98, 1.19)	0.03 (−0.13, 0.18)
D→ E	1.0863 (1.06, 1.11)	−0.004 (−0.11, 0.09)
Beyond E	1.0397 (1.025, 1.05)	0.04 (0.02,0.07)

References:

1. Governor Brian P. Kemp Office of the Governor. 2020 Executive Orders. available online: https://gov.georgia.gov/executive-action/executive-orders/2020-executive-orders (accessed on August 13, 2020).
2. Shepherd, K. Teachers returned to a Georgia school district last week. 260 employees have already gone home to quarantine. available online: https://www.washingtonpost.com/nation/2020/08/04/school-outbreaks-reopening-georgia/ (accessed on March 25, 2021).
3. Bartow County. The State of Georgia Executive Order. available online: https://www.bartowga.org/CommissionerOffice/KEMP_EXECUTIVE_ORDER_ONGOING_GUIDANCE_REVIVE_GEORGIA_05-28-2020.pdf (accessed on July 13, 2020).
4. WSBTV.com News Staff. County-by-County: Plans for returning to school this fall. available online: https://www.wsbtv.com/news/local/county-by-county-plans-returning-school-this-fall/QJAYLUB4TFBPBCJCIMVZXUQGFY/ (accessed on March 25, 2021).
5. Catoosa County, Georgia. available online: https://www.catoosa.com/corona-covid-19 (accessed on July 13, 2020).
6. Chattooga County. Governor Brian Kemp's Executive Orders. available online: https://chattoogacounty.org/together-4-chattooga/ (accessed on July 13, 2020).

7. Dade County, Georgia. available online: https://www.facebook.com/DadeCountyGA/posts/565178144131796 (accessed on July 13, 2020).
8. Floyd County. Corona Virus Updates. available online: https://romefloyd.com/coronavirus-updates (accessed on July 13, 2020).
9. Gordon County. COVID-19 Information & Resources. available online: http://www.gordoncountychamber.com/covid-19 (accessed on July 13, 2020).
10. Haralson County. Association County Commissioners of Georgia & Georgia Municipal Association. Executive Order Issuing State-wide Shelter in Place to stop the spread of COVID-19 and the impact on local governments. available online: https://growthzonesitesprod.azureedge.net/wp-content/uploads/sites/771/2020/04/Summary-of-Executive-Order-Imposing-Statewide-Shelter-in-Place-as-it-Relates-to-Local-Government-2020-04-02-002-1.pdf. (accessed on July 13, 2020).
11. Paulding County. Ordinance 20-06 Termination of Chairman's declaration of a state of emergency. available online: https://www.paulding.gov/DocumentCenter/View/9790/Ordinance-20-06 (accessed on July 13, 2020).
12. Polk County College & Career Academy. Coronavirus/ COVID-19. available online: https://www.polk.k12.ga.us/PCCCA/News/psdcoronaviusinfo2020#sthash.a78CFqs7.5vNs4sCS.dpbs (accessed on July 13, 2020).
13. Walker County responds to COVID-19. Breaking down Governor Brian Kemp's COVID-19 "Minimum Operations" Executive Order. available online: https://walkercountyga.gov/tag/covid/ (accessed on July 13, 2020).
14. Cherokee County. Coronavirus (COVID-19). available online: https://www.cherokeega.com/_focus/corona-virus-plan.php (accessed on July 13, 2020).
15. Fannin County Georgia. available online: https://www.fannincountyga.com/ (accessed on July 13, 2020).
16. Gilmer County Government. Executive orders issued by the state of Georgia. available online: https://gilmercounty-ga.gov/ (accessed on July 13, 2020).
17. Pickens County Georgia. available online: https://pickenscountyga.gov/wp-content/uploads/2020/04/PickensCounty-SalonsBarberShops.pdf (accessed on July 13, 2020).
18. Joint resolution of Whitfield County and the cities of Dalton, Tunnel Hill, Varnell, and the Town of Cohutta limiting certain activities to mitigate the spread of COVID-19, and for other purposes. available online: https://www.whitfieldcountyga.com/COVID19News.pdf (accessed on July 13, 2020).
19. Banks County. available online: http://www.co.banks.ga.us/Home.html (accessed on July 13, 2020).
20. Dawson County Georgia. available online: https://www.dawsoncounty.org/administration (accessed on July 13, 2020).
21. Franklin County Georgia. The state of Georgia. Executive Order. available online: https://www.franklincountyga.gov/wp-content/uploads/2020/04/2020_04_23_ExecutiveOrder-Reviving-a-Healthy-Georgia.pdf (accessed on July 13, 2020).
22. Habersham County Georgia. COVID-19 Information and Updates. available online: https://www.habershamga.com/covid-19-information-and-updates.cfm (accessed on July 13, 2020).
23. Hall County Georgia. Ready Hall County. Coronavirus (COVID-19) Information. available online: https://www.hallcounty.org/186/READY-Hall-County (accessed on July 13, 2020).
24. Hart County. available online: at http://hartcountyga.gov/ (accessed on July 13, 2020).
25. Lumpkin County Georgia. Coronavirus. available online: https://www.lumpkincounty.gov/470/Coronavirus (accessed on July 13, 2020).
26. Stephens County Georgia. COVID-19 Resource Center. available online: https://www.stephenscountyga.com/covid-19-information-and-updates.cfm (accessed on July 13, 2020).
27. Towns County Hiawassee young Harris. Declaration of Judicial Emergency. available online: http://www.townscountyga.org/declaration-of-judicial-emergency.html (accessed on July 13, 2020).
28. Blairsville Union County. available online: http://www.unioncountyga.gov/ucoffices/ (accessed on July 13, 2020).
29. White County Georgia. available online: http://whitecounty.net/ (accessed on July 13, 2020).
30. The state of Georgia. Executive order. available online: https://s3.us-west-2.amazonaws.com/cobbcounty.org.if-us-west-2/prod/2020-06/06.11.20.01_0.pdf (accessed on June 25, 2020).
31. Douglas County board of health. Executive Order. available online: http://www.celebratedouglascounty.com/happenings/03_26_Douglas_County_Board_of_Health_COVD-19_ExecutiveOrder.pdf (accessed on June 25, 2020).
32. Fulton County Service Updates. available online: https://fultoncountyga.gov/ (accessed on June 25, 2020).
33. Clayton County Georgia. Coronavirus education/information. available online: https://www.claytoncountyga.gov/residents/coronavirus-education-information (accessed on June 27, 2020).
34. Local emergency order. available online: https://www.gwinnettcounty.com/static/departments/boc/pdf/Local%20Emergency%20Order%201-4%203%2027%2020.pdf (accessed on June 27, 2020).
35. An amended and restated resolution for the second declaration of a local state of emergency related to COVID-19; and for other purposes. available online: http://co.newton.ga.us/DocumentCenter/View/2827/2020-3-31-County-Emergency-Resolution- (accessed on June 27, 2020).
36. Rockdale County courthouse remains open for essential court business. available online: https://rockdalecountyga.gov/our-government/top-stories/ (accessed on June 27, 2020).

37. Executive order implementing "COVID-19: The path forward," A 30 day transition plan to reinforce pandemic mitigation measures and restore economic prosperity to Dekalb County. available online: https://www.dekalbcountyga.gov/sites/default/files/users/user715/Executive%20Order%2020-003.pdf. (accessed on June 27, 2020).
38. Butts County Georgia. Governor Issues Executive order to shelter in place for Georgia. available online: https://buttscountyga.com/executiveordergeorgia/ (accessed on July 13, 2020).
39. Carroll County Georgia. COVID-19 Update. available online: https://carrollcountyga.com/718/COVID-19-Update (accessed on July 13, 2020).
40. Coweta County Government. available online: https://www.facebook.com/cowetacounty/posts/3511433092208391 (accessed on July 13, 2020).
41. Fayette County. Coronavirus COVID-19 Information. available online: https://fayettecountyga.gov/coronavirus-information (accessed on June 27, 2020).
42. Henry County safer-at-home ordinance repealing ordinance no 20-01 and imposing shelter-in-place restrictions to control the spread of COVID-19. available online: https://www.co.henry.ga.us/Portals/0/pdf/Ordinance_20-02.Shelter-In-Place.pdf (accessed on July 13, 2020).
43. Meriwether County Georgia. COVID-19 Information. available online: https://www.meriwethercountyga.gov/CivicAlerts.aspx?AID=42 (accessed on July 13, 2020).
44. Pike County Georgia. COVID-19 executive order extended. available online: http://www.pikecountygeorgia.com/archives/8233-COVID-19-executive-order-extended.html (accessed on July 13, 2020).
45. Robert Brockman Bleckley County Commissioner. Declaration of state of emergency and curfew in Bleckley County, Georgia. available online: http://www.bleckley.org/public_documents/DECLARATION_OF_STATE_OF_EMERGENCY_3-24-2020.pdf (accessed on July 13, 2020).
46. Dodge County Georgia. available online: https://www.dodgecountyga.com/index.html (accessed on July 13, 2020).
47. Lawrens County Georgia. Gov. Kemp issuing a statewide shelter in place to stop the spread of COVID-19. available online: https://laurenscoga.org/CivicAlerts.aspx?AID=133 (accessed on July 13, 2020).
48. Montgomery County Georgia. Welcome Montgomery County, GA. available online: https://montgomerycountyga.gov/ (accessed on July 13, 2020).
49. Declaration of local state of emergency. available online: https://hawkinsville-pulaski.org/wp-content/uploads/2020/03/Signed-State-of-Emergency-3-23-2020.pdf (accessed on July 13, 2020).
50. Telfair County chamber. COVID-19 public notice. available online: http://www.telfairco.org (accessed on July 13, 2020).
51. Governor Brian Kemp. available online: https://www.facebook.com/GovKemp/photos/pcb.2973724649383569/2973724502716917/?type=3&theater (accessed on July 13, 2020).
52. State of Georgia County of Baldwin. A third emergency declaration by the Baldwin County board of commissioners, to provide for the health, safety, and welfare of the general public. available online: https://www.baldwincountyga.com/wp-content/uploads/2020/05/Baldwin-Emergency-Declaration-3.pdf (accessed on July 13, 2020).
53. Macon-Bibb County. Executive order. Setting expectations for public response to COVID-19. available online: https://www.maconbibb.us/wp-content/uploads/2020/07/Executed-face-covering-EO.pdf (accessed on July 13, 2020).
54. Crawford County. available online: https://www.crawfordcountyga.org/news/ (accessed on July 13, 2020).
55. Hancock County. Declaration of local emergency. available online: http://hancockcountyga.gov/wp-content/uploads/2020/03/CCE03242020.pdf. (accessed on July 13, 2020).
56. Gov. Kemp extends COVID-19 executive orders. available online: http://hhjonline.com/gov-kemp-extends-covid-executive-orders-p14026-95.htm. (accessed on July 13, 2020).
57. Jasper County. The state of Georgia. Executive order. available online: https://jaspercountyga.org/wp-content/uploads/2020/04/Announcement-of-State-Executive-Orders-4-3-2020.pdf. (accessed on July 13, 2020).
58. Jones County. available online: https://www.jonescountyga.org/wp-content/uploads/2020/03/COVID-Operations-Memo-Restricted-Access-FINAL-032120.pdf (accessed on July 13, 2020).
59. Monroe County. County announces re-opening schedule. available online: http://www.monroecoga.org/county-announces-re-opening-schedule/ (accessed on July 13, 2020).
60. Peach county. Executive Order. available online: https://www.peachcounty.net/userfiles/Order.pdf (accessed on July 13, 2020).
61. Putnam County sheriff Howard Sills issues COVID-19 statement on governors new orders. available online: https://www.msgrnews.com/article/putnam-county-sheriff-howard-sills-issues-covid-19-statement-governors-new-orders (accessed on July 13, 2020).
62. Twiggs County board of commission. available online: https://www.facebook.com/twiggscountyga/posts/124219025881575 (accessed on July 13, 2020).
63. Washington County. Recent news. available online: https://washingtoncountyga.gov/ (accessed on July 13, 2020).
64. Emergency ordinance adopted by commissioners. Burke County COVID-19. available online: https://burkecounty-ga.gov/index.php/content-pages/news-announcements/emergency-ordinance-adopted-by-commissioners. (accessed on July 16, 2020).

65. Columbia County. News. available online: https://www.columbiacountyga.gov/Home/Components/News/News/4709/4850 (accessed on July 16, 2020).
66. Declaration of a state of emergency. A declaration of a state of emergency Emanuel County and municipalities arising because of COVID-19: an ordinance. available online: https://www.emanuelco-ga.gov/civicalerts.aspx?aid=28 (accessed on July 16, 2020).
67. McDuffie County. The state of Georgia, Executive Order. available online: https://www.mcduffiecountysheriff.com/userfiles/04-02-20-01-1.pdf (accessed on July 16, 2020).
68. In the superior court of Richmond County. State of Georgia. Standing order for court proceedings. available online: https://www.augustaga.gov/DocumentCenter/View/13509/File-Stamped-Richmond-COVID-Standing-Order?bidId= (accessed on July 16, 2020).
69. COVID-19 alerts, information and updates. available online: https://www.tcsoga.com/stay-at-home-order.cfm (accessed on July 16, 2020).
70. The state of Georgia. The executive order. Executive order to ensure a safe & healthy Georgia. available online: https://www.warrencountyga.com/uploads/4/8/9/2/48921471/executive_order.pdf (accessed on July 16, 2020).
71. The state of Georgia. Executive order. Empowering a healthy Georgia. available online: https://chambermaster.blob.core.windows.net/userfiles/UserFiles/chambers/2812/CMS/COVID19/Exec-Order-Extended_07.15.20.01.pdf (accessed on July 16, 2020).
72. Coronavirus. Chattahoochee County halts public access to buildings, facilities through April 21 due to coronavirus. available online: https://www.wrbl.com/news/health/coronavirus/chattahoochee-county-halts-public-access-to-buildings-facilities-through-april-21-due-to-coronavirus/ (accessed on July 16, 2020).
73. Clay County board of commissioners. News and announcements. available online: https://www.claycountyga.net/ (accessed on July 16, 2020).
74. State of emergency. An ordinance for the declaration of a local state of emergency related to COVID-19; and for other purposes. available online: https://crispcountysheriff.com/coronavirus-covid-19/state-of-emergency (accessed on July 16, 2020).
75. Harris County community message on COVID-19. available online: https://harriscountyga.gov/wp-content/uploads/2020/03/Community-Message-on-COVID-19.pdf (accessed on July 16, 2020).
76. The state of Georgia. Executive order. Reviving a healthy Georgia. available online: https://www.marioncountyga.org/wp-content/uploads/2020/05/05.28.20.02.pdf (accessed on July 16, 2020).
77. An executive order imposing a public safety curfew. available online: https://www.scribd.com/document/469036136/Quitman-County-Public-Safety-Curfew#from_embed (accessed on July 16, 2020).
78. Executive order. available online: https://talbotcountyga.org/Documents/misc_docs_2020/executive_order.pdf (accessed on July 16, 2020).
79. Webster County declares local state of emergency. available online: https://www.wrbl.com/news/local-news/webster-county-declares-local-state-of-emergency/ (accessed on July 16, 2020).
80. Ben Hill County EMA. Executive Orders May 28, 2020. available online: https://benhillcountyema.com/2020/05/28/executive-orders-may-28-2020/ (accessed on June 24, 2020).
81. Berrien Chamber. COVID-19 Updates. available online: https://www.berrienchamber.com/covid-19-updates (accessed on June 24, 2020).
82. Brooks County. Georgia Emergency Management and Homeland Security Agency. available online: https://www.facebook.com/brookscountygeorgia/posts/4211864948827415 (accessed on June 24, 2020).
83. Cook County Georgia. Emergency Administrative order COVID-19 shelter in place curfew imposed. available online: https://cookcountyga.us/2020/03/25/emergency-administrative-order-covid-19-shelter-in-place-curfew-imposed/ (accessed on June 24, 2020).
84. Echols County Georgia. Local Emergency Purpose Order. available online: https://www.facebook.com/Echols.County/photos/a.306227265534/10158002588425535/?type=3&theater. (accessed on June 24, 2020).
85. Lanier County Board of Commissioners. State of Emergency. available online: https://www.lanierema.com/downloads/attach5/Lanier%20County%20LSOE-1.pdf. (accessed on June 24, 2020).
86. Lowndes County Georgia. Governor Kemp Issues Shelter in Place Order. available online: https://www.lowndescounty.com/CivicAlerts.aspx?AID=357. (accessed on June 24, 2020).
87. Tift County. Emergency Administrative Order. available online: https://drive.google.com/file/d/11aJ5hQxBu7DZpBEJx52MkVWfsjvZQMnl/view (accessed on June 24, 2020).
88. Coronavirus. Turner County expands local state of emergency. available online: https://www.walb.com/2020/03/24/turner-co-expands-local-state-emergency/ (accessed on June 25, 2020).
89. City of Calhoun GA. The state of Georgia Executive order. available online: https://www.cityofcalhounga.com/wpcontent/uploads/2020/06/06.11.20.01.pdfhttps://drive.google.com/file/d/1tyde7wEj2mFU4yVBIPl-yIBGTFmscbRV/view (accessed on June 24, 2020).
90. Joint Executive Order of the Mayor of the city of Albany and the chairman of the Dougherty County commission related to COVID-19. available online: http://dougherty.ga.us/filestorage/1800/379008/379647/RevisedOrder_032520.pdf (accessed on June 24, 2020).

91. Early County News. Gov. Kemp signs new COVID-19 executive order. available online: https://www.earlycountynews.com/articles/gov-kemp-signs-new-covid-19-executive-order/. (accessed on June 24, 2020).
92. Joint declaration of a state of emergency and emergency executive order of the chairman of the Grady County board of commissioners, mayor of the city of Cairo, and mayor of the city of Whigham. available online: https://www.cairogachamber.com/. (accessed on June 24, 2020).
93. First amendment to the executive order of the board of commissioner of Lee County, Georgia related to emergency responses to corona virus pandemic. available online: http://www.lee.ga.us/publicnotice/files/COVID_Executive_Order_Amendment_032420.pdf (accessed on June 24, 2020).
94. Mitchell EMC. An important update on COVID-19. available online: https://mitchellemc.com/ (accessed on June 24, 2020).
95. Board of commissioners of Seminole County. Georgia city council of Iron city, Georgia emergency declaration order. available online: http://seminolecountyga.com/SeminoleCountyEmergencyDeclarationOrderMarch252020.pdf. (accessed on June 24, 2020).
96. The state of Georgia executive order. available online: http://www.terrellcountygeorgia.com/EXECUTIVE_ORDER_CORONA_VIRUS.pdf (accessed on June 24, 2020).
97. Worth County GA. Covid-19 response information. available online: https://worthga.com/ (accessed on June 24, 2020).
98. Joint resolution declaring state of emergency in the county of Bryan, the city of Richmond Hill, Georgia, and the city of Pembroke, Georgia establishing the practice of certain recommendations from the CDC and Georgia department of public health in regards to the COVID-19 event. available online: https://www.bryancountyga.org/home/showdocument?id=8597 (accessed on June 4, 2020).
99. Camden County Georgia. COVID-19. available online: https://www.camdencountyga.gov/1076/COVID-19 (accessed on June 4, 2020).
100. Georgia department of public health Coastal health district. available online: https://www.gachd.org/?s=covid-19+executive+order (accessed on June 4, 2020).
101. News Release. Covid-19 Glynn County Non-essential business closures. available online: https://www.glynncounty.org/DocumentCenter/View/65936/20200324_GlynnCountyNon-EssentialBusinesClosures (accessed on June 4, 2020).
102. Long County board of commissioners. available online: https://www.facebook.com/photo?fbid=2560274134230908&set=pcb.2560274170897571 (accessed on June 4, 2020).
103. Appling County Emergency Management Agency. Appling County-COVID-19 information. available online: https://www.facebook.com/applingcountyema/posts/3924415820916852 (accessed on June 4, 2020).
104. district., G.d.o.p.h.s. available online: https://www.sehdph.org/category/news/ (accessed on June 5, 2020).
105. The city of Alma. A declaration of a state of emergency arising because of COVID-19; An ordinance taking immediate emergency measures. available online: https://www.cityofalmaga.gov/state_of_emergency_declaration/index.php (accessed on June 4, 2020).
106. Brantley County emergency management agency. available online: https://www.facebook.com/brantleyema/ (accessed on June 4, 2020).
107. A temporary ordinance that affirms the second declaration of a local state of emergency related to COVID-19; and for other purposes. available online: http://bullochcounty.net/wp-content/uploads/2020/04/COVID-19-Temporary-Curfew-Ordinance.pdf (accessed on June 5, 2020).
108. City of Metter. Press Release. available online: https://www.metter-candler.com/covid-19-resources_3_870297039.pdf (accessed on June 5, 2020).
109. Charlton County Georgia. Coronavirus COVID-19 information for all citizens. available online: https://charltoncountyga.us/418/Coronavirus-COVID-19-Information-for-all (accessed on June 5, 2020).
110. Clinch County. Local emergency purpose order. available online: https://clinchcountyga.gov/uploads/1/2/7/1/127184196/clinch_county_local_emergency_management_purpose_order.pdf (accessed on June 4, 2020).
111. Coffee County. Emergency administrative order. available online: https://coffeecountygov.com/Emergency%20Admin%20Order.pdf (accessed on June 4, 2020).
112. Evans County board of commissioners. Press release. available online: https://allongeorgia.com/evans-local-government/evans-county-shutters-businesses-with-executive-order/ (accessed on June 4, 2020).
113. Jeff Davis County board of commissioners. State of emergency. available online: https://nebula.wsimg.com/7a4952bef5559aba3ec336485b76120e?AccessKeyId=A41345E2884C0768E748&disposition=0&alloworigin=1 (accessed on June 4, 2020).
114. Pierce County Georgia. Our community & COVID-19. available online: https://pcgeorgia.com/covid-19/ (accessed on June 5, 2020).
115. Tattnall County commissioners. COVID-19 temporary closures and other actions. available online: https://www.facebook.com/tattnallcounty/photos/pcb.1551602735008756/1551602115008818/?type=3&theater (accessed on June 5, 2020).

116. Joint press release. Local government COVID-19 response. available online: http://www.toombscountyga.gov/wp-content/uploads/2020/03/Joint-Press-Release-Local-Government-COVID-19-Response-03.24.2020.pdf (accessed on June 5, 2020).
117. Waycross-Ware County Development authority. Ware county ordinance. available online: https://wwda.us/covid-19-resources/ware-county-ordinance/ (accessed on June 4, 2020).
118. Wayne County. Resolution declaring state of emergency in Wayne County, Georgia and herein established emergency protective orders to control the spread of covid-19. available online: https://www.waynecountyga.us/egov/documents/1585082616_66794.pdf (accessed on June 4, 2020).
119. An ordinance for the fifth declaration of a local state of emergency related to COVID-19; and for other purposes. available online: https://www.accgov.com/DocumentCenter/View/69444/Face-Cover-Ordinance---Signed-070820?bidId= (accessed on July 16, 2020).
120. Greene County message on COVID-19. available online: http://www.greenecountyga.gov/index.php?src=gendocs&ref=COVID-19%20Greene%20County%20Message&category=Government. (accessed on July 16, 2020).
121. The state of Georgia. Executive order.Empowering a Healthy Georgia. available online: https://www.jacksoncountygov.com/DocumentCenter/View/3070/Executive-Order-06112001?bidId= (accessed on July 16, 2020).
122. First amended ordinance for the declaration of a local state of emergency related to COVID-19; and for other purposes. available online: http://www.madisonga.com/DocumentCenter/View/2525/Ordiance---Declaration-of-Local-State-of-Emergency (accessed on July 16, 2020).
123. The state of Georgia. Executive order. available online: https://www.morgancountyga.gov/DocumentCenter/View/2668/06292001?bidId= (accessed on July 16, 2020).
124. Oconee County. The state of Georgia, Executive order. Reviving a healthy Georgia. available online: https://www.oconeecounty.com//DocumentCenter/View/10035/Governor-Kemps-Executive-Order-Reviving-a-Healthy-Georgia (accessed on July 16, 2020).
125. Oglethorpe County. The state of Georgia, Executive order. Executive order to ensure a safe & healthy Georgia. available online: http://www.accg.org/docs/Covid-19/EO-%20Order%20to%20Ensure%20Healthy%20GA.pdf (accessed on July 16, 2020).
126. Walton County Georgia. Gov. Kemp extends COVID-19 executive orders. available online: https://www.waltoncountyga.gov/CivicAlerts.aspx?AID=147 (accessed on July 16, 2020).
